# Molecular analyses of triple-negative breast cancer in the young and elderly

**DOI:** 10.1186/s13058-021-01392-0

**Published:** 2021-02-10

**Authors:** Mattias Aine, Ceren Boyaci, Johan Hartman, Jari Häkkinen, Shamik Mitra, Ana Bosch Campos, Emma Nimeus, Anna Ehinger, Johan Vallon-Christersson, Åke Borg, Johan Staaf

**Affiliations:** 1 Division of Oncology, Department of Clinical Sciences Lund, Lund University, Medicon Village, SE-22381 Lund, Sweden; 2 Department of Clinical Pathology and Cytology, Karolinska University Laboratory, Stockholm, Sweden; 3 Department of Oncology and Pathology, Karolinska Institute, Stockholm, Sweden; 4 Division of Clinical Genetics, Department of Laboratory Medicine, Lund University, Lund, Sweden; 5 Division of Surgery, Department of Clinical Sciences, Lund University, Lund, Sweden; 6 Department of Genetics and Pathology, Laboratory Medicine, Region Skåne, Lund, Sweden

**Keywords:** Triple-negative breast cancer, Age at diagnosis, Gene expression, Mutations, Mutational signatures, PD-L1, TILs, Patient outcome

## Abstract

**Background:**

Breast cancer in young adults has been implicated with a worse outcome. Analyses of genomic traits associated with age have been heterogenous, likely because of an incomplete accounting for underlying molecular subtypes. We aimed to resolve whether triple-negative breast cancer (TNBC) in younger versus older patients represent similar or different molecular diseases in the context of genetic and transcriptional subtypes and immune cell infiltration.

**Patients and methods:**

In total, 237 patients from a reported population-based south Swedish TNBC cohort profiled by RNA sequencing and whole-genome sequencing (WGS) were included. Patients were binned in 10-year intervals. Complimentary PD-L1 and CD20 immunohistochemistry and estimation of tumor-infiltrating lymphocytes (TILs) were performed. Cases were analyzed for differences in patient outcome, genomic, transcriptional, and immune landscape features versus age at diagnosis. Additionally, 560 public WGS breast cancer profiles were used for validation.

**Results:**

Median age at diagnosis was 62 years (range 26–91). Age was not associated with invasive disease-free survival or overall survival after adjuvant chemotherapy. Among the *BRCA1*-deficient cases (82/237), 90% were diagnosed before the age of 70 and were predominantly of the basal-like subtype. In the full TNBC cohort, reported associations of patient age with changes in Ki67 expression, *PIK3CA* mutations, and a luminal androgen receptor subtype were confirmed. Within DNA repair deficiency or gene expression defined molecular subgroups, age-related alterations in, e.g., overall gene expression, immune cell marker gene expression, genetic mutational and rearrangement signatures, amount of copy number alterations, and tumor mutational burden did, however, not appear distinct. Similar non-significant associations for genetic alterations with age were obtained for other breast cancer subgroups in public WGS data. Consistent with age-related immunosenescence, TIL counts decreased linearly with patient age across different genetic TNBC subtypes.

**Conclusions:**

Age-related alterations in TNBC, as well as breast cancer in general, need to be viewed in the context of underlying genomic phenotypes. Based on this notion, age at diagnosis alone does not appear to provide an additional layer of biological complexity above that of proposed genetic and transcriptional phenotypes of TNBC. Consequently, treatment decisions should be less influenced by age and more driven by tumor biology.

**Supplementary Information:**

The online version contains supplementary material available at 10.1186/s13058-021-01392-0.

## Introduction

Triple-negative breast cancer (TNBC; defined by ER, PR, and *HER2/ERBB2*-negativity) is a clinically defined subgroup of breast cancer, constituting approximately 10–15% of cancers in Western countries. TNBC is overrepresented among younger women, African-American women, and women with inherited mutations in high-penetrance breast cancer susceptibility genes [1, 2]. TNBC tumors are highly heterogeneous on the molecular level, involving differences in genetic features, germline alterations (e.g., *BRCA1* mutations), DNA repair deficiency, epigenetic alterations, gene expression patterns, but also morphological features [3–9]. Several of these features have been associated with prognosis and/or therapy response in TNBC patients. Despite the overrepresentation in younger women, large registry studies have suggested that young age (< 40 years) is not an independent risk factor in women with TNBC when adjusted for other prognostic variables [10–14], in contrast to other breast cancer subgroups [15].

Previous studies have attempted to address the molecular landscape of breast cancer in the context of patient age [16–21], with the recent study by Ma et al. focusing specifically on TNBC [19]. Overall in breast cancer, results concerning specific molecular traits or patterns associated with age at diagnosis appear heterogeneous. This may in part be due to inconsistent age group definitions between studies and that highly selected cohorts have often been used for molecular analyses. Concerning the latter, analyses have often not been performed within a relevant clinical subgroup or molecular phenotype, or that appropriate adjustments for other important clinical or molecular disease parameters have not been performed. As an example of the latter, an initial study by Anders et al. [21] reported transcriptional differences in breast cancer associated with patient age that subsequently disappeared when later correcting for clinical and proposed molecular subtypes [17]. Considering these common confounding factors, a more complete understanding of whether age at diagnosis in breast cancer is intrinsically linked to specific genetic, epigenetic, and transcriptional differences requires studies to be performed in representative population-based cohorts that account also for the relevant underlying genetic and transcriptional phenotypes of TNBC.

In the current study, we aimed to resolve whether TNBC in younger versus elderly patients represent similar or different molecular diseases in the context of proposed genetic and transcriptional TNBC subtypes. To this end, we used comprehensive whole-genome sequencing (WGS) data, RNA sequencing (RNAseq), and in situ immunohistochemistry data from 237 TNBC patients recruited from a population-based study in south Sweden [9]. To further generalize specific findings, we also analyzed an additional 560 reported WGS analyzed cases representative of all molecular breast cancer subtypes.

## Methods

### Ethics approval and consent to participate

All included patients were enrolled in the Sweden Cancerome Analysis Network – Breast (SCAN-B) study (ClinicalTrials.gov ID NCT02306096) [22–24], approved by the Regional Ethical Review Board in Lund, Sweden (Registration numbers 2009/658, 2010/383, 2012/58, 2016/742, 2018/267, and 2019/01252) as previously described [9]. All patients provided written informed consent prior to enrollment. All analyses were performed in accordance with local and international regulations for research ethics in human subject research.

### Unselected population-based TNBC cohort

A previously reported unselected population-based TNBC cohort comprised of 237 patients analyzed by RNAseq and WGS formed the study material [9]. The cohort is hereafter referred to as SCAN-B. Clinicopathological characteristics for included patients versus stratified age at diagnosis (10-year intervals, < 40, 40–50, 50–60, 60–70, 70–80, and ≥ 80 years) are summarized in Table 1. The analyzed patient cohort has previously been shown to be representative of the underlying healthcare population from which it was recruited based on comparison with the Swedish national breast cancer quality registry (NKBC) [9].

**Table 1:** Patient characteristics and clinicopathological variables of the study cohorts

	All patients	Patients < 40 years	Patients 40–50 years	Patients 50–60 years	Patients 60–70 years	Patients 70–80 years	Patients ≥ 80 years	Statistical difference between age groups^E^
*N*	237	28	24	55	53	40	37	
ER IHC % (1–10%) ^A^	12.3%	10.7%	16.7%	9.3%	15.1%	10.3%	13.5%	*p* = 1.0
Tumor size > 20 mm	49.4%	46.4%	37.5%	40.0%	50.9%	42.5%	78.4%	*p* = 0.09
Grade 3 (%)	87.9%	96.3%	100%	94.3%	78.4%	80%	86.5%	*p* = 0.29
Median Ki67%	70	87	78	70	70	58	60	
Node positive (%)	34.6%	37.0%	29.2%	32.1%	34.0%	37.5%	37.8%	*p* = 1.0
Adjuvant chemotherapy (%)	72.8%	100%	100%	94.5%	84.9%	59.0%	0%	*p* = 4e−15
Outcome								
Death events (%)	26.6%	17.9%	0%	12.7%	32.1%	27.5%	62.2%	*p* = 3e−6
IDFS events (%)	32.5%	25.0%	12.5%	16.4%	39.6%	35.0%	62.2%	*p* = 0.0007
Distant metastases (%)	20.7%	21.4%	8.3%	14.5%	28.3%	20.0%	27.0%	*p* = 1.0
*BRCA1*-germline (%) ^B^	8.0%	28.6%	4.2%	12.7%	1.9%	5.0%	0%	*p* = 0.003
*BRCA1* status								
*BRCA1*-null ^C^ (%)	10.5%	32.1%	4.2%	16.4%	7.5%	5.0%	0%	*p* = 0.006
*BRCA1* hypermethylation (%)	24.1%	50.0%	41.7%	21.8%	28.3%	10.0%	5.4%	*p* = 0.001
*BRCA1* wildtype ^D^	65.4%	17.9%	54.1%	61.8%	64.2%	85%	94.6%	*p* = 2e−8
HRD status								*p* = 1e−6
HRDetect-high (%)	58.6%	92.9%	83.3′%	67.3%	60.4%	27.5%	35.1%	
HRDetect-intermediate (%)	5.5%	0%	4.2%	5.5%	1.9%	5.0%	16.2%	
HRDetect-low (%)	35.9%	7.1%	12.5%	27.3%	37.7%	67.5%	48.6%	
PD-L1 positivity (%)	51.8%	65.4%	54.5%	62.7%	46.0%	40.0%	44.1%	*p* = 1.0
TILs (%)								*p* = 1.0
< 30%	61.5%	46.2%	40.9%	51.0%	73.5%	76.5%	69.7%	
30–50%	17.8%	19.2%	31.8%	24.5%	10.2%	11.8%	15.2%	
> 50%	20.7%	34.6%	27.3%	24.5%	16.3%	11.8%	15.2%	
PAM50 subtypes (%)								*p* = 0.001
Basal-like	79.9%	100%	95.7%	94.3%	73.5%	57.1%	63.9%	
HER2-enriched	14.7%	0%	4.3%	1.9%	16.3%	28.6%	36.1%	
Luminal A	1.3%	0%	0%	0%	2.0%	5.7%	0%	
Luminal B	0.4%	0%	0%	0%	0%	2.9%	0%	
Normal-like	3.6%	0%	0%	3.8%	8.2%	5.7%	0%	
TNBC subtypes (%)								*p* = 0.22
BL1	20.2%	14.3%	26.1%	26.4%	20.4%	14.7%	16.7%	
BL2	9.9%	10.7%	8.7%	11.3%	10.2%	5.9%	11.1%	
IM	20.2%	25.0%	21.7%	28.3%	14.3%	17.6%	13.9%	
LAR	13.0%	0%	0%	3.8%	16.3%	23.5%	30.6%	
M	18.4%	17.9%	21.7%	18.9%	20.4%	8.8%	22.2%	
MSL	6.3%	7.1%	13.0%	1.9%	4.1%	17.6%	0%	
UNS	12.1%	25.0%	8.7%	9.4%	14.3%	11.8%	5.6%	
IntClust10 subtypes								*p* = 0.07
1	0.9%	0%	4.3%	0%	0%	0%	2.8%	
10	64.7%	89.3%	87.0%	79.2%	57.1%	40.0%	44.4%	
3	2.2%	0%	4.3%	1.9%	2.0%	5.7%	0%	
4	25.0%	3.6%	4.3%	17.0%	30.6%	40.0%	44.4%	
5	0.9%	0%	0%	0%	2.0%	2.9%	0%	
8	0.4%	0%	0%	0%	0%	2.9%	0%	
9	5.8%	7.1.%	0%	1.9%	8.2%	8.6%	8.3%	

Proportions calculated excluding missing data. Groups are defined as, e.g., ≥ 40 and < 50

^A^Proportion of cases with an ER IHC staining of 1–10%, which is classified as ER-negative in Sweden

^B^Germline alteration according to WGS analysis

^C^*BRCA1*-null: germline and/or biallelic inactivation of *BRCA1* determined by WGS

^D^Patients that are not defined as *BRCA1*-null or show somatic *BRCA1* promoter hypermethylation based on available data for the study

^E^Chi-square test, with multiple testing correction by Bonferroni adjustment (*n* = 18 tests)

### Copy number and mutational analyses

From existing WGS data [9], we extracted calls of copy number alteration, tumor ploidy, loss of heterozygosity (LOH), breast cancer driver gene mutation calls, and genome-wide mutational and rearrangement signatures [3, 9]. DNA promoter hypermethylation status for *BRCA1* and *RAD51C* were obtained from [9]. *BRCA1*-null, *BRCA2*-null, and *PALB2*-null status was defined as either a somatic or germline loss of function variant with LOH, or a loss of function germline mutation only [9]. *BRCA1* deficiency was defined as either *BRCA1*-null or *BRCA1* promoter hypermethylation, as these are mutually exclusive and impart an identical genomic phenotype in TNBC [25]. As estimates of homologous recombination deficiency (HRD), we used the WGS-based HRDetect [26] and the copy number based HRD score (*genomic scars*) [27] classifications available from [9]. In addition, we calculated weighted genomic instability index scores [28], and mutant-allele tumor heterogeneity scores [29] as outlined in original studies.

Mutational signatures were refitted for specific group analyses using all substitutions with a PASS filter flag from the original study [9] using the latest version of the SigFit algorithm [30] with default parameters, except for using 8000 iterations. Only the signatures reported in our original WGS study [9] were refitted. Tumor mutational burden was calculated as the sum of somatic substitutions and indels per Mb sequence. Publicly available mutational and copy number data from 560 breast cancers of all subtypes analyzed by WGS and RNAseq were obtained from Nik-Zainal et al. [3]. This cohort is hereafter referred to as Nik-Zainal.

### Gene expression analyses

Processed RNA sequencing data (fragments per kilobase million, FPKM) for 232 SCAN-B cases, including gene expression subtype classifications of PAM50 using a nearest centroid classifier (*n* = 224 obtained from [31]), IntClust 10 (*n* = 224) [32, 33], and reported TNBC subtypes (*n* = 223) [6, 34] were obtained from [9, 31]. Supervised Significance Analysis of Microarrays (SAM) analysis was performed on FPKM data for the 232 cases after: (i) offsetting all FPKM values with + 0.1, (ii) log2 transformation, and (iii) mean-centering. Pathway analysis was performed using the PANTHER Classification System (http://pantherdb.org/geneListAnalysis.do) and the overrepresentation test application. Default settings were used, and gene ontology terms with a false discovery rate (FDR) adjusted Fisher’s exact test *p* < 0.05 were considered significant. Principal component analysis was performed using the R swamp package (ver 1.5.1) and all available refseq genes [35]. RNAseq-based immune cell deconvolution was obtained for 230 cases using CIBERSORTx [36] as described [25].

### PD-L1 and CD20 immunohistochemistry and tumor-infiltrating lymphocyte scoring (TILs)

PD-L1 immunohistochemistry using the SP-142 antibody (Roche) was performed on a tissue microarray including 218 SCAN-B tumors (two 1 mm cores / tumor) on a Ventana instrument (Roche) according to the manufacturer’s recommendations. PD-L1 assessment was performed according to antibody instructions on immune cells by a board-certified breast cancer pathologist, using a ≥ 1% cut-off for positivity. Cases negative in both TMA cores were set to score 0. CD20 immunohistochemistry was performed on TMA slides using the CD20 L26 clone (Dako/Agilent cat no M0755) with a 1:500 dilution incubated 30 min at room temperature. Deparaffinization and antigen retrieval was performed using the Dako PT-Link buffer. Staining was performed using the Dako Envision™ Flex K8010 kit in an Autostainer Plus (Dako/Agilent) instrument. Scoring was performed by a breast cancer pathologist into four groups (0,1,2,3) based on presence of stained cells from low to high.

Tumor-infiltrating lymphocytes (TILs) were scored on available whole section formalin-fixed paraffin-embedded hemotoxylin and eosin-stained slides by a board-certified breast cancer pathologist and summarized as a percentage per sample. Scoring was performed according to the international consensus scoring recommendations of the International Immuno-Oncology Biomarker Working Group on Breast Cancer (www.tilsinbreastcancer.org). When multiple slides were available, per patient scores were averaged. All pathology scorings were performed blinded to downstream analyses.

### Neoantigen prediction

NeoPredPipe [37] was used to predict putative neoantigens with substitution mutation calls provided by CaVEMan (https://cancerit.github.io/CaVEMan/) and HLA typing done with Polysolver [38] as input, as detailed in Glodzik et al. [25].

### Survival analyses and statistical methods

Survival analyses were performed in R (ver 3.6.0) using the survival package with overall survival (OS), invasive disease-free survival (IDFS), or distant relapse-free interval (DRFI), as endpoints defined according with the STEEP criteria [39]. Hazard ratios were calculated through univariable Cox regression and verified to fulfill assumptions for proportional hazards. Survival curves were compared using Kaplan-Meier estimates and the log-rank test. Survival analyses were performed using the 149 eligible cases (62.8%) from the 237-sample cohort treated with standard-of-care adjuvant chemotherapy according to national guidelines (in 96% of cases a FEC-based [combination of 5 fluorouracil, epirubicin, and cyclophosphamide] treatment ± a taxane). Full details on the exclusion criteria for outcome analysis and individual patient treatments are available in [9]. Trends of decreasing or increasing estimates versus age at diagnosis were tested using linear regression modeling with age as a continuous variable. All *p* values reported from statistical tests are two-sided if not otherwise specified. Box-plot elements corresponds to (i) center line = median, (ii) box limits = upper and lower quartiles, and (iii) whiskers = 1.5× interquartile range.

### Data availability statement

Genomic data used in the current study is available in open repositories as described in the original studies.

## Results

**Fig. 1:**
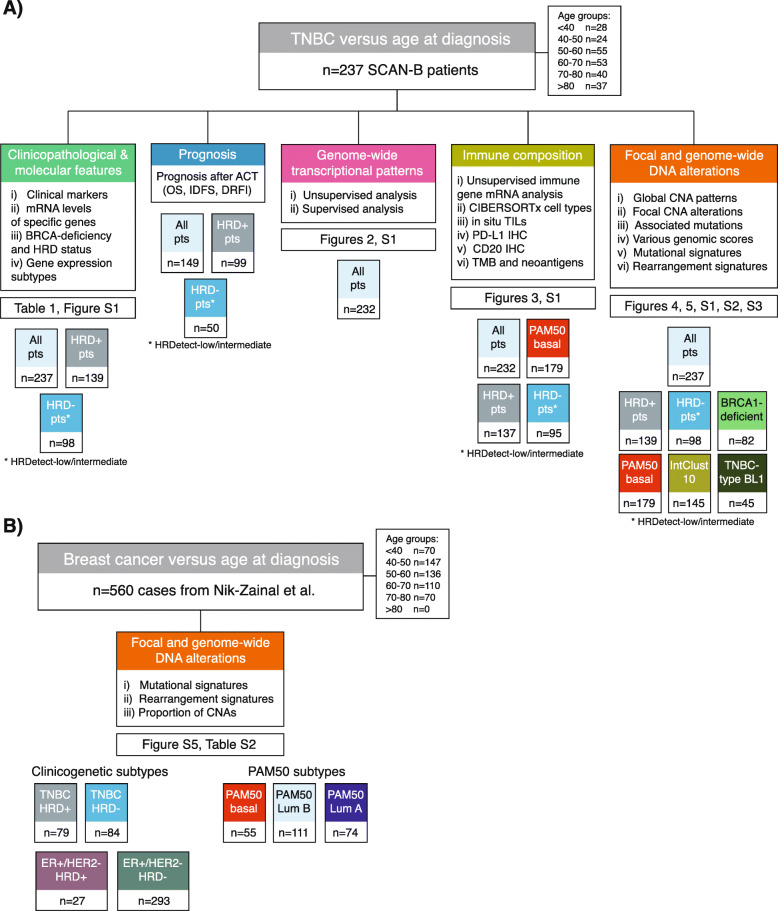
Study scheme. **a** Analyses performed in the SCAN-B TNBC cohort together with investigated main sample groups. **b** Analyses performed in the external Nik-Zainal et al. [3] cohort together with investigated main sample groups. In both panels, sample size numbers for patient groups refer to the largest set of patients available for at least one of the specified analyses. Specific sample size numbers are provided in the detailed results and the “Methods” section. References to the main figures and tables presenting results are provided for each analysis. HRD+: HRDetect-high, HRD-: HRDetect-low, TILs: tumor-infiltrating lymphocytes, TMB: tumor mutational burden, CNA: copy number alteration, Lum A: Luminal A, Lum B: Luminal B

An outline of the study, including performed analyses and sample group sizes, is shown in Fig. 1.

### Clinicopathological differences between young and old TNBC patients

In the SCAN-B cohort (*n* = 237), the median age at diagnosis was 62 years with a range of 26–91 years. Clinicopathological and molecular characteristics for cases are summarized in Table 1 for patients stratified into six age groups based on 10-year intervals; < 40, 40–50, 50–60, 60–70, 70–80, and ≥ 80 years. No statistical difference in ER immunohistochemistry levels (< 1% stained cells versus 1–10% stained cells) was observed between the six age groups (chi-square test *p* = 0.91, Table 1). This finding was supported by *ESR1* gene expression levels (FPKM) that did also not show significant differences between the six groups (Kruskal-Wallis test *p* = 0.34, Additional file 1A). Similarly, no statistically significant difference in *ESR1* FPKM levels versus age groups were found in patient subgroups defined by HRD status (HRDetect-high or low/intermediate) (Additional file 1A). In contrast, Ki67 immunohistochemistry levels, as well as Ki67 (*MKI67*) mRNA expression, were overall higher in younger patients with a decreasing trend with increasing age (Kruskal-Wallis *p* = 4e−05 and *p* = 0.0009, respectively, Additional file 1B-C). When considering the genetic background of HR deficiency (HRD), this decrease in Ki67 gene expression was, however, only statistically significant in HRDetect-low/intermediate patients (Kruskal-Wallis *p* = 0.03, linear regression *p* = 0.09, Additional file 1D-E). There was no statistical difference in the estimated tumor cell content by WGS between the six age groups that could explain the RNAseq observation (Kruskal-Wallis *p* = 0.15, Additional file 1F). Overall, analysis of the molecular and clinicopathological features listed in Table 1 revealed that the strongest associations with the stratified age groups were related to BRCA deficiency, HRD, and PAM50 subtypes.

### Age at diagnosis is not associated with outcome after adjuvant chemotherapy in TNBC

To test the association of age at diagnosis with outcome after adjuvant standard-of-care chemotherapy, we analyzed the 149 treated SCAN-B patients using OS, IDFS, and DRFI as clinical endpoints. Three different clinical endpoints were assessed to provide the most comprehensive view, as both OS and IDFS include death from other causes, for which higher age is a risk factor. For this patient subset, the median age at diagnosis was 56 years (range = 27–76 years). Univariate Cox regression analysis using patient age (years) as covariate did not reveal significant hazard ratios for any of the three clinical endpoints (Table 2). This non-significant finding was repeated also in HRD-high (HRDetect-high) patients, as well as HRD-low (HRDetect-low/intermediate) patients (Table 2). Univariate Cox regression using the six age groups did also not show any statistically significant results when tested using all chemotherapy-treated patients (all Cox regression *p* values > 0.05 for all clinical endpoints).

**Table 2:** Results of univariate Cox regression survival analysis in patients treated with adjuvant chemotherapy

Subset of patients	*N*	OS	IDFS	DRFI
All patients	149	HR = 1.04, 95% CI = 0.996–1.078, *p* = 0.08	HR = 1.02, 95% CI = 0.991–1.05, *p* = 0.18	HR = 1, 95% CI = 0.967–1.036, *p* = 0.98
HRDetect-high	99	HR = 1.046, 95% CI = 0.989–1.106, *p* = 0.12	HR = 1.008, 95% CI = 0.968–1.049, *p* = 0.71	HR = 0.977, 95% CI = 0.928–1.028, *p* = 0.365
HRDetect-low/intermediate	50	HR = 1.0, 95% CI = 0.938–1.067, *p* = 0.99	HR = 1.003, 95% CI = 0.954–1.055, *p* = 0.90	HR = 0.983, 95% CI = 0.928–1.042, *p* = 0.565

*OS* overall survival, *IDFS* invasive disease-free survival, *DRFI* distant relapse-free interval, *HR* hazard ratio, *CI* confidence interval

### Age at diagnosis in TNBC subgroups defined by BRCA1 and DNA repair deficiency

**Fig. 2:**
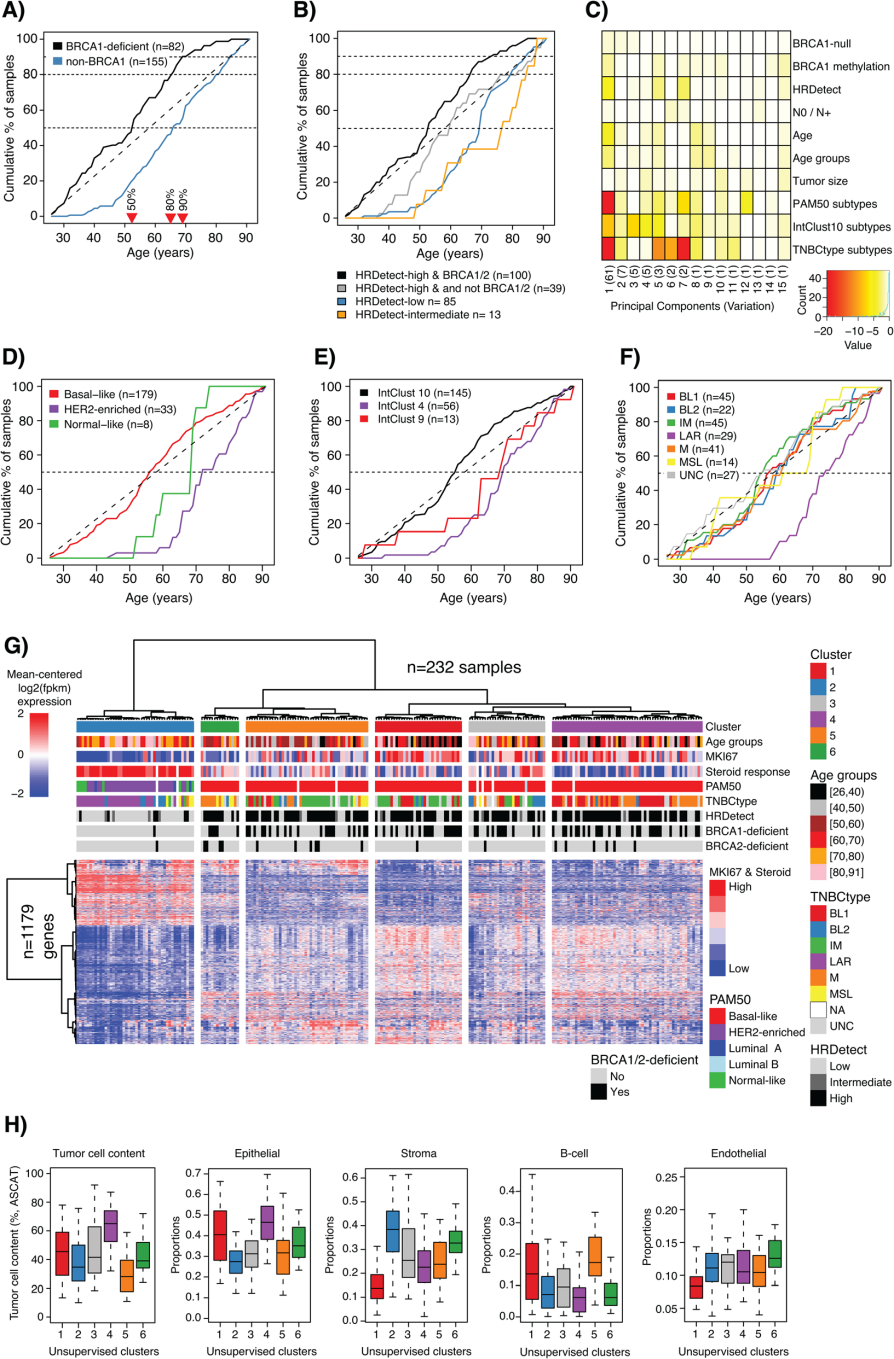
Patient age versus *BRCA1* deficiency and gene expression subtypes in TNBC. **a** Cumulative proportion of patients with *BRCA1* deficiency (*BRCA1* hypermethylation or *BRCA1*-null tumors) and non-*BRCA1*-deficient patients versus age at diagnosis. Red triangles indicate age at diagnosis for 50%, 80%, and 90% of *BRCA1*-deficient patients. **b** Cumulative proportions of patients in HRDetect groups versus age at diagnosis. **c** Principal component analysis of gene expression data for 232 SCAN-B cases using 19,102 RefSeq genes and different molecular and clinicopathological factors, including age at diagnosis (years: Age) and stratified age groups (10-year intervals: Age groups). **d** Cumulative proportions of PAM50 subtypes versus age at diagnosis. **e** Cumulative proportions of IntClust 10 subtypes versus age at diagnosis. **f** Cumulative proportions of TNBCtype subtypes versus age at diagnosis. **g** Heatmap of 1179 genes differentially expressed between six 10-year interval age groups in 232 SCAN-B cases. Hierarchical clustering of cases (columns) and genes (rows) were performed using Pearson correlation as distance metric and ward.D linkage on mean-centered log2 transformed data with an offset of 0.1. The six top clusters were identified and labeled. MKI67: Ki67. Steroid response: Scores according to the steroid response metagene [40]. **h** From left to right: Estimations of tumor cell content from WGS (ASCAT method), epithelial, stromal, B cell lymphocyte, and endothelial cell proportions from CIBERSORTx versus the hierarchical clusters in **g**. For age group definitions, “[” equals ≥, “)” equals <, and “]” equals ≤ for the value specified next to it

Younger patients showed higher proportions of *BRCA1* germline alterations, *BRCA1*-null tumors, and HRDetect-high tumors (a proxy for HRD) (Table 1). Strikingly, in patients < 40 years, 82.1% had a *BRCA1*-deficient phenotype (*BRCA1*-null tumor or somatic *BRCA1* promoter hypermethylation). In older patients, only 4.6% of patients ≥60 years had a *BRCA1*-null tumor, while 16.2% showed *BRCA1* promoter hypermethylation. For patients ≥ 70 years, corresponding values were 2.6% and 7.8%. We have previously shown the genomic equivalency of the phenotypes associated with *BRCA1* inactivation by DNA methylation or mutations [25]. The higher proportion of hypermethylated cases in older patients would suggest that a HRD phenotype brought on by epigenetic silencing represents a more long-tailed process compared to the same phenotype induced by germline alterations. However, clinical testing in known breast cancer families may bias this view. Among the 237 SCAN-B patients, 46 patients had undergone clinical germline screening with nine *BRCA1* positive cases. WGS analysis identified 10 additional cases with germline *BRCA1* alterations and also six cases with biallelic somatic inactivation [9]. Due to small sample groups, it could not be determined whether clinically screened patients with germline *BRCA1* alterations had different clinicopathological characteristics or different variant distribution compared to germline cases detected by WGS. While *BRCA1* germline-screened patients were notably younger (potentially due to participation in screening programs or presence of other unknown risk factors not known to this study), hypermethylated patients had similar age at diagnosis as *BRCA1* germline and somatic patients detected by WGS (Additional file 1G). This suggests a potentially similar intrinsic pace of tumor development for hypermethylated and mutation inactivated cases deserving validation in larger cohorts. Figure 2a shows the cumulative summary of *BRCA1* inactivated cases by mutation or hypermethylation versus age. It illustrates that 80% of detected *BRCA1* alterations occur before the age of 65, and 90% of alterations in patients ≤ 69 years. Similarly, for patient subsets defined by DNA repair deficiency, patients with HRD-high tumors (assessed by HRDetect) showed a trend towards a younger age at diagnosis compared to non-HRD patients (Fig. 2b).

### Transcriptional breast cancer subtypes versus TNBC patient age

To investigate the impact of patient age on the overall transcriptional variation in TNBC, we first performed an unsupervised principal component analysis, including different clinicopathological and molecular variables, and 19,102 refseq genes from 232 SCAN-B cases with matching gene expression data and classifications (Fig. 2c). Neither when used as a continuous variable (years) or stratified into six age groups could age strongly capture transcriptional variation in the cohort. In contrast, we found that specific gene expression subtypes in the PAM50, IntClust 10, and TNBC (TNBCtype) classification schemes differed significantly between age groups (Table 1) and appear to capture true transcriptional variation (Fig. 2c). Cumulative subtype plots versus patient age further illustrate that non-basal-like (PAM50), IntClust 4 (IntClust 10), and the luminal androgen receptor (LAR) (TNBCtype) subtypes are predominantly observed in older TNBC patients, increasing rapidly in patients aged 60 or more (Fig. 2d–f). Consistently, the LAR subtype has been previously associated with higher age at diagnosis and expression of, e.g., the androgen receptor (*AR*) [6, 19]. Across age groups for all SCAN-B patients, *AR* gene expression increased with higher age (Kruskal-Wallis *p* = 0.025), but the increase in expression was restricted to HRDetect-low/intermediate cases when the analysis was substratified (Kruskal-Wallis *p* = 0.07) (Additional file 1H). A similar result was obtained when using a six-gene (*AGR2*, *SLC44A4*, *TBC1D9*, *FOXA1*, *GATA3*, and *CA12*) breast cancer steroid response module [40] (Kruskal-Wallis *p* = 0.01 across all patients, *p* = 0.94 in HRDetect-high patients, and *p* = 0.02 in HRDetect-low/intermediate patients) (Additional file 1I). Exclusion of LAR classified cases in HRDetect-low/intermediate cases further reduced the association for both *AR* gene expression and the steroid response module with patient age (Additional file 1H-I). Additionally, within the 29 LAR subtyped samples, there was no statistical trend of changes in *AR* mRNA expression with stratified patient age (Kruskal-Wallis *p* = 0.41) (Additional file S1H).

In summary, we found that while a higher age at diagnosis was significantly associated with steroid response module and AR expression as well as a non-HRD phenotype, these observations are likely driven by these samples belonging to the LAR molecular subgroup rather than age per se.

### Age at diagnosis does not by itself represent a distinct gene signature in TNBC

To test whether an age-related gene expression signal exists in TNBC, we performed a supervised multiclass SAM analysis using the stratified age groups as class labels and the top 10,000 most variable refseq genes in the cohort as input. At a false discovery rate (FDR) of *p* = 0.01, 1179 genes were differentially expressed between the groups. Of these, 528 had a maximum median absolute difference in log2 expression > 1 across all groups, but only 76 had an absolute log2 expression difference > 2, indicating that the significant transcriptional alterations detected between groups are in the lower fold-change range. Pathway enrichment analysis of the 528 genes identified almost exclusively cell cycle-related gene ontology biological processes (Additional file 2). This is consistent with the previous observation of different Ki67 expression levels across the age groups. Hierarchical clustering of all 1179 age-associated genes across samples illustrated that the association with age groups appeared weak (Fig. 2g). To test this, we cut the dendrogram at the level of six sample clusters and tested the associations with our stratified age categories as well as proposed gene expression subtypes (PAM50, TNBCtype) and genetic subtypes (HRDetect). The analysis revealed a stronger overall association with the molecularly defined entities rather than patient age, even though the genes were preselected for their association with the latter (Fig. 2g, Additional file 1 J-L). Extending the analysis beyond transcriptomic and genetic subtypes, the clusters defined by age-related gene expression also differed with respect to differences in the tumor microenvironment, illustrated by estimates of tumor cell content from WGS and in silico estimated proportions of stromal and immune cell content by CIBERSORTx (Fig. 2h).

### Immune cell landscape, tumor mutational burden, and expressed neoantigens in TNBC with respect to age at diagnosis

To analyze the immune cell landscape in the context of age at diagnosis, we used a combination of in silico deconvolution of RNAseq data based on the CIBERSORTx method, in situ analyses of PD-L1 and CD20 protein expression, whole-slide hemotoxylin and eosin-stained TIL infiltration estimates, and neoantigen expression and tumor mutational burden (TMB) estimates from WGS. Analyses were performed in the complete SCAN-B cohort, HRDetect-high cases only, HRDetect-low only, and PAM50 basal-like cases (other molecular subtypes were not large enough to allow 10-year age binning).

**Fig. 3:**
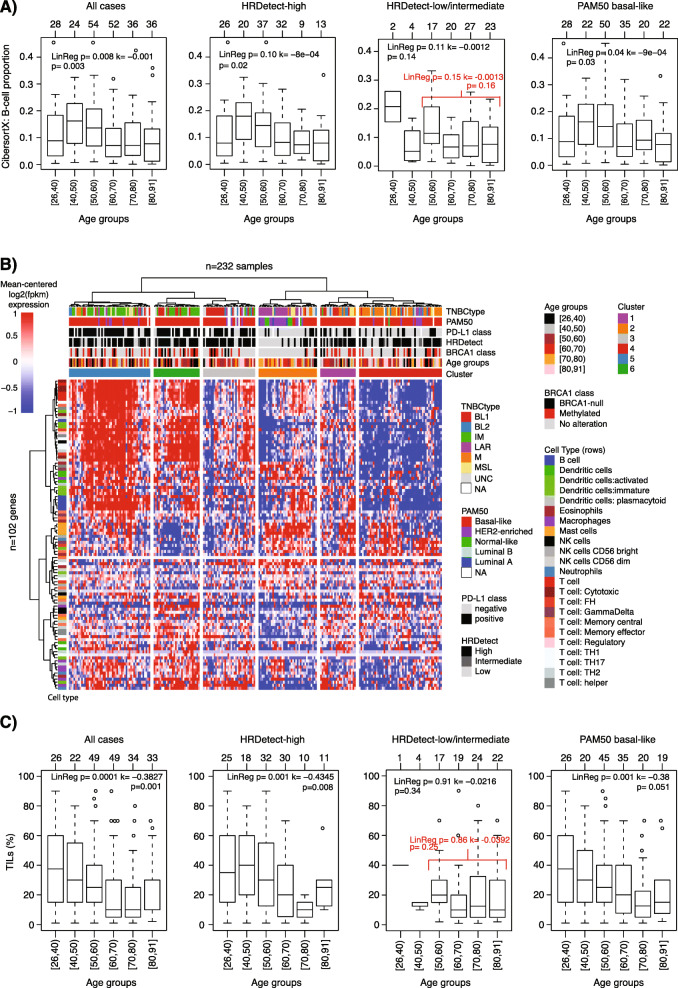
Immune cell landscape of TNBC with respect to age at diagnosis. **a** CIBERSORTx estimated B cell proportions per sample versus stratified age groups in all cases (left), HRDetect-high, HRDetect-low, and PAM50 basal-like cases (right). Top axes indicate group sizes. Two-sided *p* values calculated using Kruskal-Wallis test. Linear regression modeling showing *p* value and slope coefficient (*k*) when using B cell proportion and continuous age in the model. **b** Heatmap of 102 immune cell marker genes in 232 SCAN-B cases using Pearson correlation and ward.D linkage. Hierarchical clustering of cases (columns) and genes (rows) was performed using Pearson correlation as distance metric and ward.D linkage on mean-centered log2 transformed data with an offset of 0.1. **c** TIL percentage estimated from whole-slide hemotoxylin and eosin-stained sections versus stratified age groups in all cases (left), HRDetect-high, HRDetect-low, and PAM50 basal-like cases (right). Top axes indicate group sizes. Two-sided *p* values calculated using Kruskal-Wallis test. Linear regression modeling showing *p* value and slope coefficient (*k*) when using TIL percentage and continuous age in the model. For age group definitions, “[” equals ≥, “)” equals <, and “]” equals ≤ for the value specified next to it. In panels **a** and **c**, separate results of a sensitivity analysis for trend are reported in red due to very small sample numbers across the full stratified age range

For CIBERSORTx, which estimates proportions of six cell types (epithelial, macrophage, stroma, T and B cell, and endothelial), stratified age group testing (Kruskal-Wallis test) combined with linear regression modeling for trend (increase/decrease) showed that only the estimated B cell proportion per sample appeared to decrease with patient age in tested subgroups (albeit with a non-significant trend for HRDetect-low/intermediate cases) (Fig. 3a). To test whether these observations could be validated in situ, we performed CD20 immunohistochemistry and scored 200 of the 237 cases into four groups (0,1,2,3) based on staining patterns. With the exception of HRDetect-low/intermediate cases, the proportion of cases with the highest score (bin = 3) decreased with increasing patient age for all patients, HRDetect-high, and PAM50 basal-like patients (Additional file 1 M).

To further analyze mRNA expression of different immune cell marker genes, we clustered 102 genes related to 23 immune cell types (as previously defined in [25]) in the 232 SCAN-B cases with gene expression (Fig. 3b). This analysis demonstrated the heterogeneity of immune cell expression across stratified age groups, HRDetect defined groups, and the PAM50 subtypes. For B cell-associated genes in Fig. 3b, 83% were differentially expressed between age groups in the total cohort (*n* = 232 samples, Kruskal-Wallis FDR *p* < 0.05) supporting the CIBERSORTx and CD20 immunohistochemistry results.

No systematic differences in PD-L1 class (≥ 1%) or actual PD-L1 IHC scores (% stained cells) between age groups were observed (*n* = 218 evaluable cases, chi-square test *p* = 0.16, Kruskal-Wallis *p* = 0.20, respectively) (Additional file 1 N). Similar non-significant results were found in HRDetect-high, HRDetect-low/intermediate, and PAM50 basal-like cases separately (*p* > 0.05 for both tests in subgroups) (Additional file 1 N). We observed a decreasing trend of TILs assessed from whole-slide sections with age at diagnosis (*n* = 213, Kruskal-Wallis *p* = 0.001 and linear regression modeling *p* = 0.0001) (Fig. 3c). This trend remained significant when stratifying TIL evaluable cases into HRDetect-high (*n* = 126, *p* = 0.008 and *p* = 0.001, respectively) and PAM50 basal-like (*n* = 165, *p* = 0.051 and *p* = 0.001, respectively), but not in HRDetect-low/intermediate cases (Fig. 3c). To further analyze the difference in immune cell infiltration, we compared tumor mutational burden and expressed neoantigens modeled from somatic substitutions versus patient age. For tumor mutational burden, there was no consistent change in the number of substitutions and indels per Mb sequence between age groups that was matched with a significant trend (increase/decrease) in any of the tested patient groups (groups: all patients, HRDetect-high, HRDetect-low/intermediate, and PAM50 basal-like, all Kruskal-Wallis *p* > 0.05 and linear regression modeling *p* > 0.05, Additional file 1O). Similarly, no significant results were obtained for the number of expressed neoantigens per sample when modeled using the NeoPredPipe pipeline for the same patient groups (Additional file 1P).

### Difference in copy number alterations and driver mutations between young and elderly TNBC patients

To analyze the copy number landscape of TNBC with respect to age at diagnosis, we first calculated and compared (i) the fraction the of genome affected by copy number gain or loss, (ii) the fraction the of genome affected by LOH, (iii) weighted genome integrity index estimates [28], (iv) mutant-allele tumor heterogeneity scores [29], and (v) the individual components of the HRD score algorithm (LST, AI, HRD) [27]. With exception of the HRD score, there was no statistical difference for the different estimates in (i) all SCAN-B patients, (ii) HRDetect-high cases only, (iii) HRDetect-low cases only, or (iv) PAM50 basal-like cases when stratified by age groups (Kruskal-Wallis *p* > 0.05, linear regression modeling for trend *p* > 0.05, Additional file 1Q-T). For the HRD score components, decreasing trends with age were observed across all cases as well as the PAM50 basal-like group (Additional file 1 U). These observations can, however, be explained by that these patient groups are mixtures of HRD-high and HRD-low cases as previously shown [9].

**Fig. 4:**
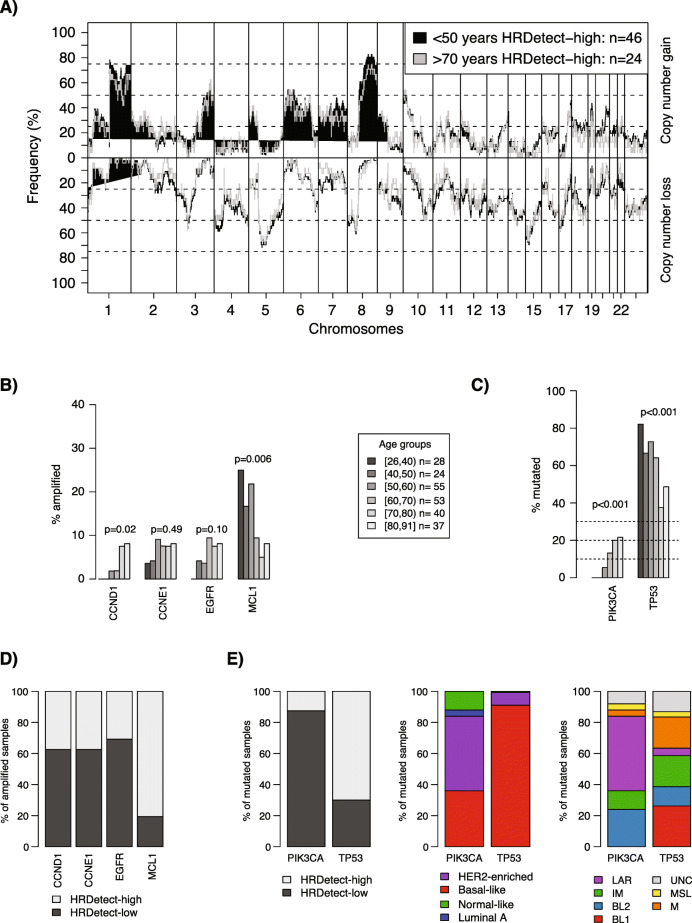
Copy number alterations versus age at diagnosis in TNBC. **a** Copy number landscape of HRDetect-high patients < 50 years at diagnosis versus > 70 years at diagnosis. **b** Difference in amplification frequency of *CCND1*, *CCNE1*, *EGFR*, and *MCL1* with age groups when analyzed in the total SCAN-B cohort. Two-sided *p* values calculated using chi-square test for trends in proportions. **c** Difference in mutation frequency of *PIK3CA* and *TP53* with age groups when analyzed in the total SCAN-B cohort. Two-sided *p* values calculated using chi-square test for trends in proportions. **d** Proportions of amplified cases for *CCND1*, *CCNE1*, *EGFR*, and *MCL1* according to HRDetect classification. **e** Proportions of mutated cases for *PIK3CA* and *TP53* according to HRDetect (left), PAM50 (center), and TNBCtype (right) classifications. For age group definitions, “[” equals ≥, “)” equals <, and “]” equals ≤ for the value specified next to it

Overall, age at diagnosis does not appear associated with the overall amount of copy number alterations in TNBC tumors. Illustrations of this are shown in Fig. 4a for HRDetect-high patients younger than 50 years (*n* = 46) versus HRDetect-high patients older than 70 years (*n* = 24) and in Additional file 3A-B, both showing similar copy number landscapes between the groups. Considering the previously described association of older age at diagnosis with the LAR gene expression subtype (Fig. 2, Table 1, and [6]), we compared the copy number landscape between older SCAN-B patients (> 70 years), LAR-classified SCAN-B patients, and reference populations of luminal B and basal-like-classified cases from Nik-Zainal et al. [3]. Both the > 70-year cohort and the LAR-classified patients showed more copy number similarities with basal-like tumors than luminal B-like cases (Additional file 3C).

Finally, we analyzed whether the frequency of reported amplifications and driver mutations in breast cancer [3] differ between the age groups in the total SCAN-B cohort. With higher age, trends of increasing amplification frequencies of *CCND1*, *CCNE1*, *EGFR*, and increasing mutation frequency of *PIK3CA* mutations were observed, while frequencies of *MCL1* amplifications and *TP53* mutations decreased (Fig. 4b, c). The alterations are however also significantly associated with HR status as well as PAM50 and TNBC subtypes (Fig. 4d, e). Unfortunately, the low number of amplified samples per age group precluded robust age stratified analysis within, e.g., HRD phenotypes.

### Mutational and rearrangement signatures versus patient age

**Fig. 5:**
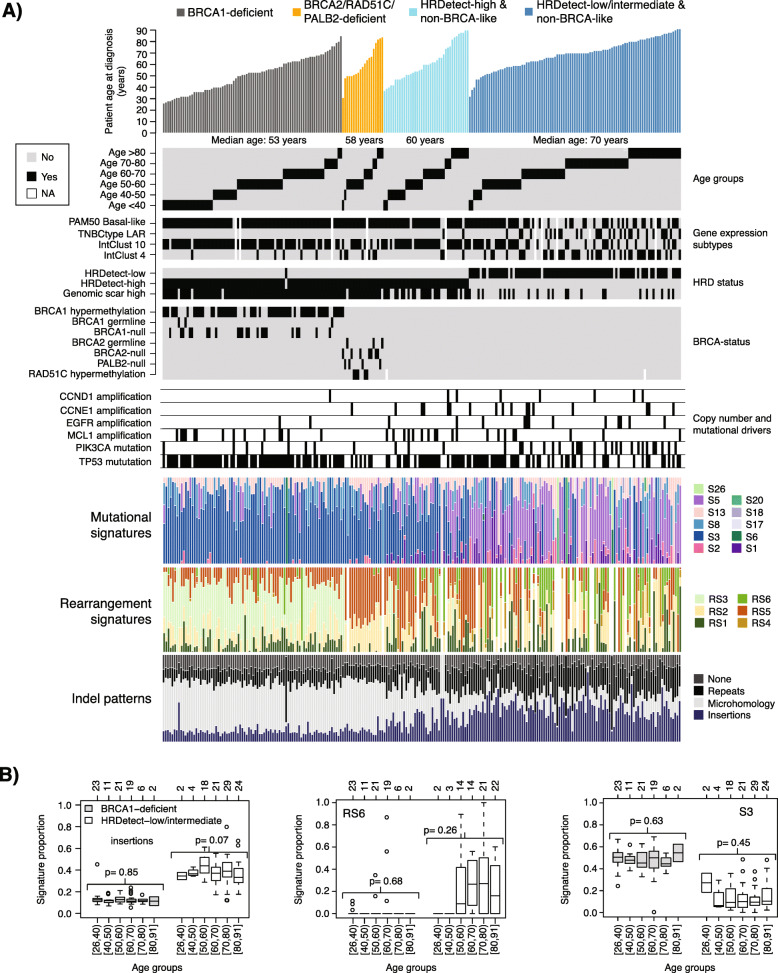
Composite view of molecular and genetic phenotypes versus age at diagnosis in TNBC. **a** Integrative view of gene expression subtypes (PAM50 basal-like, LAR, IntClust 4, 10), HRD classification, *BRCA1*, *BRCA2*, *PALB2*, *RAD51C*, *MCL1*, *CCND1*, *CCNE1*, *EGFR*, *PIK3CA*, and *TP53* alterations, mutational signatures (S1-S26), rearrangement signatures (RS1-RS6), patterns of insertion, and deletions versus age groups stratified by an underlying *BRCA1* deficiency, *BRCA2* deficiency, and HRDetect classification. BRCA1-null, BRCA2-null, and PALB2-null imply biallelic loss of the gene based on WGS. **b** Illustration of signature patterns in *BRCA1*-deficient tumors (light gray) and HRDetect-low/intermediate cases (white) stratified by age for proportion of insertions (left), rearrangement signature 6 (RS6, center), and mutational substitution signature 3 (S3, right, refitted by SigFit). RS6 is characterized by clustered rearrangements typically found in cases with driver amplifications, while S3 is associated with *BRCA1/2* deficiency [41]. Top axes indicate group sizes. Top axes indicate group sizes. Two-sided *p* values are calculated by Kruskal-Wallis test per group. For age group definitions, “[” equals ≥, “)” equals <, and “]” equals ≤ for the value specified next to it

To provide a composite view on clinical characteristics, gene expression subtypes, driver alterations, HRD status, and mutational and rearrangement signatures, we merged data for the 237 SCAN-B cases, subdivided by HRDetect and BRCA status and ordered by age at diagnosis (Fig. 5a). Interestingly, Fig. 5a illustrates that concordance between HRD algorithms appear to decrease in patients without *BRCA1/2* deficiency. For instance, across all 237 cases, agreement in HRD classification (high/low) between HRDetect and HRD score [27] was 83%, 94% in *BRCA1/2*-deficient cases, but only 74% in patients with no known BRCA1/2 deficiency. This difference is likely due to the tuning of the methods’ cut-offs and that the WGS-based HRDetect method captures additional levels of information compared to the copy number based HRD score. For patients with no known BRCA1/2-deficiency, HRD agreement varied between 65 and 81% across age groups (median = 75%, standard deviation 6%).

We next dissected the different mutational and rearrangement signatures shown in Fig. 5a in detail versus the patient age at diagnosis. For mutational signatures, we refitted substitutions from the original study using the SigFit algorithm [30] to account for the fact that the original signature fitting was made by the optimized HRDetect algorithm [9]. For the two mutational signatures, 1 and 5, proposed to be associated with age at diagnosis [42], a significant linear trend of increasing signature proportions with age at diagnosis was observed across all SCAN-B cases (linear regression *p* = 0.007 for signature 1 and *p* = 2e−6 for signature 5, Additional file 4A). To explore the underlying genetic phenotypes, we focused on *BRCA1*-deficient cases as prototypical examples of HR-deficient tumors and HRDetect-low/intermediate cases as their polar opposite. The need to stratify HRDetect-high tumors is clear from Fig. 5a, as *BRCA1*-deficient, *BRCA2*/*PALB2*/*RAD51C*-deficient, and cases without known HRD inactivation mechanism have clearly different genetic phenotypes concerning mutational and rearrangement signatures. For both tested  subgroups, we did not find differences in proportions of mutational signatures, rearrangement signatures, or indel types showing a significant trend across the stratified age groups (Kruskal-Wallis *p* > 0.01) (Fig. 5b and Additional file 4B-D). These non-significant results were further supported by linear regression modeling of proportions as a function of age (*p* > 0.01) (Additional file 4E-F). The significance of the underlying genetic phenotype(s) was further evident when performing the same analyses for the PAM50 basal-like, IntClust 10, and TNBCtype gene expression subtypes (mainly the BL1 subtype). Here, typical features of HRD [26], such as the proportion of deletions with junctional microhomology, mutational signature 3 proportions, and rearrangement signature 3 proportions, decreased with increasing patient age (Additional file 4G-I). However, these observations may be explained by the PAM50 basal-like, IntClust 10, and TNBCtype basal-like (BL1 and BL2) gene expression subtypes being mixtures of both HRD-high and HRD-low cases as shown in [9] and of the four genetic subtypes in Fig. 5a as shown in Additional file 5A. Similar to the total cohort (Fig. 2c), age at diagnosis did not capture transcriptional variation in the individual four genetic subtypes based on unsupervised principal component analysis (Additional file 5B). Moreover, the pattern of later onset of non-basal expression subtypes (Fig. 2d, e), and the LAR subtype (Fig. 2f) in the total cohort was reflected with sufficient numbers only in HRD-negative tumors when substratified (Additional file 5C). In contrast, in HRD-positive tumors the different TNBCtype subtypes did not appear to differ concerning their cumulative age distributions (Additional file 5D).

### Mutational and rearrangement signatures are not associated with age at diagnosis in molecular subtypes of breast cancer

To further address the question of differences in mutational and rearrangement signatures versus age at diagnosis in breast cancer in general, we utilized the Nik-Zainal et al. cohort comprising 560 WGS analyzed cases. The cohort includes 163 TNBCs and 320 ER-positive and HER2-negative (ER+/HER2−) tumors, and 255 of 560 cases had available PAM50 subtypes (55 basal-like, 10 HER2-enriched, 74 luminal A, 111 luminal B, and five normal-like cases). Based on group sizes, we focused on TNBC and ER+/HER2− tumors (stratified by HRDetect to account for underlying genetic HRD-high or low/intermediate phenotypes), as well as basal-like, luminal A and B subtype-classified cases.

When comparing the different signature proportions stratified by age groups (statistically evaluated by Kruskal-Wallis’s test) or using linear regression modeling of signature proportions as a function of age, only three significant observations were made across the patient subgroups after Bonferroni correction for multiple testing (summarized in Additional file 6 and shown in detail in Additional file 7A). Significant associations were all based on linear regression modeling, with corresponding age group stratifications not reaching significance after adjustment for multiple testing (Additional file 7A). Significant associations included an increase of insertions with patient age in PAM50 basal-like cases and increases of mutational signature 5 proportions with age in PAM50 basal-like and luminal A cases. For the two mutational signatures (signatures 1 and 5) previously associated with age at diagnosis [42], signature 5 appeared most consistent with increasing proportions with higher age across tested subgroups (Additional file 7B). Based on the copy number data available for the Nik-Zainal cohort, we could also conclude that the proportion of the tumor  genome affected by copy number gain or loss and the proportion of the genome affected by LOH did not differ for the different patient subsets listed in Table 2 in an age-related fashion. This held true irrespective of whether the analysis was performed using stratified age groups or linear regression modeling (Additional file 7C).

## Discussion

In the current study, we tested the hypothesis that TNBC in young and old breast cancer patients represents different molecular entities in the context of underlying genomic phenotypes, based on integration of multiple layers of genomic profiling by state-of-the-art sequencing techniques and in situ analysis of immune cell infiltration, CD20, and PD-L1 expression.

Above all, our study demonstrates the importance of analyzing age-related alterations in TNBC, as well as breast cancer in general, in the context of underlying genomic phenotypes. Considering the molecular heterogeneity of TNBC, this may appear self-evident, but has not previously been reported in a population-based cohort comprehensively profiled on as many levels as in the current study. In TNBC, the importance of a comprehensive approach is particularly highlighted by the genetic phenotypes caused by DNA repair deficiency, foremost *BRCA1/2*-deficiency that confers a characteristic HRD phenotype. Despite the size of the current study some limitations are apparent for specific subgroup analyses. This applies primarily to HRDetect-low/intermediate patients where the stratified group sizes for younger patients (< 50 years) are small and care needs to be taken in interpretation of their characteristics. To address this, sensitivity analyses are provided for features reported in Additional file 1 and Fig. 3a, c. In this study we show several examples for which trends appear significantly associated with age at diagnosis in general and even within/between molecular subtypes, but where substratified analyses reveal that the significance is restricted to a specific genomic phenotype. One such example is the Ki67 proliferation marker, for which levels have been reported to be lower in elderly patients [14, 19]. This is in agreement with our findings in the full SCAN-B cohort, but when stratified by HRD status, the decrease appears restricted to HRD-low/intermediate cases and not to HRD-high or *BRCA1/2*-deficient cases. Other examples include genome-wide mutational and rearrangement signatures, HRD score [27] components indicative of large-scale transitions and allelic imbalance, specific copy number alterations, and driver mutations. For these features, trends of increasing/decreasing proportions may be observed in the total cohort and even across different transcriptional subtypes (PAM50, IntClust10, and TNBCtype) if one does not acknowledge that these represent mixtures of genetic phenotypes.

For mutational and rearrangement signatures, tumor mutational burden, mutant-allele tumor heterogeneity scores [29], and the fraction of the genome affected by copy number alterations, patient age did not appear associated with consistent decreasing/increasing trends when accounting for the genetic background of the tumor. Thus, for these genome-wide characteristics, TNBC tumors appear shaped by specific mutational processes like DNA repair deficiency rather than age at diagnosis. A similar conclusion was reached in ER-positive disease based on analysis in the Nik-Zainal dataset. Moreover, despite the apparent lack of HR deficiency (as defined by [26, 27]) in older TNBC patients (including LAR subtype cases), these still resembled basal-like breast cancer more than, e.g., ER-positive luminal B disease with respect to genome-wide copy number alterations (Additional file 3), albeit with an increase of amplification drivers and *PIK3CA* mutations as previously noted [19]. Concerning the latter, the small number of affected cases unfortunately precludes a conclusive statement of whether such specific alterations are associated with age at diagnosis within a specific genetic or transcriptional TNBC subtype. The clearest exceptions for mutational signatures involved the two signatures previously associated with patient age at diagnosis (mutational signatures 1 and 5 often referred to as “clock” signatures) [42]. In both SCAN-B and the Nik-Zainal WGS data, signature 5 showed the most trend-like pattern of increasing mutational proportion with patient age. Interestingly, trends (estimated by regression coefficients) for both signatures appeared different across molecular subgroups, raising the question of whether the replicative “clock” has different rates in breast cancer subgroups, or alternatively is affected by conditions like replication stress.

While younger TNBC patients predominantly present with a typical basal-like transcriptional subtype (PAM50: basal-like, IntClust10: cluster 10), exceptions exist (Table 1). With increasing patient age, non-basal-like subtypes gain in proportion, illustrated by the LAR subtype originally proposed by Lehmann et al. [6] and the IntClust10 cluster 4 subtype proposed by Curtis et al. [32] (Fig. 2). Still, these non-basal-like subtypes only represent a subset of elderly patients as also noted by Ma et al. [19] (Table 1). The heterogeneity in gene expression subtype classification across age groups was further illustrated by both unsupervised and supervised analyses. The latter identified a weaker gradient-like signal of tumor proliferation consistent with Ki67 differences [14, 19] and findings by Ma et al. [19]. However, when age-related differentially expressed genes were used to cluster samples, the obtained clusters recapitulated the primary biological subdivision of HRD-positive and basal-like TNBCs from HRD-negative, less-proliferative, and non-basal-like tumors (Fig. 2g). Moreover, it is mainly within the HRD-low/intermediate genetic phenotype that a steroid/androgen-driven transcriptional subtype appears in a subset of elderly patients, which also coincided with differences in tumor microenvironment cell composition (e.g., higher stromal content, Fig. 2h). While TNBC-specific subtypes (e.g., [4, 6, 43, 44]) have been associated with tumor and microenvironment characteristics [5, 45], it remains unclear if these subtypes are all tumor intrinsic or whether the observed differences are more reflective of the tumor microenvironment. Taken together, our results demonstrate that age at diagnosis by itself does not define the transcriptional or genetic landscape of TNBC.

An observation that appeared more consistent also in different genetic backgrounds was a trend of decreasing TIL counts with patient age (Fig. 2 and Additional file 5E). The analysis is however limited by a low number of cases in certain age groups and thus requires additional validation especially for subgroups defined by, e.g., HRD status. This observation has been reported before in both TNBC and, e.g., lobular breast carcinoma [46, 47]. Interestingly, in our cohort, the TIL decrease was not mirrored by clear linear decreases of lymphocyte PD-L1 expression, tumor mutational burden, number of expressed neoantigens, or an immune-associated transcriptional signature in unsupervised and supervised gene expression analyses. Immune infiltration in breast cancers has been suggested to be T cell predominant [48] and with prognostic associations [49]. But the underlying causes of the infiltration and the heterogeneity between tumors remains largely unknown. Loi et al. identified increasing TIL levels with high histological grade in TNBC suggesting genomic instability as a possible trigger [47, 50]. However, in our data, older HRDetect-high patients with generally lower TIL levels had no signs of a less unstable genome compared to younger patients, and no trends of different in silico estimated T cell proportions in all patients or analyzed subgroups were observed. A weaker trend of decreasing B cell proportions, based on in silico deconvolution of RNAseq data and in situ immunohistochemistry analysis of CD20 expression, with increasing patient age was observed. This observation warrants confirmation in larger materials, especially for subgroups based on HRD status due to small sample sizes. Moreover, how this B cell trend is associated with the TIL decrease in tumor tissue remains to be further examined. Notably, age-related immunosenescence characterized by a decrease in cell-mediated immune function as well as reduced humoral immune responses, including reduction of mature B cells, is well-established in healthy human subjects [51].

The clinical significance of patient age in TNBC remains controversial [10–14]. In our cohort, long-term outcome after adjuvant chemotherapy was not associated with age at diagnosis. It needs to be acknowledged that elderly patients in our cohort often did not receive adjuvant chemotherapy due to national guidelines and regional practice at the time, representing a potential source of bias. Based on that the genetic features of, e.g., BRCA deficiency and HRD (which both appear prognostic after adjuvant chemotherapy treatment [9, 25]) do not change with age, it may be argued that withholding treatment based on age alone could for some patients at least be reconsidered. Moreover, for a subset of HRD-negative elderly patients with mainly the LAR subtype alternative therapies like anti-androgens may be considered [52, 53]. In contrast, while young age at diagnosis is strongly associated with HRD (Table 1), it appears particularly important to identify young/middle aged patients without an HRD phenotype, as these may derive greater benefit from other types of treatment than conventional chemotherapy. In this context, our observation of a greater discrepancy between HRD methods for particularly non-*BRCA1/2*-deficient patients becomes a relevant issue to consider for the choice of HRD classification method in a diagnostic setting (Fig. 5a).

From a genetic perspective, TNBC represents at minimum two types of disease, a DNA repair-deficient disease with genetic scars strongly associated with *BRCA1/2* deficiency and a basal-like phenotype, and a second entity in which there is a larger heterogeneity concerning transcriptional subtypes [9]. For genetic subtypes (illustrated by the decline of *BRCA1* deficiency), a switch is apparent at 60–70 years of age coinciding with the median age of diagnosis of ER+ disease. Considering previous studies, it has been hypothesized that molecular differences between younger and older women may be more related to the differences in the tissue microenvironment of a pre-menopausal and post-menopausal patient rather than intratumoral biological differences [15]. A limitation is however that we are currently unable to assess at which time and in which cell type a tumor forms, representing an intriguing but challenging research area. Moreover, strong associations with specific microenvironmental, transcriptomic, and genetic phenotype in addition to patient age for certain subgroups, e.g., the LAR subtype, make definitive conclusions regarding causality challenging. An additional caveat with a population-based cohort such as ours is the influence of screening, particularly if biased towards specific high-risk groups such as families with known hereditary risk factors for breast cancer. In most cases however, exceptions exist wherein features of old patients’ tumors present in a young patient and vice versa, arguing in favor of the primacy of tumor-intrinsic rather than patient-level characteristics in tumor evolution.

## Conclusions

Our study demonstrates that in TNBC, age at diagnosis alone does not appear to provide an additional layer of biologic complexity above that of proposed genetic and transcriptional phenotypes. It may thus be argued that decisions regarding treatment regimens should be less influenced by age and more driven by actual tumor biology that needs to be carefully assessed through modern molecular diagnostics.

## Supplementary Information

**Additional file 1.** A PDF file with supplementary Figure 1 showing different associations of molecular and clinicopathological variables with patient age at diagnosis.

**Additional file 2.** A Microsoft Excel file (.xlsx) including supplementary Table 1 showing PANTHER gene ontology results for supervised SAM analysis.

**Additional file 3.** A PDF file with supplementary Figure 2 showing copy number alterations in subgroups of TNBC.

**Additional file 4.** A PDF file with supplementary Figure 3 showing mutational and rearrangement signatures in SCAN-B TNBCs.

**Additional file 5.** A PDF file with supplementary Figure 4 showing molecular characteristics of genetic subtypes of SCAN-B TNBCs related to the main Fig. 5.

**Additional file 6.** A PDF file including supplementary Table 2 showing patterns of mutational and rearrangement signatures in the general Nik-Zainal et al. breast cancer cohort.

**Additional file 7.** A PDF file with supplementary Figure 5 showing mutational and rearrangement signatures in 560 WGS analyzed breast cancers.

## Data Availability

Genomic data used in the current study is available in open repositories as described in the original studies.

## Abbreviations

DRFI: Distant relapse-free interval
FDR: False discovery rate
HRD: Homologous recombination deficiency
IDFS: Invasive disease-free survival
IntClust10: Integrative cluster subtype
LAR: Luminal androgen receptor subtype
OS: Overall survival
SCAN-B: Sweden Cancerome Analysis Network – Breast study
TIL: Tumor-infiltrating lymphocyte
TNBC: Triple-negative breast cancer
