# Correction to: Molecular analyses of triple-negative breast cancer in the young and elderly

**DOI:** 10.1186/s13058-021-01405-y

**Published:** 2021-02-24

**Authors:** Mattias Aine, Ceren Boyaci, Johan Hartman, Jari Häkkinen, Shamik Mitra, Ana Bosch Campos, Emma Nimeus, Anna Ehinger, Johan Vallon-Christersson, Åke Borg, Johan Staaf

**Affiliations:** 1 Division of Oncology, Department of Clinical Sciences Lund, Lund University, Medicon Village, SE-22381 Lund, Sweden; 2 Department of Clinical Pathology and Cytology, Karolinska University Laboratory, Stockholm, Sweden; 3 Department of Oncology and Pathology, Karolinska Institute, Stockholm, Sweden; 4 Division of Clinical Genetics, Department of Laboratory Medicine, Lund University, Lund, Sweden; 5 Division of Surgery, Department of Clinical Sciences, Lund University, Lund, Sweden; 6 Department of Genetics and Pathology, Laboratory Medicine, Region Skåne, Lund, Sweden

**Correction to: Breast Cancer Res (2021) 23:20**

**https://doi.org/10.1186/s13058-021-01392-0**

After publication of the original article [1], the authors identified an error in Fig. 4A. The correct figure is given below.

**Fig. 4:**
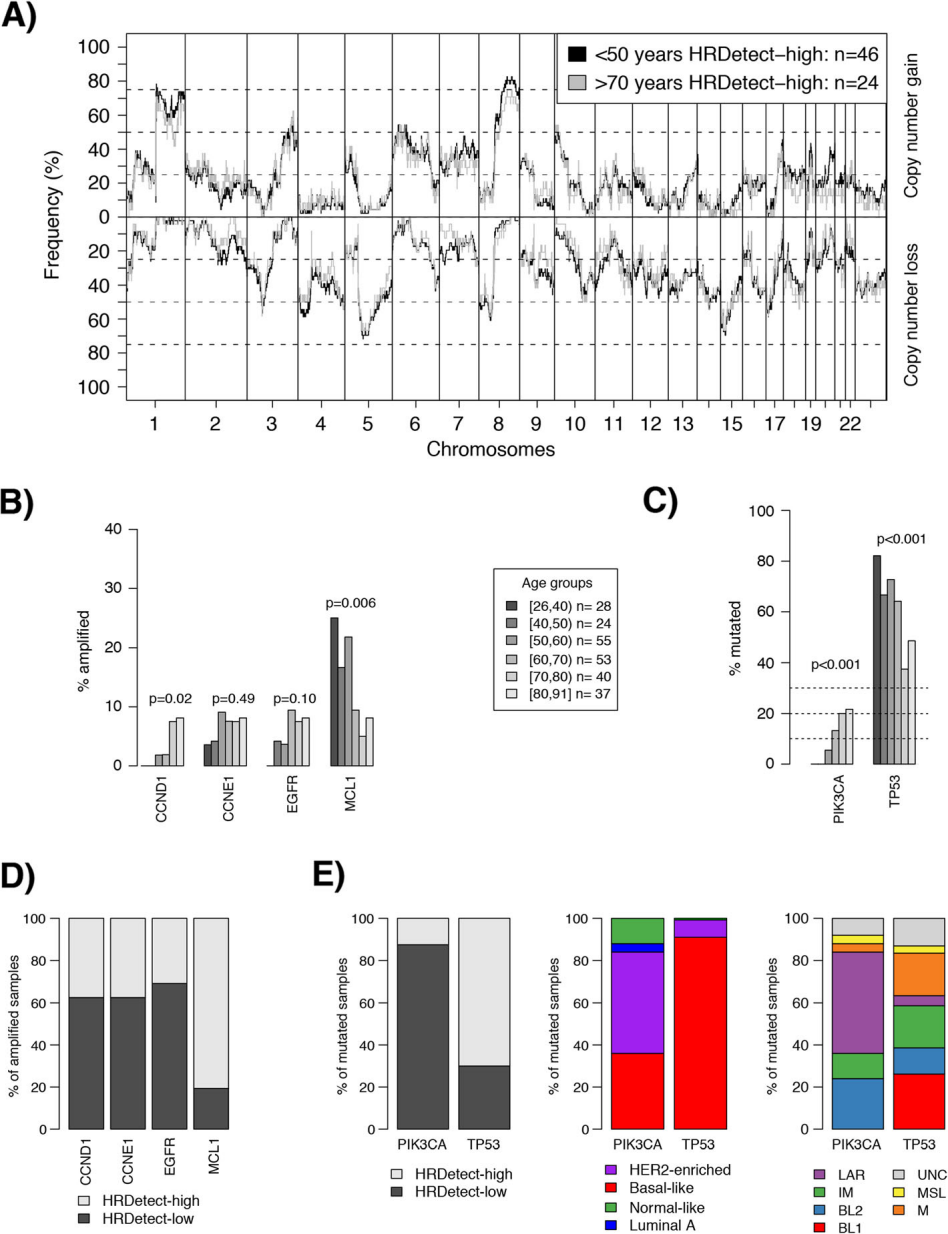
Copy number alterations versus age at diagnosis in TNBC. **a** Copy number landscape of HRDetect-high patients < 50 years at diagnosis versus > 70 years at diagnosis. **b** Difference in amplification frequency of CCND1, CCNE1, EGFR, and MCL1 with age groups when analyzed in the total SCAN-B cohort. Two-sided *p* values calculated using chi-square test for trends in proportions. **c** Difference in mutation frequency of PIK3CA and TP53 with age groups when analyzed in the total SCAN-B cohort. Two-sided p values calculated using chi-square test for trends in proportions. **d** Proportions of amplified cases for CCND1, CCNE1, EGFR, and MCL1 according to HRDetect classification. **e** Proportions of mutated cases for PIK3CA and TP53 according to HRDetect (left), PAM50 (center), and TNBCtype (right) classifications. For age group definitions, “[” equals ≥, “)” equals <, and “]” equals ≤ for the value specified next to it

The original article has been corrected.
