# Supplementary material for: Molecular analyses of triple-negative breast cancer in the young and elderly
Source: Breast Cancer Res. 2021 Feb 10;23:20. doi: 10.1186/s13058-021-01392-0 (PMC7874480; doi:10.1186/s13058-021-01392-0)
Supplement: Supplementary file 1 — Additional file 1. A PDF file with supplementary Figure 1 showing different associations of molecular and clinicopathological variables with patient age at diagnosis. [file 13058_2021_1392_MOESM1_ESM.pdf]

Supplementary Figure 1. Associations of molecular and pathology variables with patient age at diagnosis in SCAN-B TNBC patients. (**A**) ESR1 mRNA expression (FPKM) versus defined age groups for all cases and cases divided by HRD (HRDetect) status. (**B**) Ki67% as estimated by immunohistochemistry versus patient age at diagnosis for all SCAN-B patients. (**C**) MKI67 (Ki67) gene expression (FPKM) versus patient age at diagnosis for all SCAN-B patients. (**D**) MKI67 gene expression (FPKM) for SCAN-B patients stratified by HRDetect status. Left, HRDetect-high classified patients. Right, HRDetect-low/intermediate classified patients. The decrease in age in the latter group is significant (Kruskal-Wallis p<0.05, while no trend was observed in the former. (**E**) Scatter plot of MKI67 gene expression (FPKM) of tumors versus individual patient age for HRDetect-low/intermediate classified SCAN-B patients. Red line corresponds to linear regression fit. (**F**) Boxplot of tumor cell content (%) determined by the ASCAT algorithm, as outlined in Staaf et al. Nat Med 2019, versus age groups. (**G**) Age at diagnosis for patients with different mechanisms of *BRCA1* gene inactivation. Cases with mutations are stratified based on whether patients were clinically screened or not. (**H**) AR mRNA expression (FPKM) versus age stratified groups in all patients, HRDetect-high patients, HRDetect-low/intermediate patients, HRDetect-low/intermediate & non-LAR patients, and LAR patients only. (**I**) Steroid module (Fredlund et al. BCR 2012) gene expression scores versus stratified age groups in all patients, HRDetect-high patients, HRDetect-low/intermediate patients, and HRDetect-low/intermediate & non-LAR patients. (**J**) Distribution of age groups versus hierarchical clusters based on unsupervised clustering of 1179 differentially expressed genes as shown in main figure 2G. (**K**) Distribution of PAM50 molecular subtypes for the hierarchical clusters in main figure 2G. (**L**) Distribution of TNBCtype subtypes for the hierarchical clusters in main figure 2G. (**M**) 200 of 237 cases were evaluated for CD20 antibody staining on TMA slides. Pathology based scoring divided cases into four groups (0,1,2,3) with increasing stained cells. Scatter plot shows the proportion of patients in different patient subsets (colored lines) with the highest score (group=3) versus age stratified bins. A decrease in proportion is seen for increasing age for groups except for the HRDetect-low/intermediate group. (**N**) PD-L1 IHC scores versus stratified age groups, HRDetect-high, HRDetect-low/intermediate and PAM50 basal-like cases. (**O**) Tumor mutational burden (TMB) calculated as number of substitutions and indels per MBp sequence versus stratified age groups for all patients, HRDetect-high, HRDetect-low/intermediate, and PAM50 basal-like cases. In no instance did supportive linear regression modelling for trends show a consistent increase / decrease with age as indicated by regression slopes and p-values. (**P**) Total number of expressed neoantigens from NeoPredPipe, processed as described in Glodzik et al. NatCom 2020, versus stratified age groups for all patients, HRDetect-high, HRDetect-low/intermediate, and PAM50 basal-like cases. In no instance did supportive linear regression modelling for trends show a consistent increase / decrease with age as indicated by regression slopes and p-values. Neoantigen prediction involves RNAseq data, thus the number of cases is lower than for TMB as 232 of 237 cases had RNAseq data. (**Q**) Proportion of the genome altered by copy number gain or loss versus age groups for subsets of patients. (**R**) Proportion of the genome altered by loss of heterozygosity versus age groups for subsets of patients. (**S**) wGII scores versus age groups for subsets of patients. (**T**) MATH scores versus age groups for subsets of patients. (**U**) Scores for individual genomic scars components (LST, AI, HRD) for all SCAN-B patients and PAM50 basal like classified cases stratified by age groups. P-values (two-sided) are calculated using Kruskal-Wallis test.

For the age group definitions these are indicated as "[" meaning equal or greater than, ")" meaning smaller than, or "]" meaning smaller or equal than the value specified next to it.

P-values and linear regression values marked in red applies only to the subset of patients/groups marked by red inclusion bars.

A)

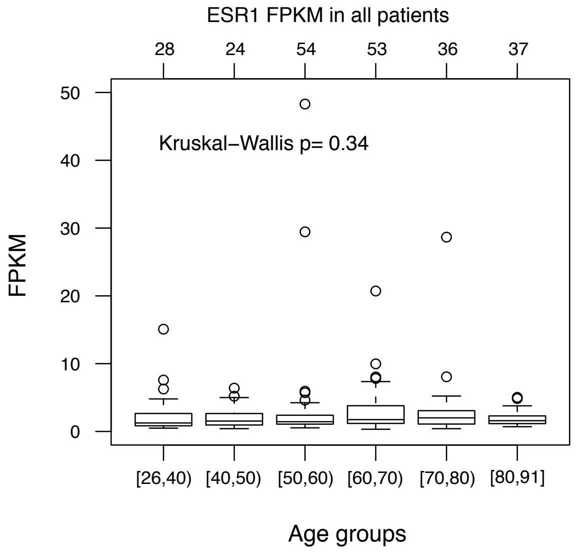

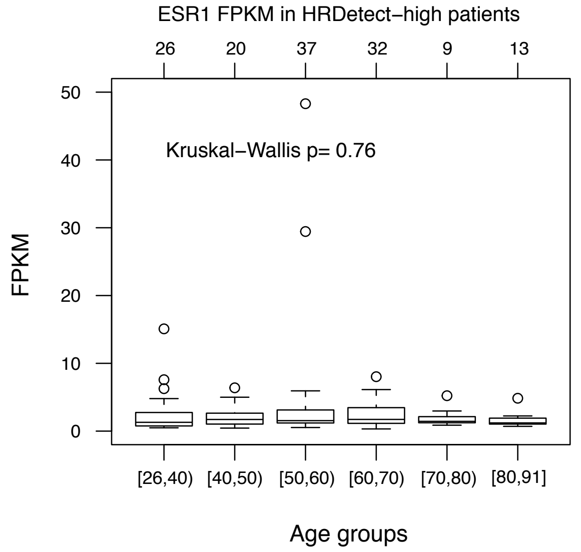

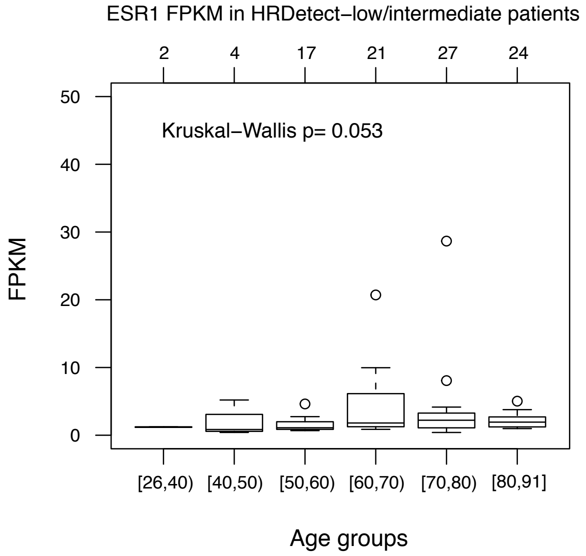

B)

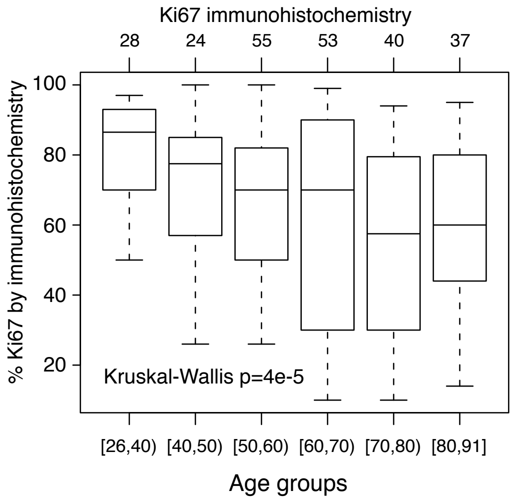
Ki67 immunohistochemistry
28 24 55 53 40 37
% Ki67 by immunohistochemistry
100
80
60
40
20
Kruskal-Wallis p=4e-5
[26,40) [40,50) [50,60) [60,70) [70,80) [80,91]
Age groups

C)

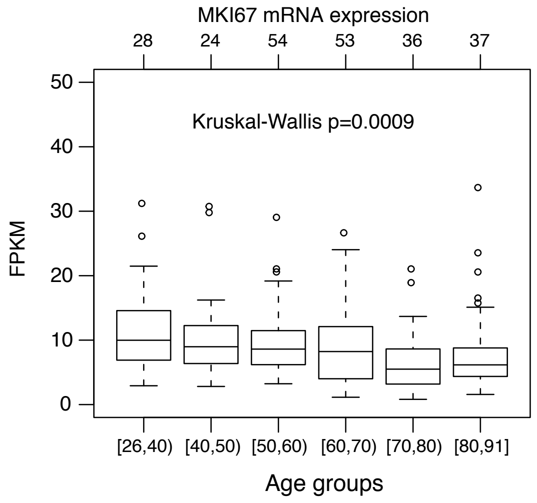
MKI67 mRNA expression
28 24 54 53 36 37
50
40
30
20
10
0
FPKM
Kruskal-Wallis p=0.0009
[26,40) [40,50) [50,60) [60,70) [70,80) [80,91]
Age groups

D)

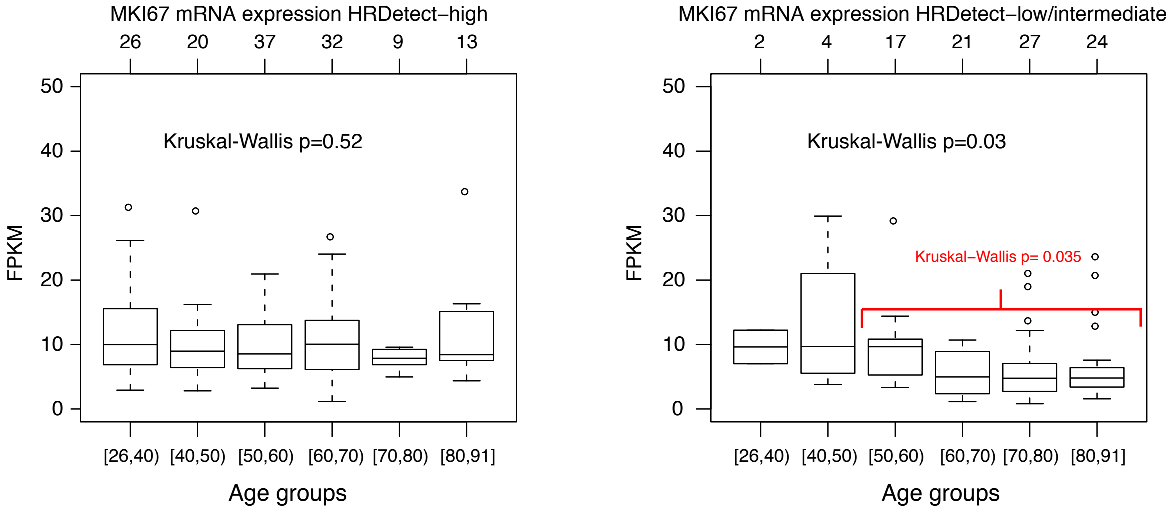
MKI67 mRNA expression HRDetect–high
26 20 37 32 9 13
50
40
30
20
10
0
FPKM
Kruskal-Wallis p=0.52
[26,40) [40,50) [50,60) [60,70) [70,80) [80,91]
Age groups
MKI67 mRNA expression HRDetect–low/intermediate
2 4 17 21 27 24
50
40
30
20
10
0
FPKM
Kruskal-Wallis p=0.03
Kruskal–Wallis p= 0.035
[26,40) [40,50) [50,60) [60,70) [70,80) [80,91]
Age groups

E)

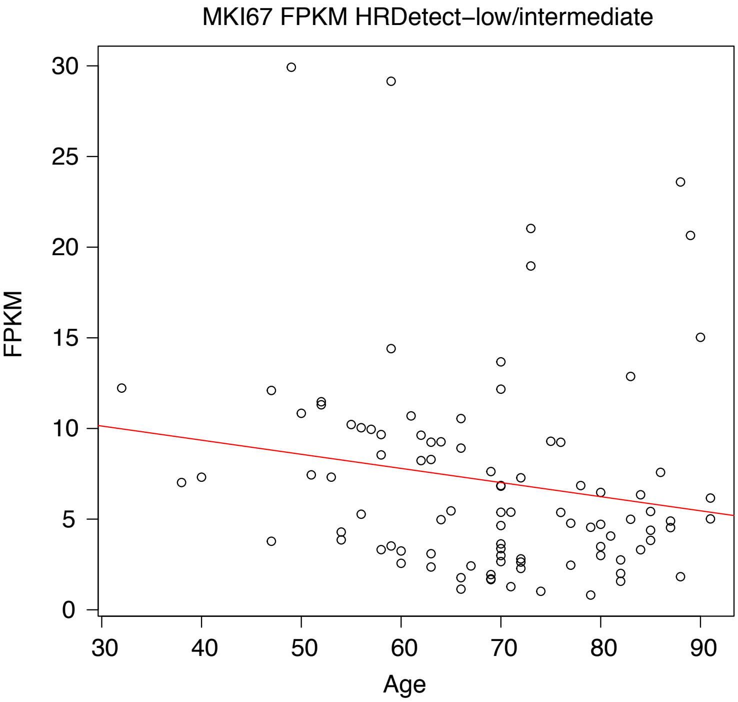

F)

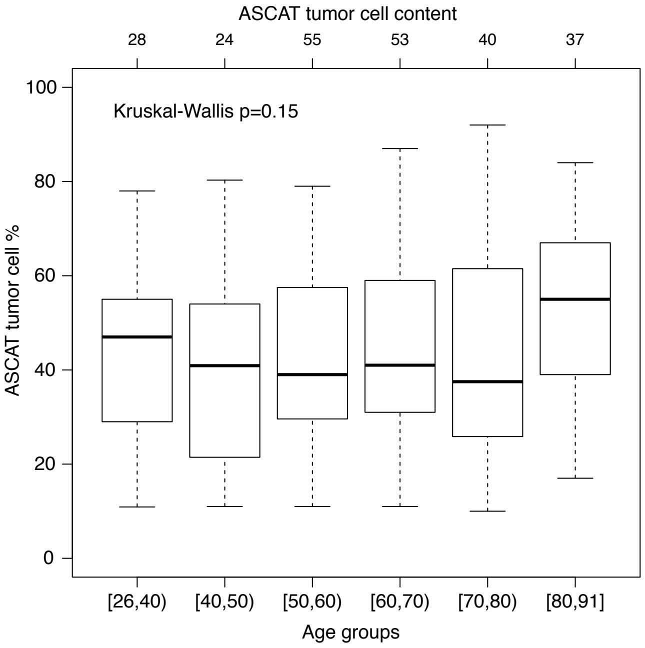

G)

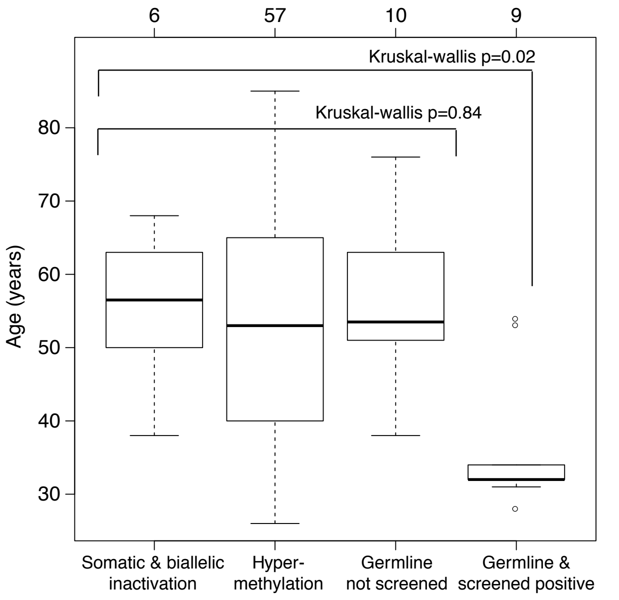

BRCA1-deficiency according to Staaf et al. Nature Medicine 2019

H)

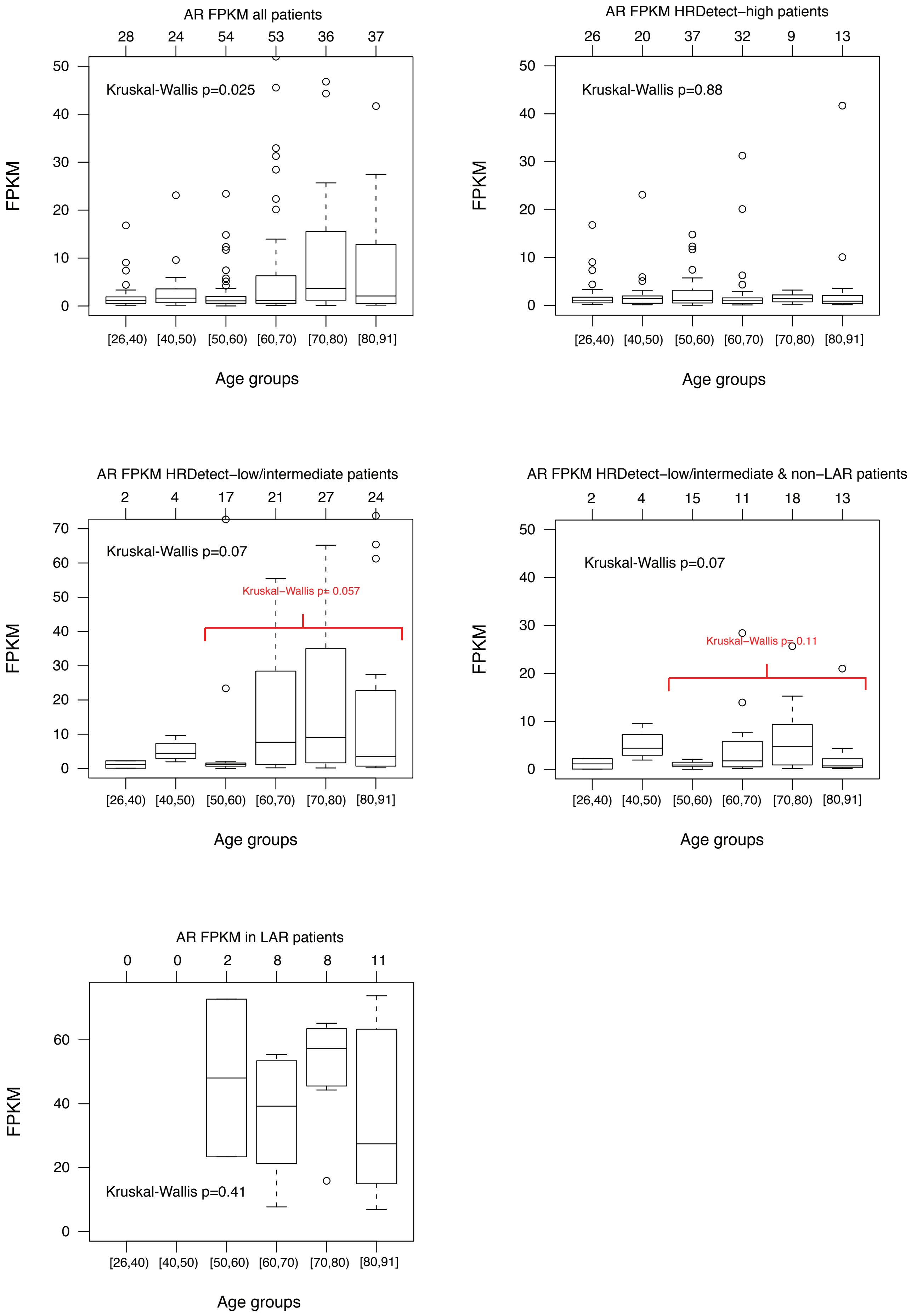
AR FPKM all patients
28 24 54 53 36 37
Kruskal-Wallis p=0.025
FPKM
[26,40) [40,50) [50,60) [60,70) [70,80) [80,91]
Age groups
AR FPKM HRDetect−high patients
26 20 37 32 9 13
Kruskal-Wallis p=0.88
FPKM
[26,40) [40,50) [50,60) [60,70) [70,80) [80,91]
Age groups
AR FPKM HRDetect−low/intermediate patients
2 4 17 21 27 24
Kruskal-Wallis p=0.07
Kruskal−Wallis p= 0.057
FPKM
[26,40) [40,50) [50,60) [60,70) [70,80) [80,91]
Age groups
AR FPKM HRDetect−low/intermediate & non−LAR patients
2 4 15 11 18 13
Kruskal-Wallis p=0.07
Kruskal−Wallis p= 0.11
FPKM
[26,40) [40,50) [50,60) [60,70) [70,80) [80,91]
Age groups
AR FPKM in LAR patients
0 0 2 8 8 11
Kruskal-Wallis p=0.41
FPKM
[26,40) [40,50) [50,60) [60,70) [70,80) [80,91]
Age groups

I)

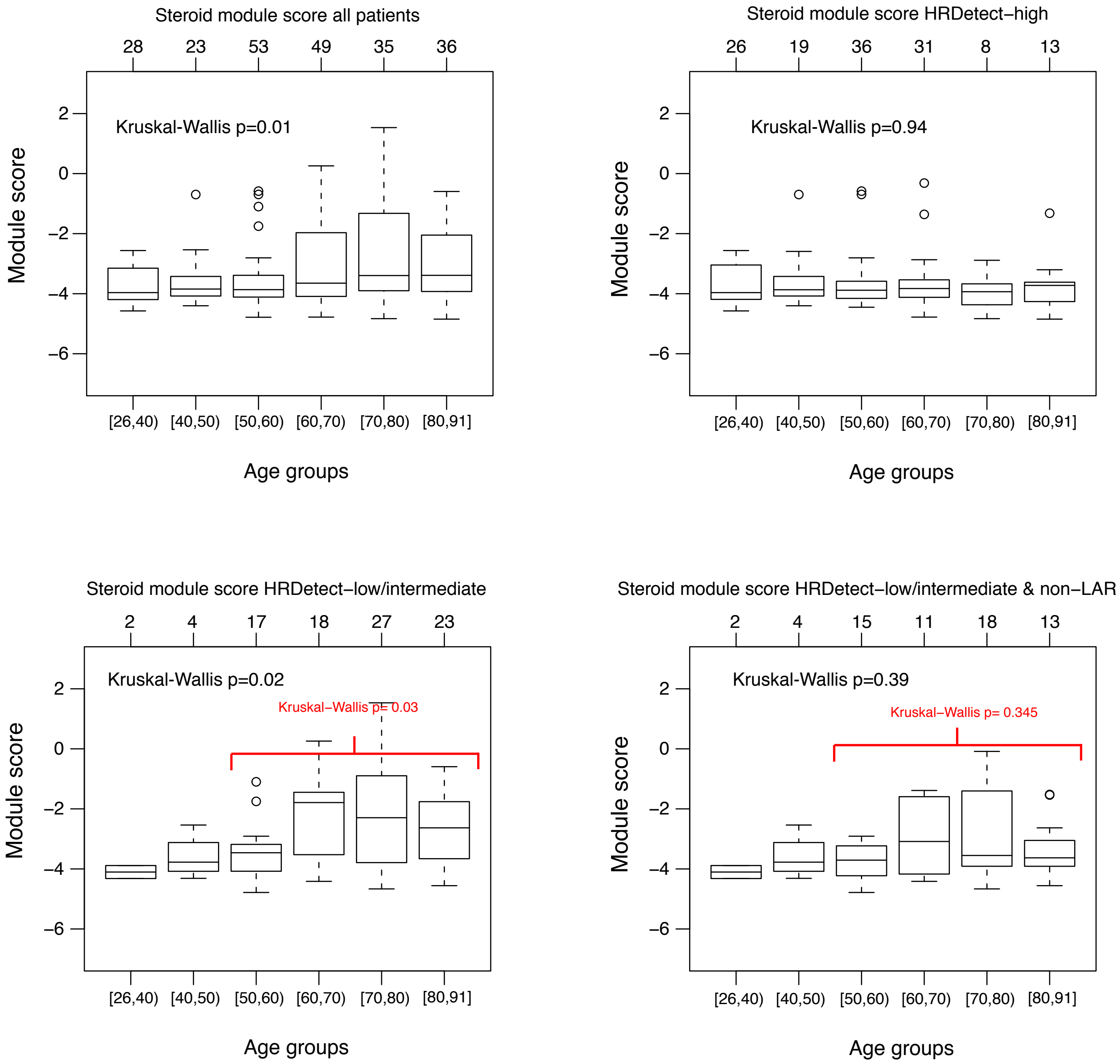
Steroid module score all patients
28 23 53 49 35 36
Kruskal-Wallis p=0.01
Module score
Age groups
[26,40) [40,50) [50,60) [60,70) [70,80) [80,91]
Steroid module score HRDetect−high
26 19 36 31 8 13
Kruskal-Wallis p=0.94
Steroid module score HRDetect−low/intermediate
2 4 17 18 27 23
Kruskal-Wallis p=0.02
Kruskal−Wallis p= 0.03
Steroid module score HRDetect−low/intermediate & non−LAR
2 4 15 11 18 13
Kruskal-Wallis p=0.39
Kruskal−Wallis p= 0.345

J)

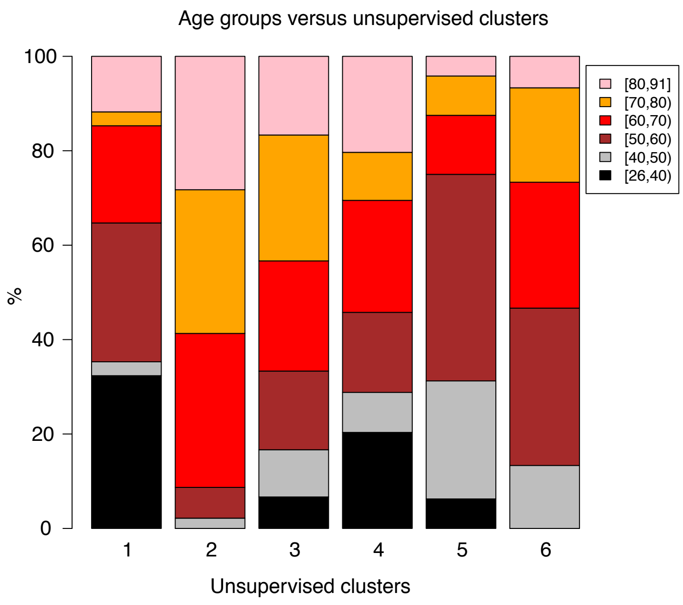
Age groups versus unsupervised clusters
100
80
60
40
20
0
%
1
2
3
4
5
6
Unsupervised clusters
[80,91]
[70,80)
[60,70)
[50,60)
[40,50)
[26,40)

K)

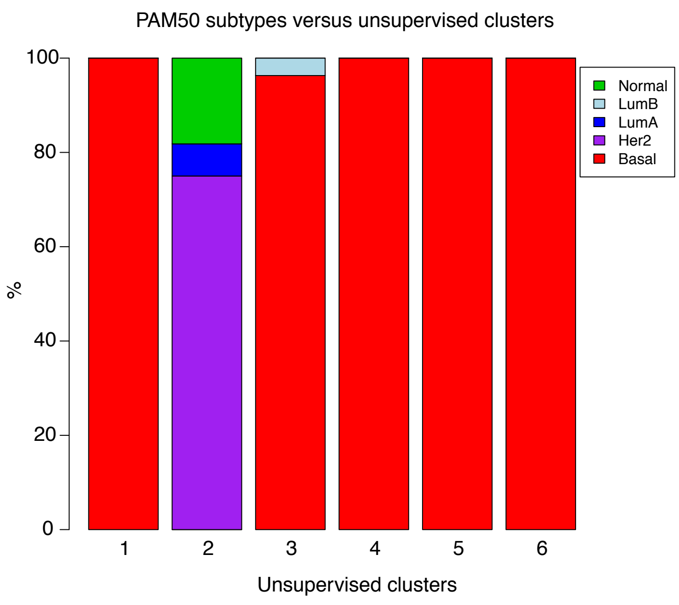
PAM50 subtypes versus unsupervised clusters
100
80
60
40
20
0
%
1
2
3
4
5
6
Unsupervised clusters
Normal
LumB
LumA
Her2
Basal

L)

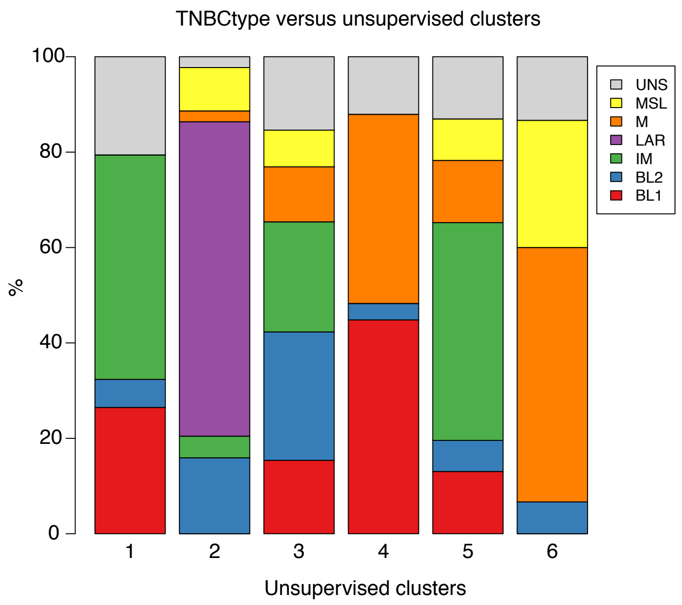
TNBCtype versus unsupervised clusters
100
80
60
40
20
0
%
1
2
3
4
5
6
Unsupervised clusters
UNS
MSL
M
LAR
IM
BL2
BL1

M)

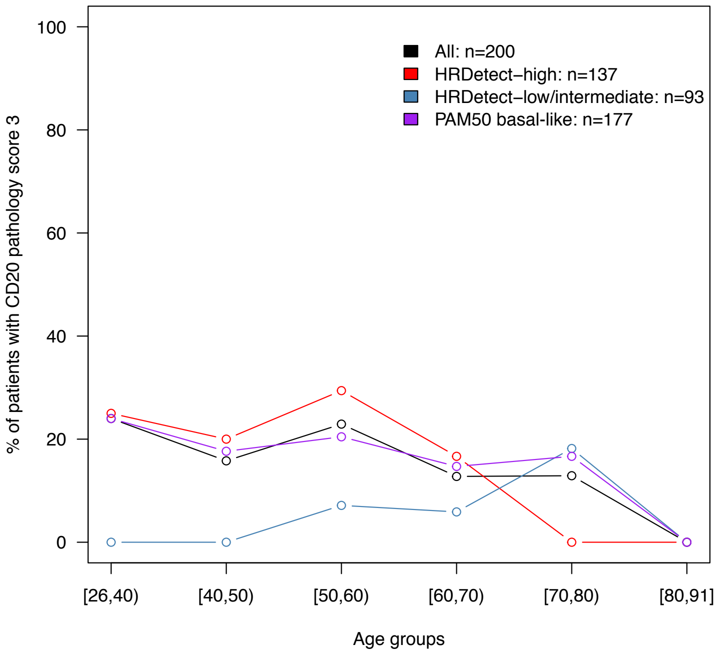
100
80
60
40
20
0
% of patients with CD20 pathology score 3
All: n=200
HRDetect−high: n=137
HRDetect−low/intermediate: n=93
PAM50 basal-like: n=177
[26,40)
[40,50)
[50,60)
[60,70)
[70,80)
[80,91]
Age groups

N)

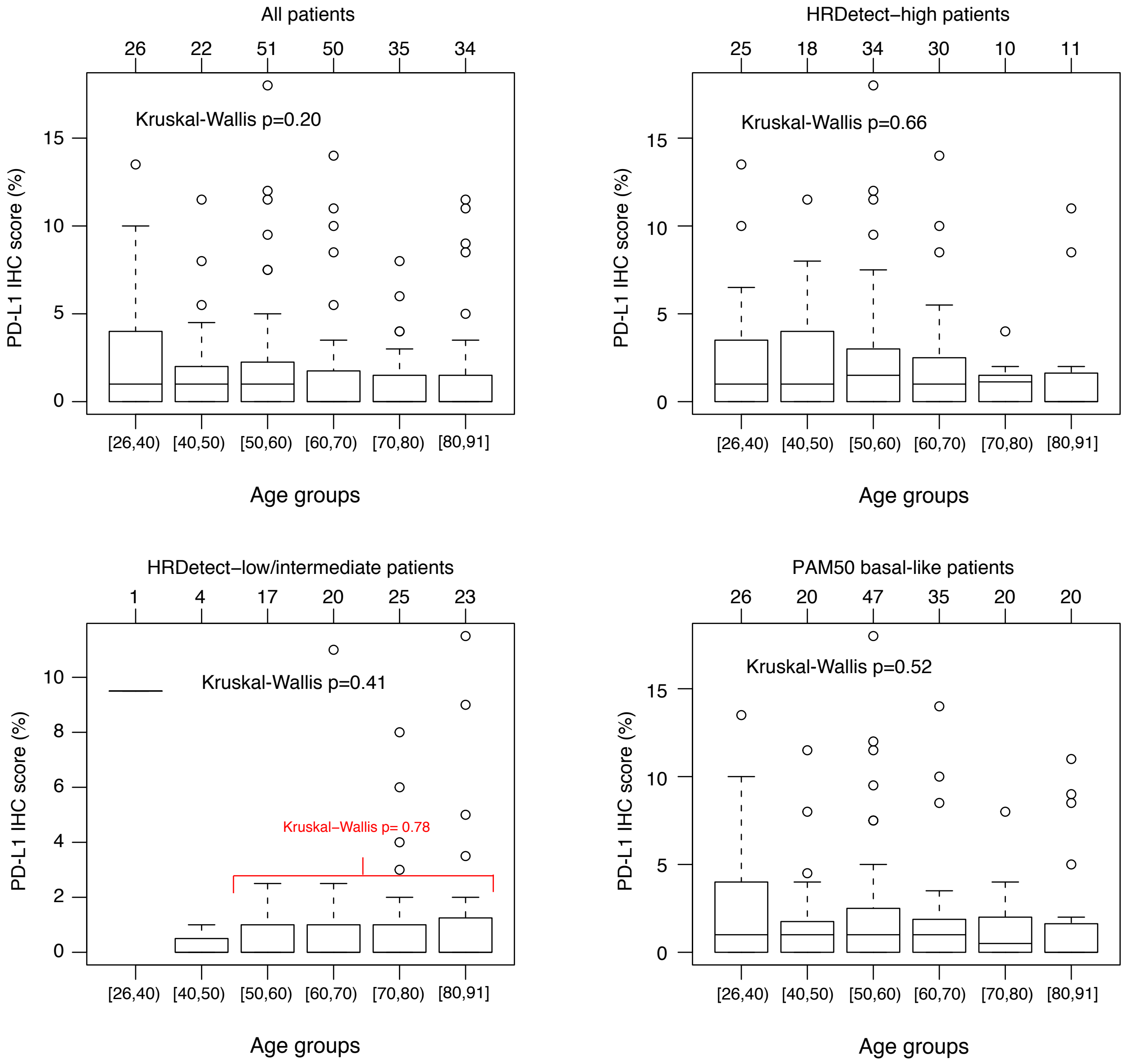
All patients
26
22
51
50
35
34
Kruskal-Wallis p=0.20
PD-L1 IHC score (%)
[26,40)
[40,50)
[50,60)
[60,70)
[70,80)
[80,91]
Age groups
HRDetect−high patients
25
18
34
30
10
11
Kruskal-Wallis p=0.66
HRDetect−low/intermediate patients
1
4
17
20
25
23
Kruskal-Wallis p=0.41
Kruskal−Wallis p= 0.78
PAM50 basal-like patients
26
20
47
35
20
20
Kruskal-Wallis p=0.52

O)

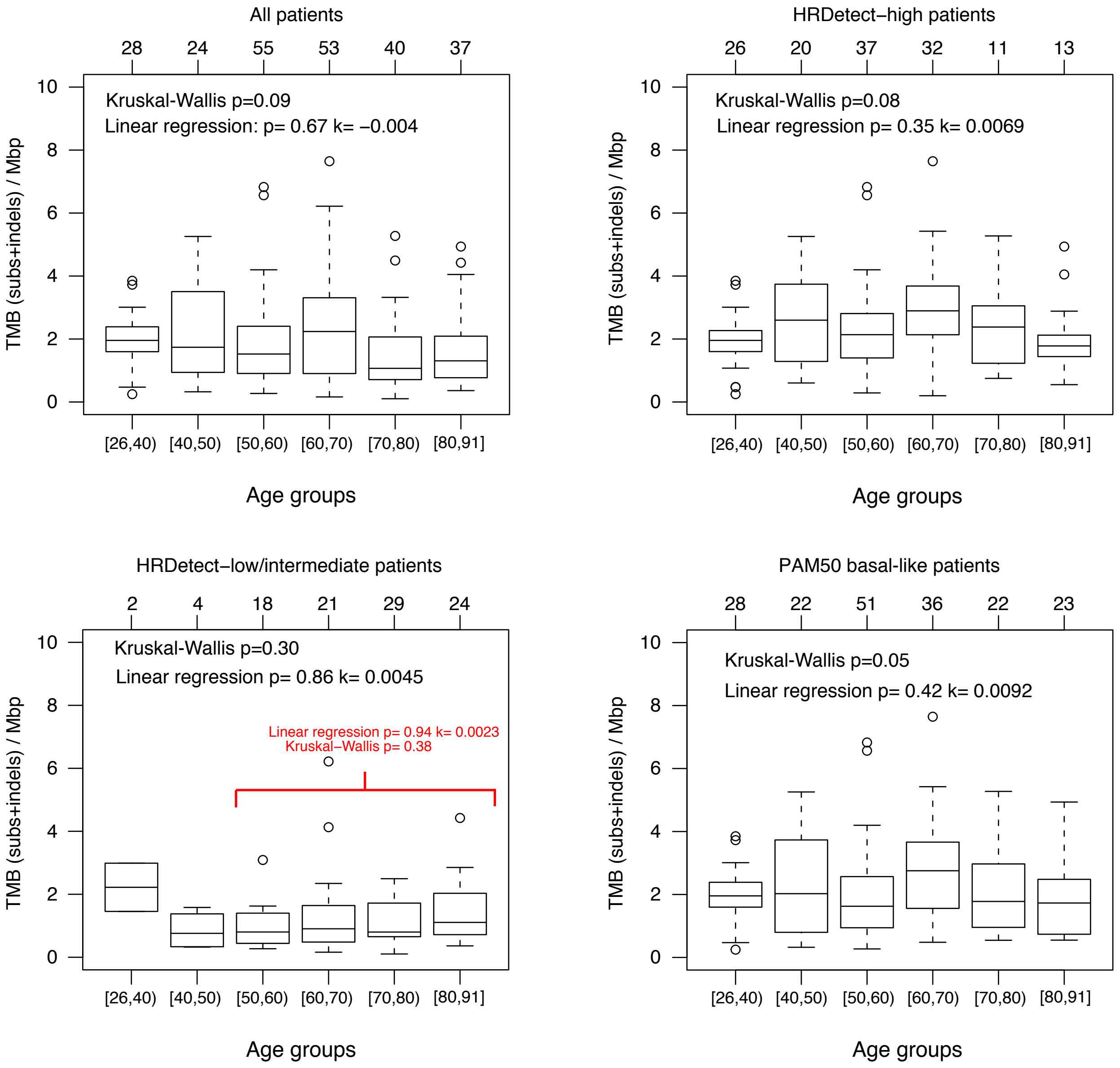
All patients
28 24 55 53 40 37
Kruskal-Wallis p=0.09
Linear regression: p= 0.67 k= −0.004
TMB (subs+indels) / Mbp
0 2 4 6 8 10
[26,40) [40,50) [50,60) [60,70) [70,80) [80,91]
Age groups
HRDetect–high patients
26 20 37 32 11 13
Kruskal-Wallis p=0.08
Linear regression p= 0.35 k= 0.0069
TMB (subs+indels) / Mbp
0 2 4 6 8 10
[26,40) [40,50) [50,60) [60,70) [70,80) [80,91]
Age groups
HRDetect–low/intermediate patients
2 4 18 21 29 24
Kruskal-Wallis p=0.30
Linear regression p= 0.86 k= 0.0045
Linear regression p= 0.94 k= 0.0023
Kruskal–Wallis p= 0.38
TMB (subs+indels) / Mbp
0 2 4 6 8 10
[26,40) [40,50) [50,60) [60,70) [70,80) [80,91]
Age groups
PAM50 basal-like patients
28 22 51 36 22 23
Kruskal-Wallis p=0.05
Linear regression p= 0.42 k= 0.0092
TMB (subs+indels) / Mbp
0 2 4 6 8 10
[26,40) [40,50) [50,60) [60,70) [70,80) [80,91]
Age groups

P)

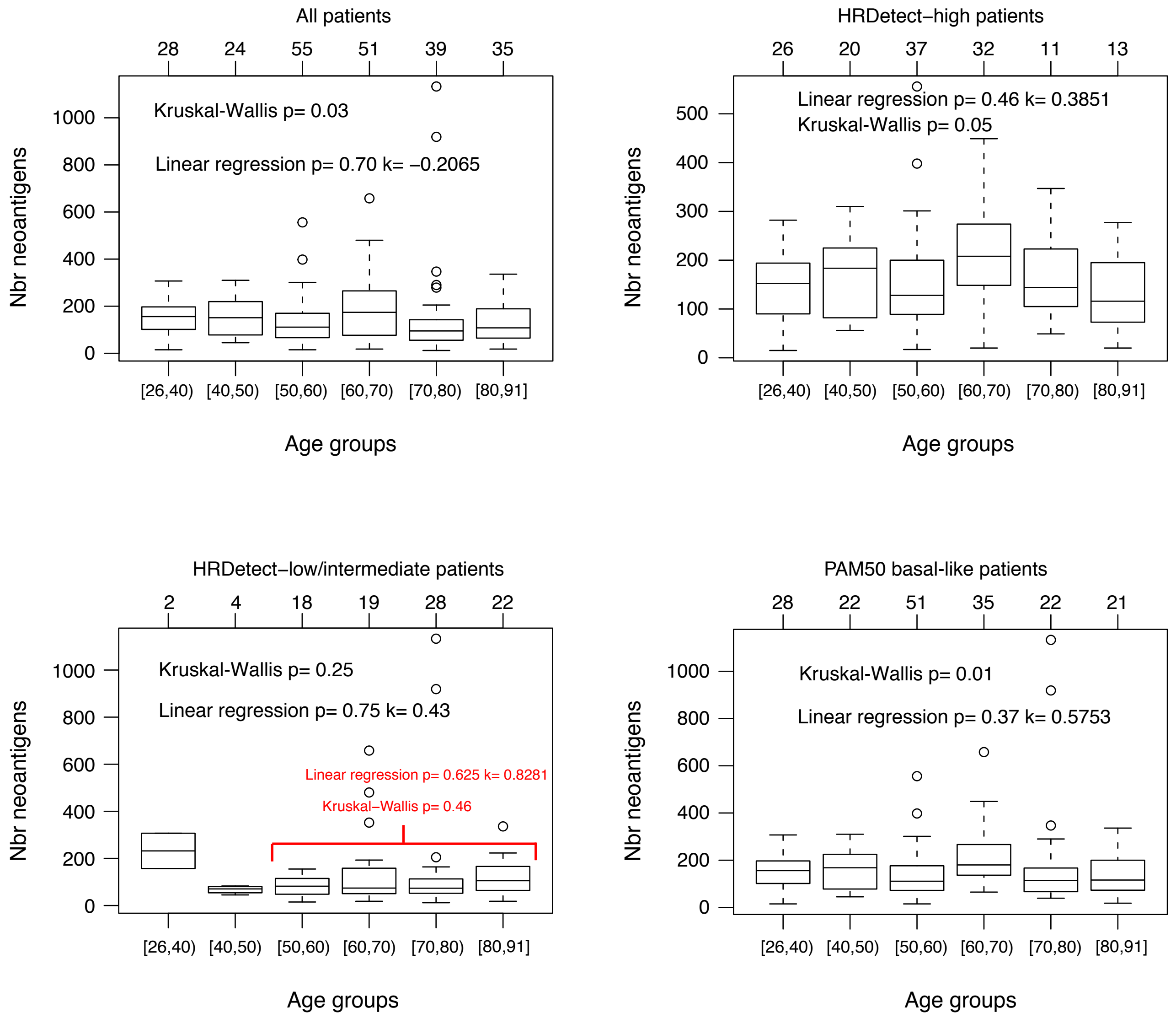
All patients
28 24 55 51 39 35
Kruskal-Wallis p= 0.03
Linear regression p= 0.70 k= −0.2065
Nbr neoantigens
0 200 400 600 800 1000
[26,40) [40,50) [50,60) [60,70) [70,80) [80,91]
Age groups
HRDetect−high patients
26 20 37 32 11 13
Linear regression p= 0.46 k= 0.3851
Kruskal-Wallis p= 0.05
Nbr neoantigens
0 100 200 300 400 500
[26,40) [40,50) [50,60) [60,70) [70,80) [80,91]
Age groups
HRDetect−low/intermediate patients
2 4 18 19 28 22
Kruskal-Wallis p= 0.25
Linear regression p= 0.75 k= 0.43
Linear regression p= 0.625 k= 0.8281
Kruskal−Wallis p= 0.46
Nbr neoantigens
0 200 400 600 800 1000
[26,40) [40,50) [50,60) [60,70) [70,80) [80,91]
Age groups
PAM50 basal-like patients
28 22 51 35 22 21
Kruskal-Wallis p= 0.01
Linear regression p= 0.37 k= 0.5753
Nbr neoantigens
0 200 400 600 800 1000
[26,40) [40,50) [50,60) [60,70) [70,80) [80,91]
Age groups

Q)

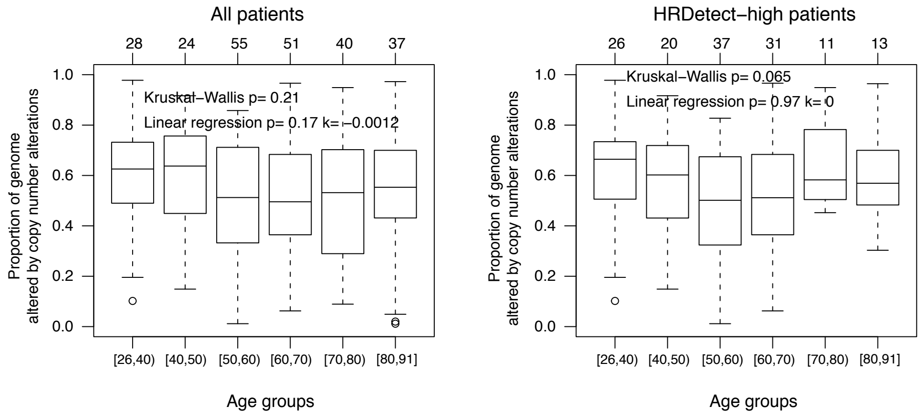
All patients
28 24 55 51 40 37
Kruskal–Wallis p= 0.21
Linear regression p= 0.17 k= −0.0012
HRDetect–high patients
26 20 37 31 11 13
Kruskal–Wallis p= 0.065
Linear regression p= 0.97 k= 0
HRDetect–low patients
2 3 15 19 27 18
Kruskal–Wallis p= 0.60
Linear regression p= 0.30 k= −0.0022
PAM50 basal-like
28 22 51 36 22 23
Kruskal–Wallis p= 0.37
Linear regression p= 0.85 k= −2e−04
Proportion of genome altered by copy number alterations
0.0 0.2 0.4 0.6 0.8 1.0
[26,40) [40,50) [50,60) [60,70) [70,80) [80,91]
Age groups

R)

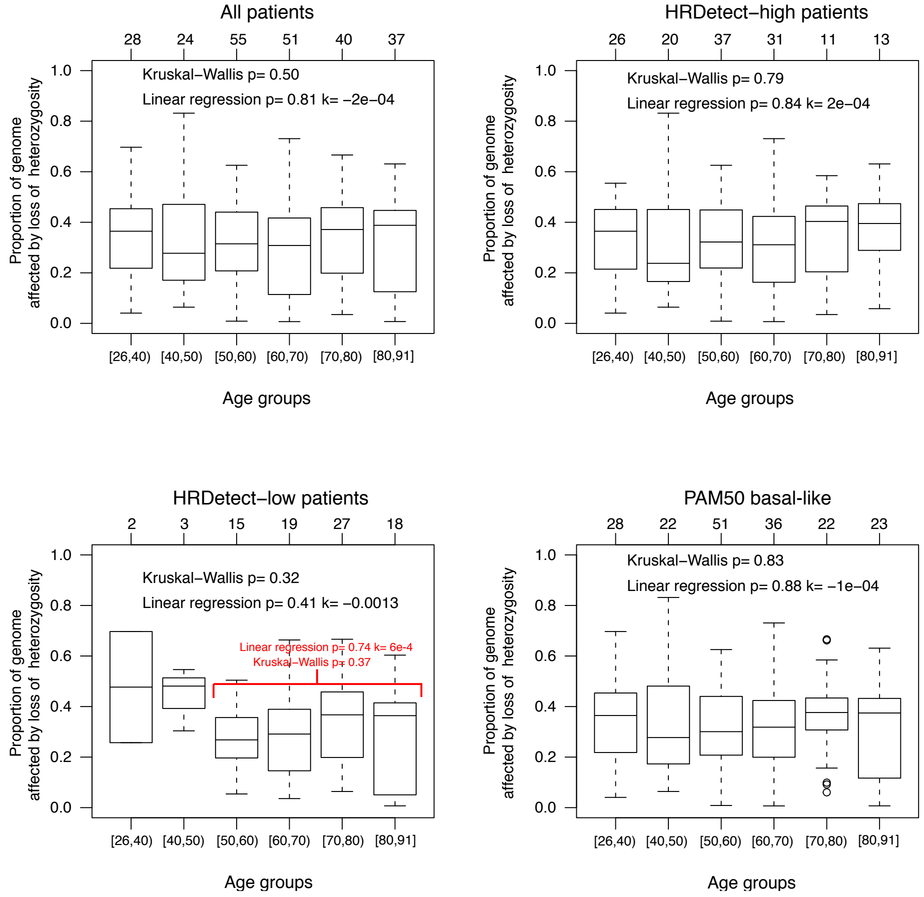
All patients
28 24 55 51 40 37
Kruskal–Wallis p= 0.50
Linear regression p= 0.81 k= −2e−04
HRDetect–high patients
26 20 37 31 11 13
Kruskal–Wallis p= 0.79
Linear regression p= 0.84 k= 2e−04
HRDetect–low patients
2 3 15 19 27 18
Kruskal–Wallis p= 0.32
Linear regression p= 0.41 k= −0.0013
Linear regression p= 0.74 k= 6e-4
Kruskal–Wallis p= 0.37
PAM50 basal-like
28 22 51 36 22 23
Kruskal–Wallis p= 0.83
Linear regression p= 0.88 k= −1e−04
Proportion of genome affected by loss of heterozygosity
0.0 0.2 0.4 0.6 0.8 1.0
[26,40) [40,50) [50,60) [60,70) [70,80) [80,91]
Age groups

S)

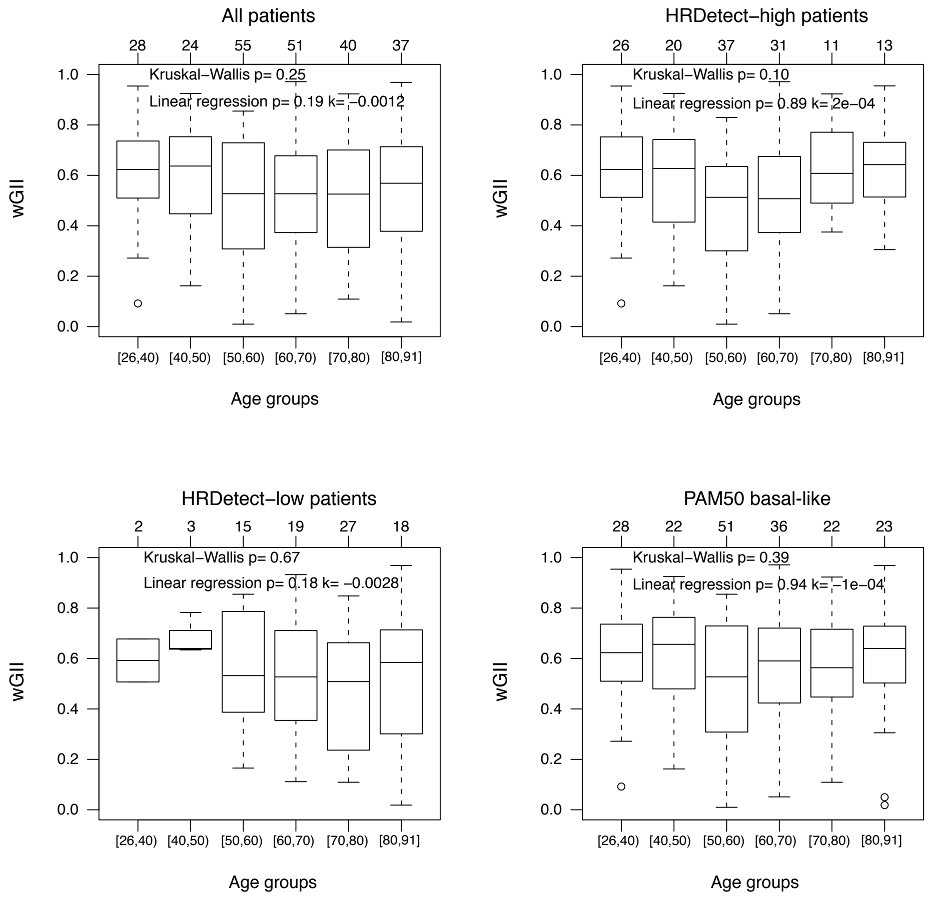
All patients
28 24 55 51 40 37
Kruskal–Wallis p= 0.25
Linear regression p= 0.19 k= –0.0012
wGII
[26,40) [40,50) [50,60) [60,70) [70,80) [80,91]
Age groups
HRDetect–high patients
26 20 37 31 11 13
Kruskal–Wallis p= 0.10
Linear regression p= 0.89 k= 2e–04
wGII
[26,40) [40,50) [50,60) [60,70) [70,80) [80,91]
Age groups
HRDetect–low patients
2 3 15 19 27 18
Kruskal–Wallis p= 0.67
Linear regression p= 0.18 k= –0.0028
wGII
[26,40) [40,50) [50,60) [60,70) [70,80) [80,91]
Age groups
PAM50 basal-like
28 22 51 36 22 23
Kruskal–Wallis p= 0.39
Linear regression p= 0.94 k= –1e–04
wGII
[26,40) [40,50) [50,60) [60,70) [70,80) [80,91]
Age groups

T)

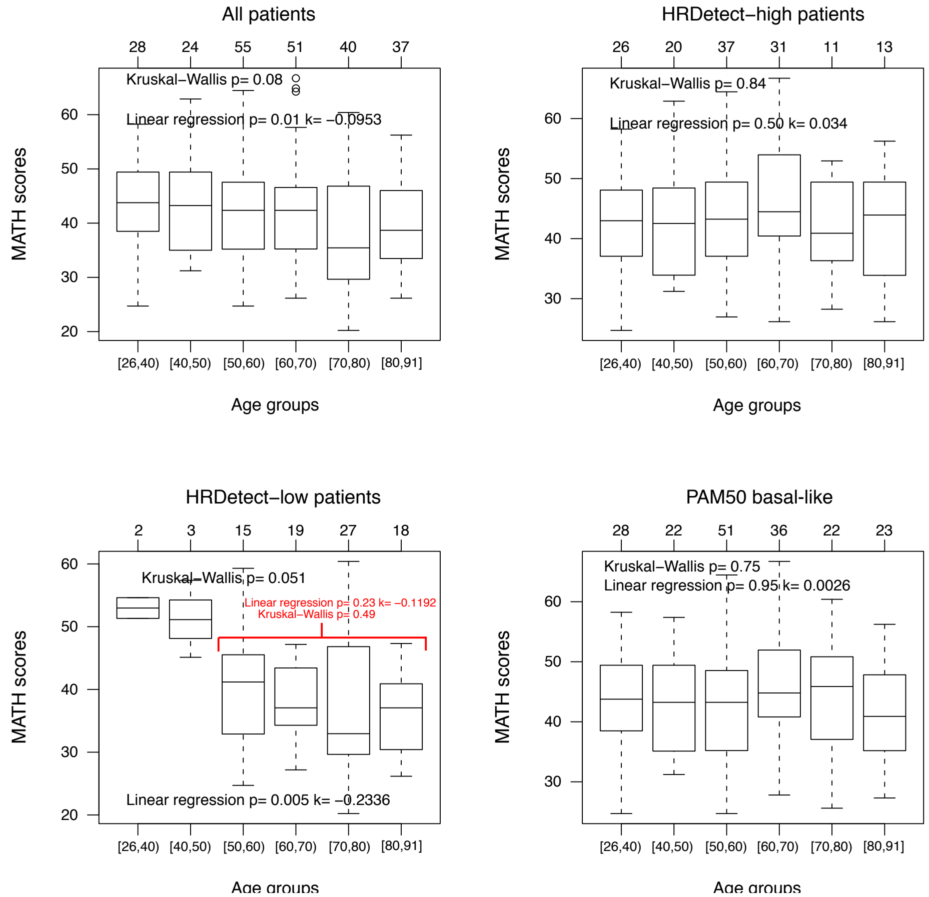
All patients
28 24 55 51 40 37
Kruskal–Wallis p= 0.08
Linear regression p= 0.01 k= –0.0953
MATH scores
[26,40) [40,50) [50,60) [60,70) [70,80) [80,91]
Age groups
HRDetect–high patients
26 20 37 31 11 13
Kruskal–Wallis p= 0.84
Linear regression p= 0.50 k= 0.034
MATH scores
[26,40) [40,50) [50,60) [60,70) [70,80) [80,91]
Age groups
HRDetect–low patients
2 3 15 19 27 18
Kruskal–Wallis p= 0.051
Linear regression p= 0.23 k= –0.1192
Kruskal–Wallis p= 0.49
Linear regression p= 0.005 k= –0.2336
MATH scores
[26,40) [40,50) [50,60) [60,70) [70,80) [80,91]
Age groups
PAM50 basal-like
28 22 51 36 22 23
Kruskal–Wallis p= 0.75
Linear regression p= 0.95 k= 0.0026
MATH scores
[26,40) [40,50) [50,60) [60,70) [70,80) [80,91]
Age groups

U)

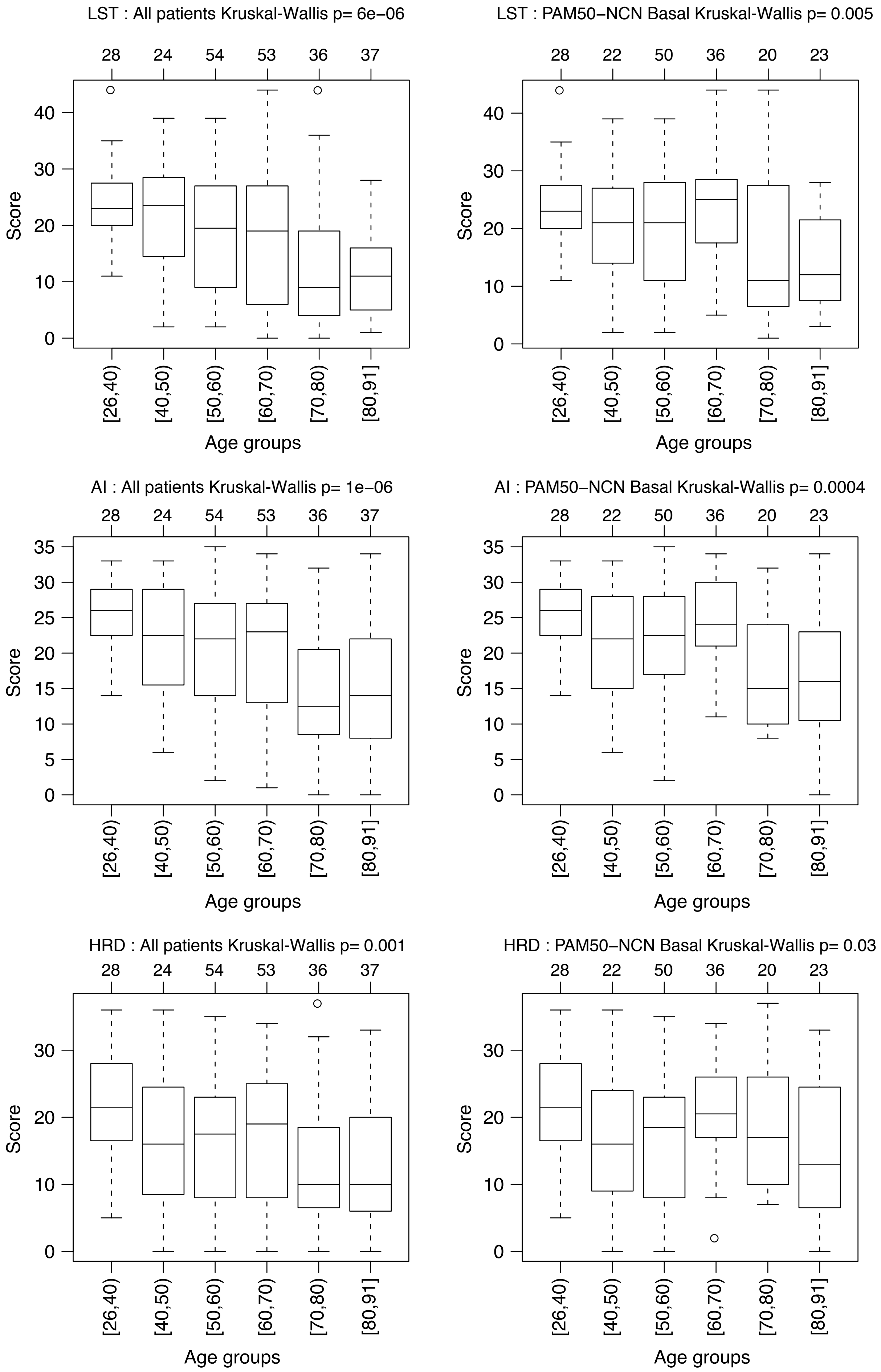

LST : All patients Kruskal-Wallis p= 6e−06
LST : PAM50−NCN Basal Kruskal-Wallis p= 0.005
AI : All patients Kruskal-Wallis p= 1e−06
AI : PAM50−NCN Basal Kruskal-Wallis p= 0.0004
HRD : All patients Kruskal-Wallis p= 0.001
HRD : PAM50−NCN Basal Kruskal-Wallis p= 0.03
28 24 54 53 36 37
28 22 50 36 20 23
Score
Age groups
[26,40) [40,50) [50,60) [60,70) [70,80) [80,91]
