# Supplementary material for: Molecular analyses of triple-negative breast cancer in the young and elderly
Source: Breast Cancer Res. 2021 Feb 10;23:20. doi: 10.1186/s13058-021-01392-0 (PMC7874480; doi:10.1186/s13058-021-01392-0)
Supplement: Supplementary file 3 — Additional file 3. A PDF file with supplementary Figure 2 showing copy number alterations in subgroups of TNBC. [file 13058_2021_1392_MOESM3_ESM.pdf]

Supplementary Figure 2. Copy number alterations (CNAs) in specific TNBC subgroups. (**A**) Overall frequency of CNAs in the cohort. Red is frequency of gain, green is frequency of loss. Data is based on ASCAT segmented data analyzed as described in Nik-Zainal et al., Nature 2016. (**B**) Frequency of copy number gain (red) and loss (green) across the genome for the different age groups in SCAN-B patients. (**C**) SCAN-B patients older than 70 years (top) SCAN-B LAR subtype patients (second top panel), and as reference patients with a Luminal B PAM50-AIMS subtype from Nik-Zainal et al. (Nature 2016) and patients with a basal-like PAM50-AIMS subtype (bottom) from Nik-Zainal et al.

A)

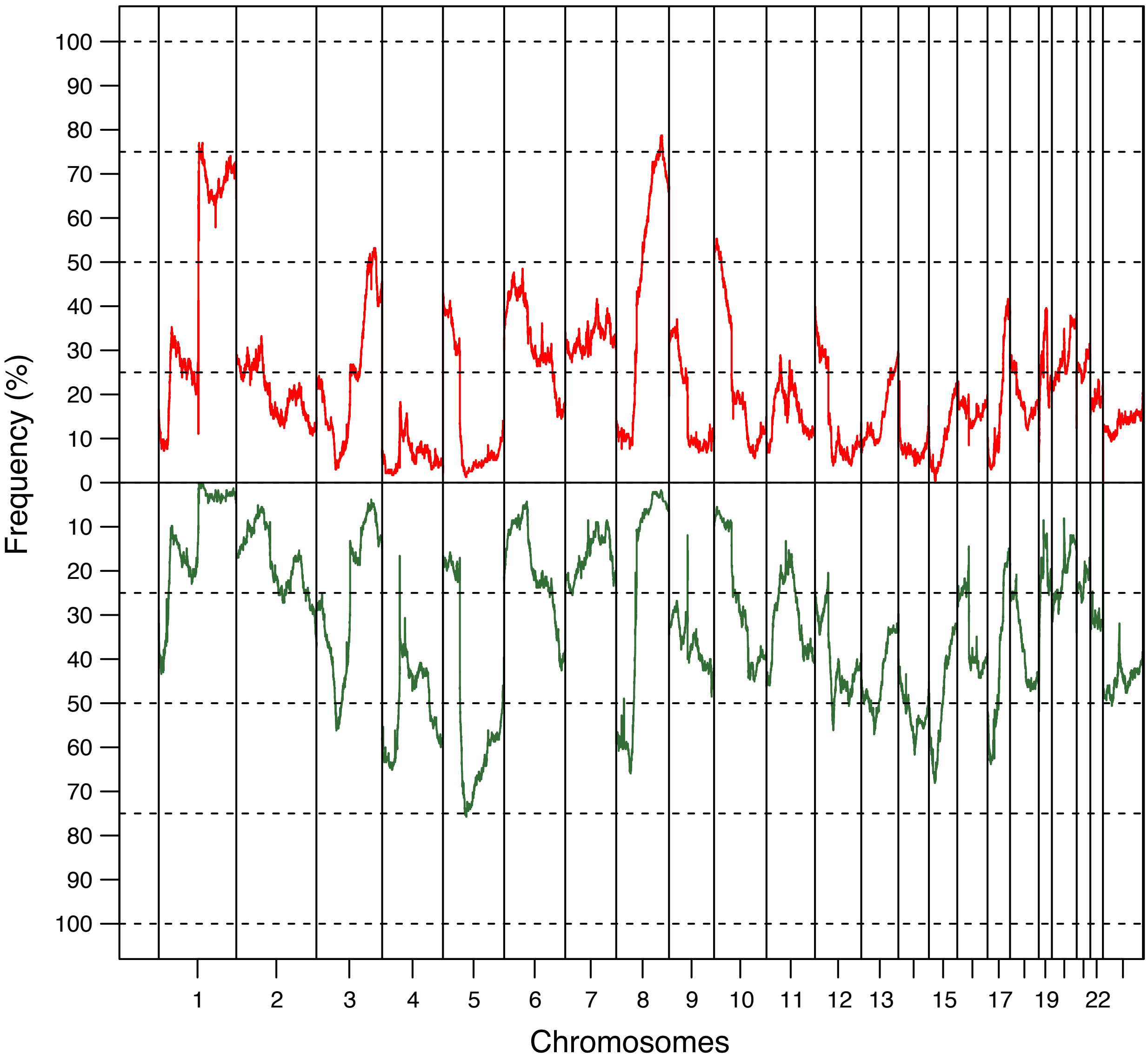

B)

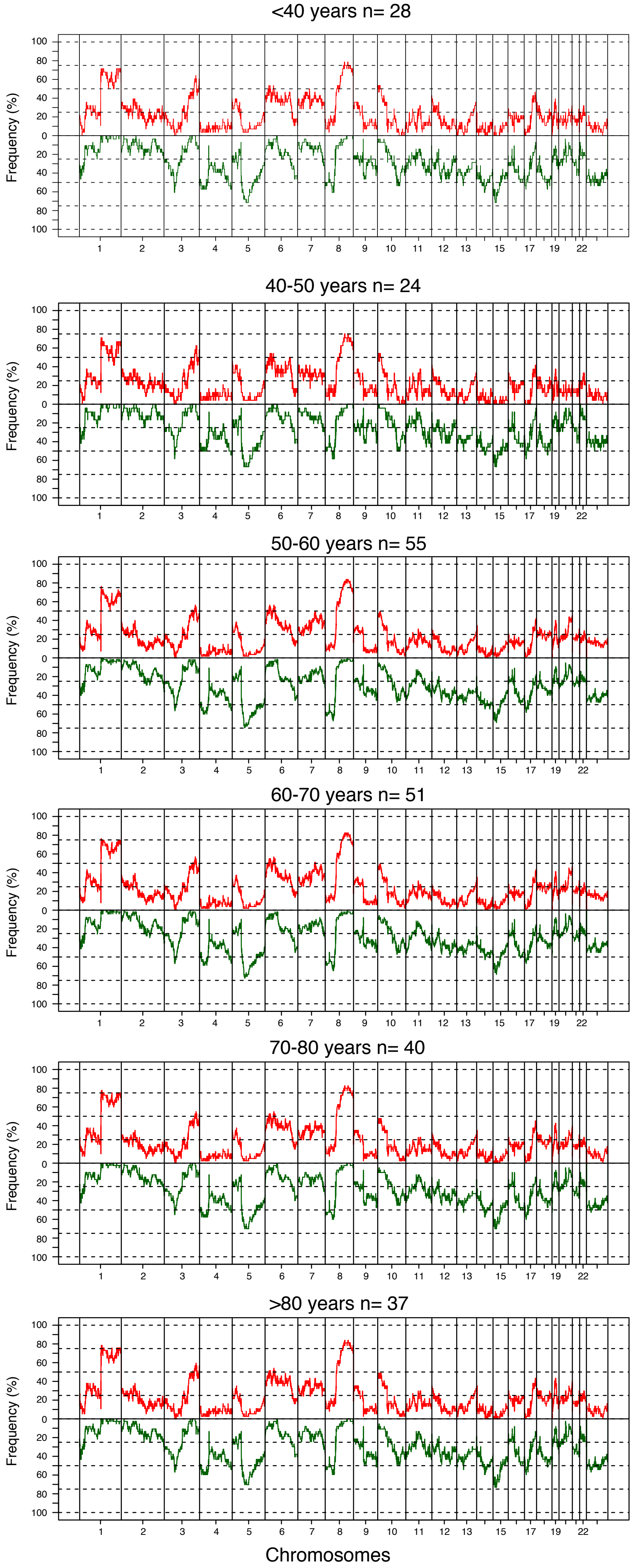

<40 years n= 28
40-50 years n= 24
50-60 years n= 55
60-70 years n= 51
70-80 years n= 40
>80 years n= 37
Frequency (%)
100
80
60
40
20
0
1
2
3
4
5
6
7
8
9
10
11
12
13
15
17
19
22
Chromosomes

C)

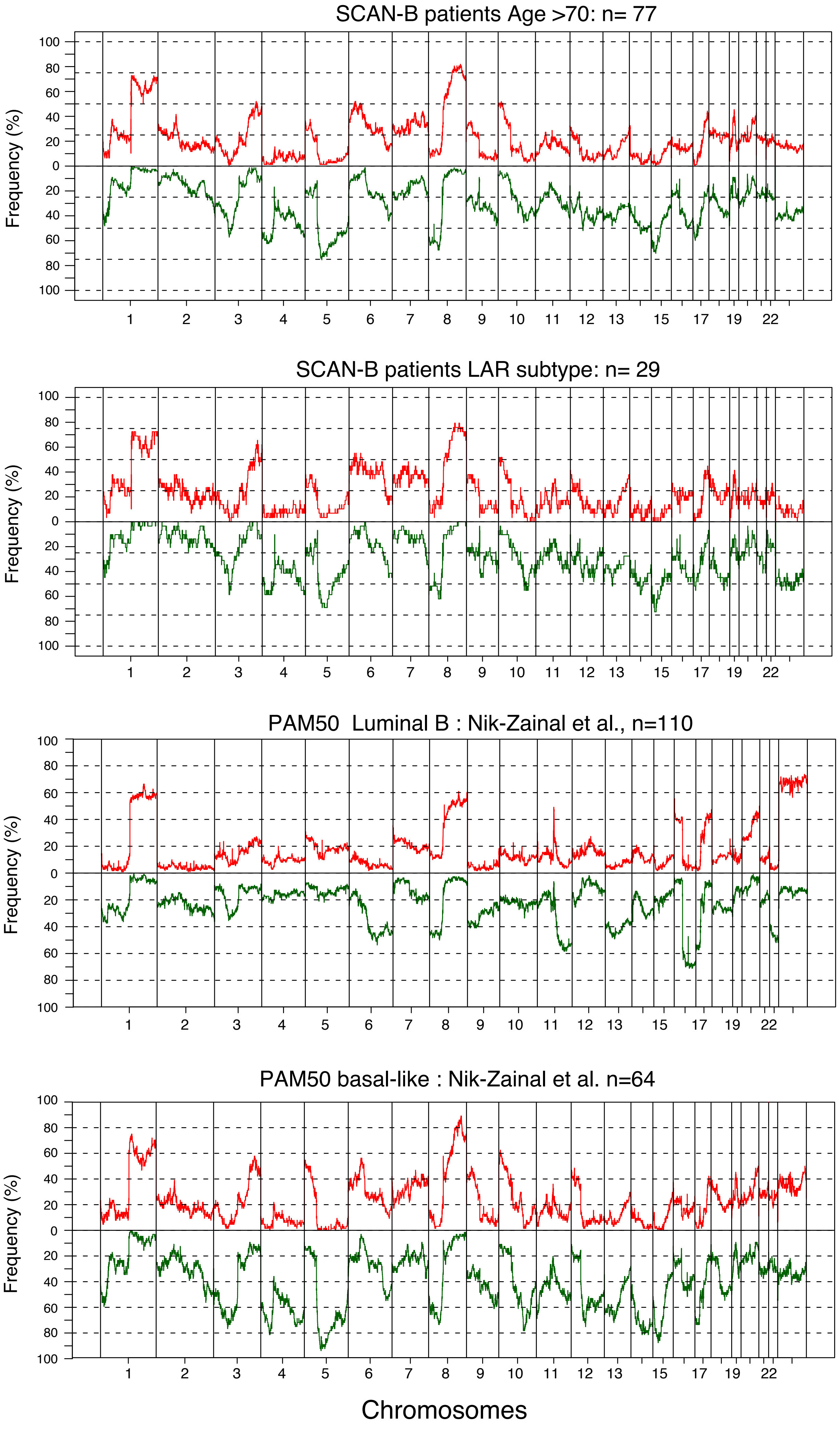
SCAN-B patients Age >70: n= 77
SCAN-B patients LAR subtype: n= 29
PAM50 Luminal B : Nik-Zainal et al., n=110
PAM50 basal-like : Nik-Zainal et al. n=64
Frequency (%)
100
80
60
40
20
0
1
2
3
4
5
6
7
8
9
10
11
12
13
15
17
19
22
Chromosomes
