# Supplementary material for: Molecular analyses of triple-negative breast cancer in the young and elderly
Source: Breast Cancer Res. 2021 Feb 10;23:20. doi: 10.1186/s13058-021-01392-0 (PMC7874480; doi:10.1186/s13058-021-01392-0)
Supplement: Supplementary file 4 — Additional file 4. A PDF file with supplementary Figure 3 showing mutational and rearrangement signatures in SCAN-B TNBCs. [file 13058_2021_1392_MOESM4_ESM.pdf]

Supplementary Figure 3. Mutational and rearrangement signatures in SCAN-B samples. (**A**) Scatter plots of mutational signature 1 (left) and 5 (right) proportions versus age at diagnosis for all 237 SCAN-B cases. Signature proportions were from substitution data refitted with SigFit. Red line corresponds to a linear regression fit. (**B**) Proportions of different indel types in BRCA1-deficient (inactivation by somatic/germline mutations or hypermethylation) and HRDetect-low/intermediate SCAN-B cases versus stratified age groups. Top axes indicate group sizes. Data obtained from Staaf et al. Nature Medicine 2019. (**C**) Proportions of six rearrangement signatures defined by Nik-Zainal et al. Nature 2016 in BRCA1-deficient and HRDetect-low/intermediate SCAN-B cases versus stratified age groups. Top axes indicate group sizes. Data obtained from Staaf et al. Nature Medicine 2019. (**D**) Proportions of 11 mutational signatures in BRCA1-deficient and HRDetect-low/intermediate SCAN-B cases versus stratified age groups. Top axes indicate group sizes. Proportions come from SigFit refitted substitutions originally reported by Staaf et al. Nature Medicine 2019. (**E**) Scatter plots of proportions of 11 mutational signatures from D versus patient age at diagnosis in BRCA1-deficient cases. Red line corresponds to a linear regression fit, with p-value and slope (k) values supplied. (**F**) Similar scatter plot as in E but for HRDetect-low/intermediate cases. (**G**) Proportions of deletions with microhomology for PAM50 basal-like (left), IntClust10 cluster 10 cases (center), and TNBCtype BL1 cases (right) versus stratified age groups. P-values calculated using Kruskal-Wallis test. (**H**) Proportions of mutational signature 3 for the same groups as in G. (**I**) Proportions of rearrangement signature 3 for the same groups as in G.

For the age group definitions these are indicated as "[" meaning equal or greater than, ")" meaning smaller than, or "]" meaning smaller or equal than the value specified next to it.

A)

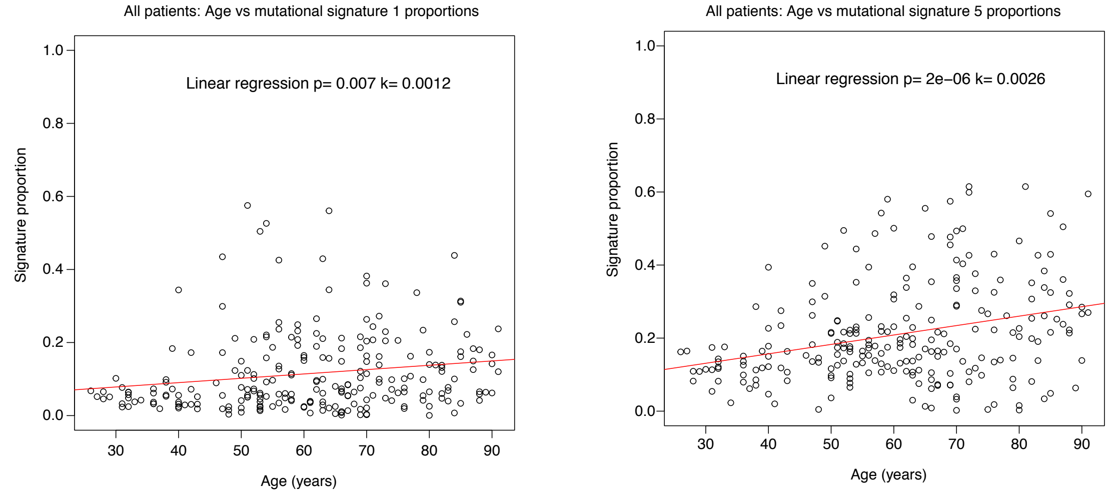

B)

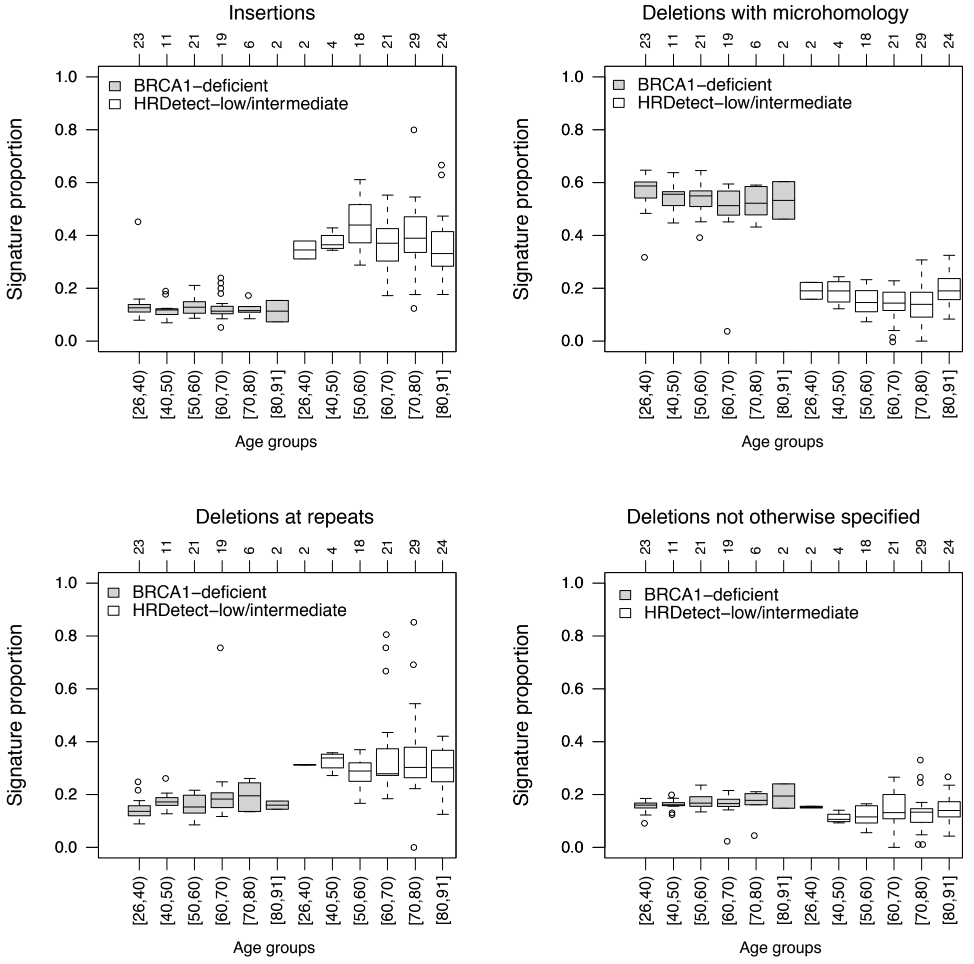

C)

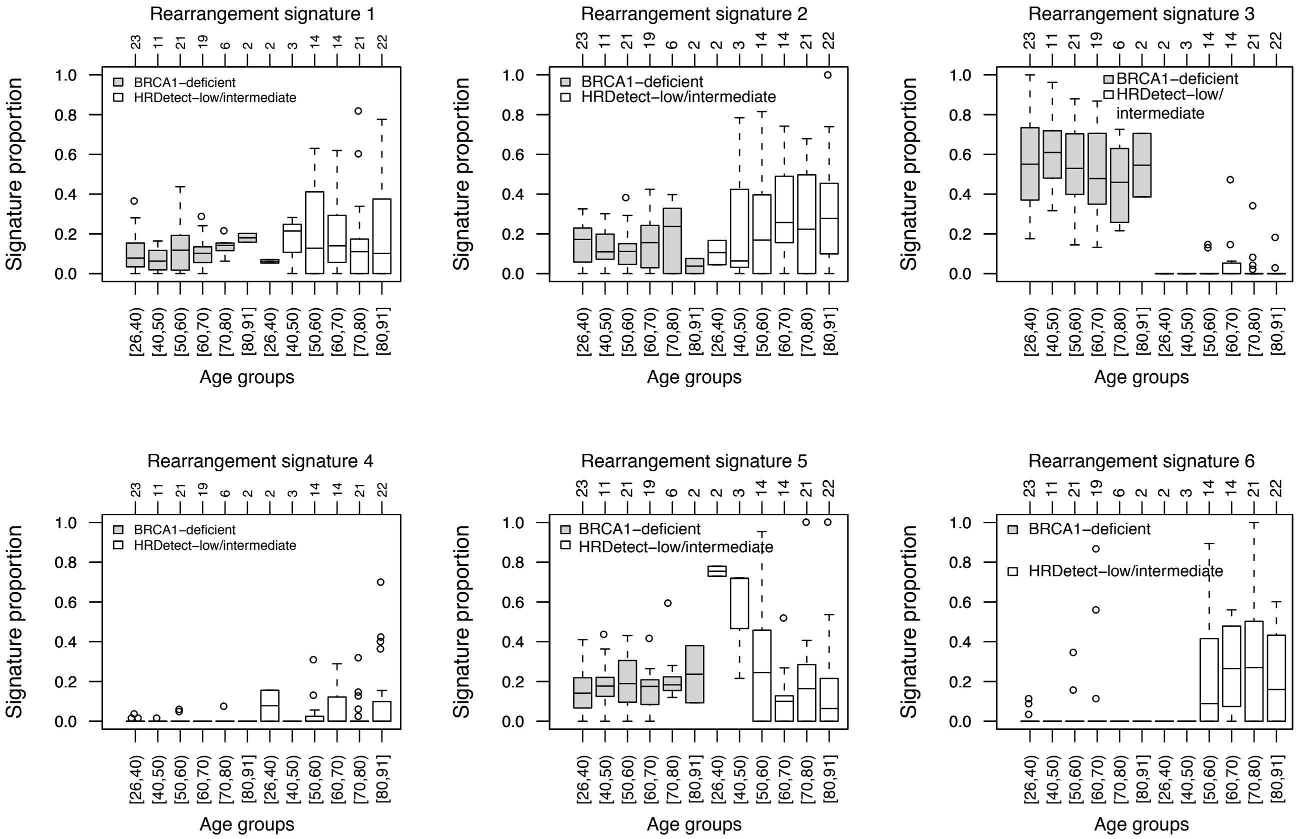

Rearrangement signature 1
Rearrangement signature 2
Rearrangement signature 3
Rearrangement signature 4
Rearrangement signature 5
Rearrangement signature 6
BRCA1−deficient
HRDetect−low/intermediate
Signature proportion
Age groups
[26,40)
[40,50)
[50,60)
[60,70)
[70,80)
[80,91]

D)

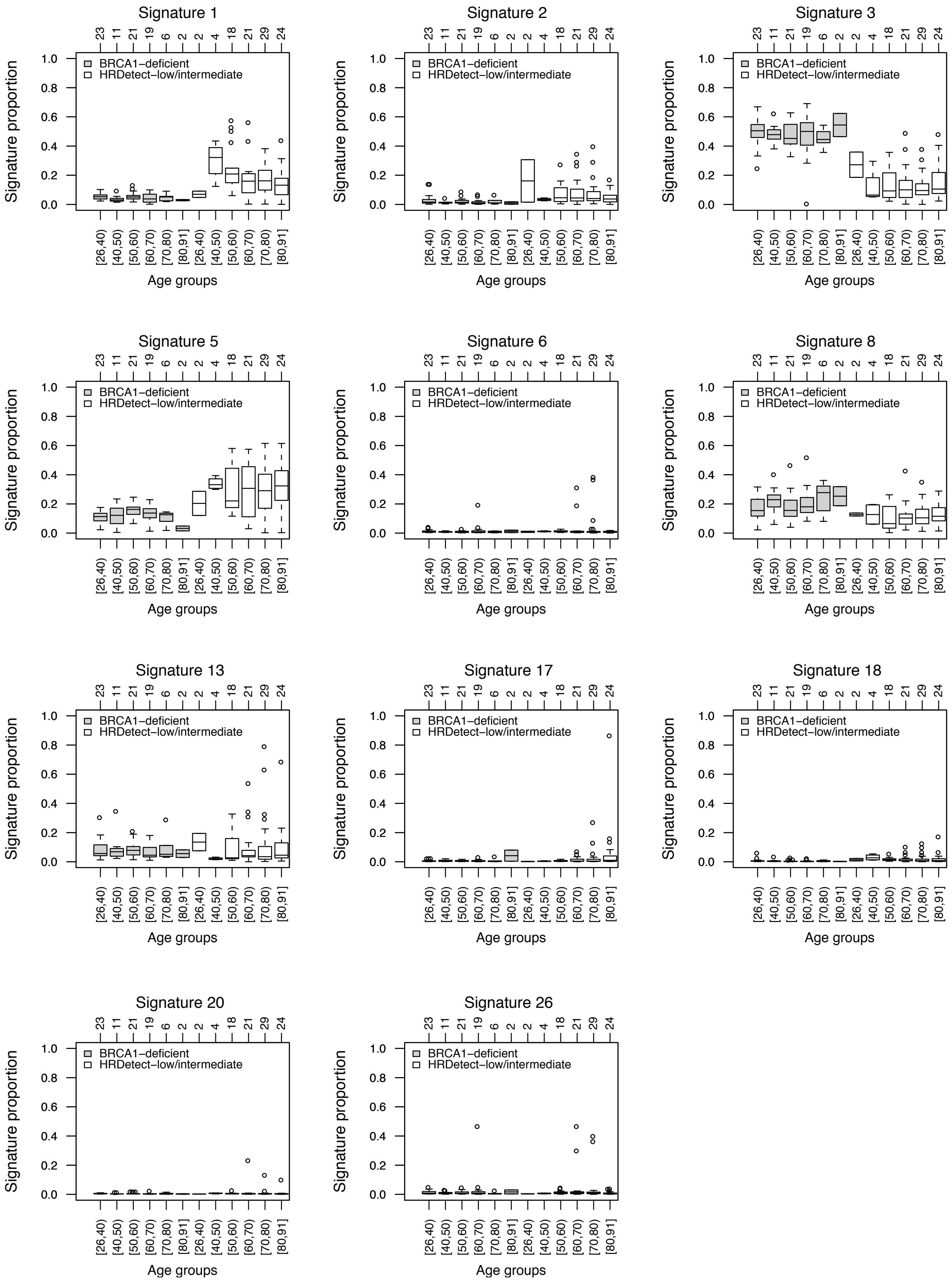
Signature 1
Signature 2
Signature 3
Signature 5
Signature 6
Signature 8
Signature 13
Signature 17
Signature 18
Signature 20
Signature 26
BRCA1−deficient
HRDetect−low/intermediate
Signature proportion
Age groups
23 11 21 19 6 2 2 4 18 21 29 24
[26,40) [40,50) [50,60) [60,70) [70,80) [80,91] [26,40) [40,50) [50,60) [60,70) [70,80) [80,91]
0.0 0.2 0.4 0.6 0.8 1.0

E)

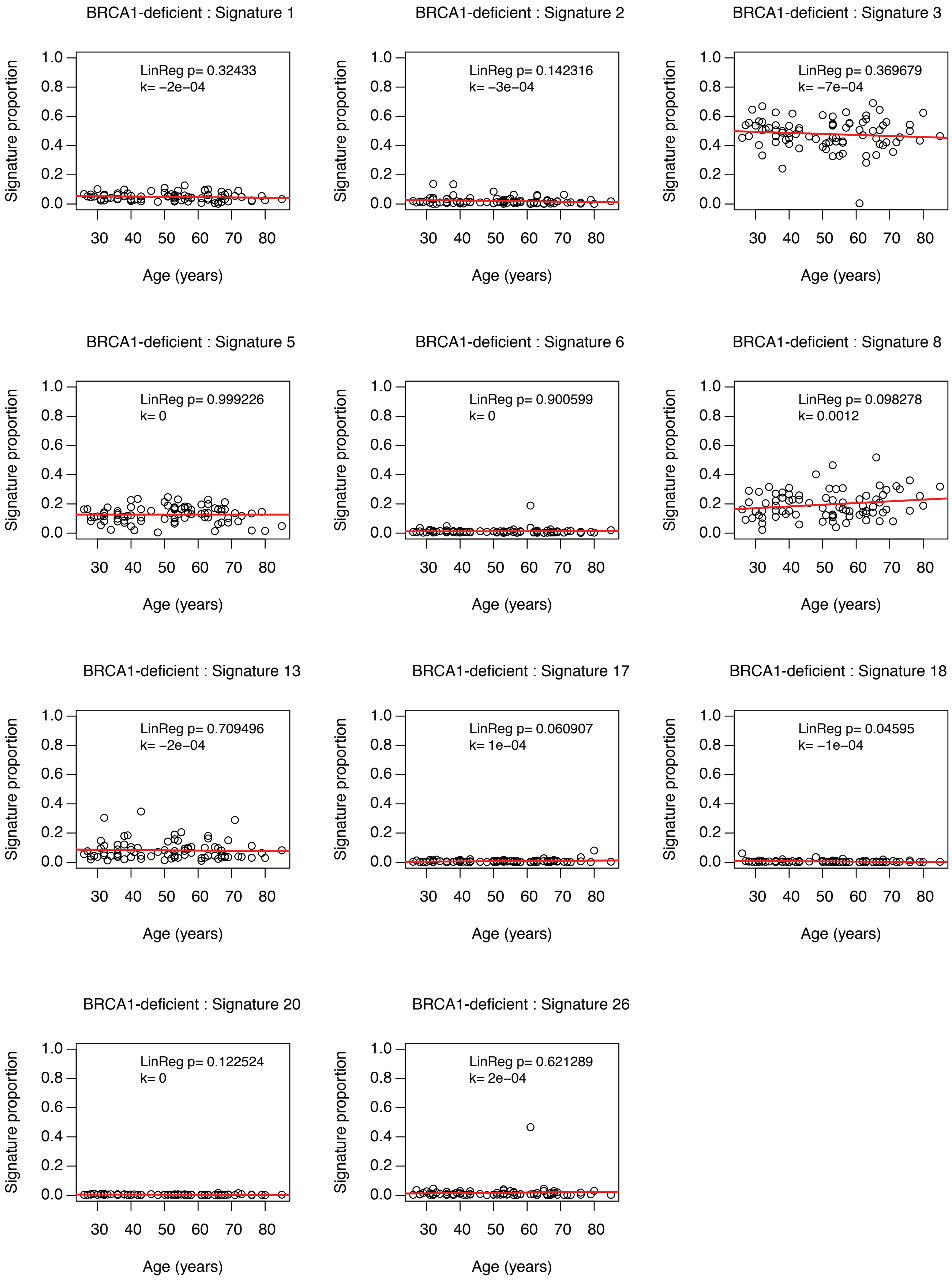
BRCA1-deficient : Signature 1
LinReg p= 0.32433
k= −2e−04
BRCA1-deficient : Signature 2
LinReg p= 0.142316
k= −3e−04
BRCA1-deficient : Signature 3
LinReg p= 0.369679
k= −7e−04
BRCA1-deficient : Signature 5
LinReg p= 0.999226
k= 0
BRCA1-deficient : Signature 6
LinReg p= 0.900599
k= 0
BRCA1-deficient : Signature 8
LinReg p= 0.098278
k= 0.0012
BRCA1-deficient : Signature 13
LinReg p= 0.709496
k= −2e−04
BRCA1-deficient : Signature 17
LinReg p= 0.060907
k= 1e−04
BRCA1-deficient : Signature 18
LinReg p= 0.04595
k= −1e−04
BRCA1-deficient : Signature 20
LinReg p= 0.122524
k= 0
BRCA1-deficient : Signature 26
LinReg p= 0.621289
k= 2e−04
Signature proportion
Age (years)
0.0
0.2
0.4
0.6
0.8
1.0
30
40
50
60
70
80

F)

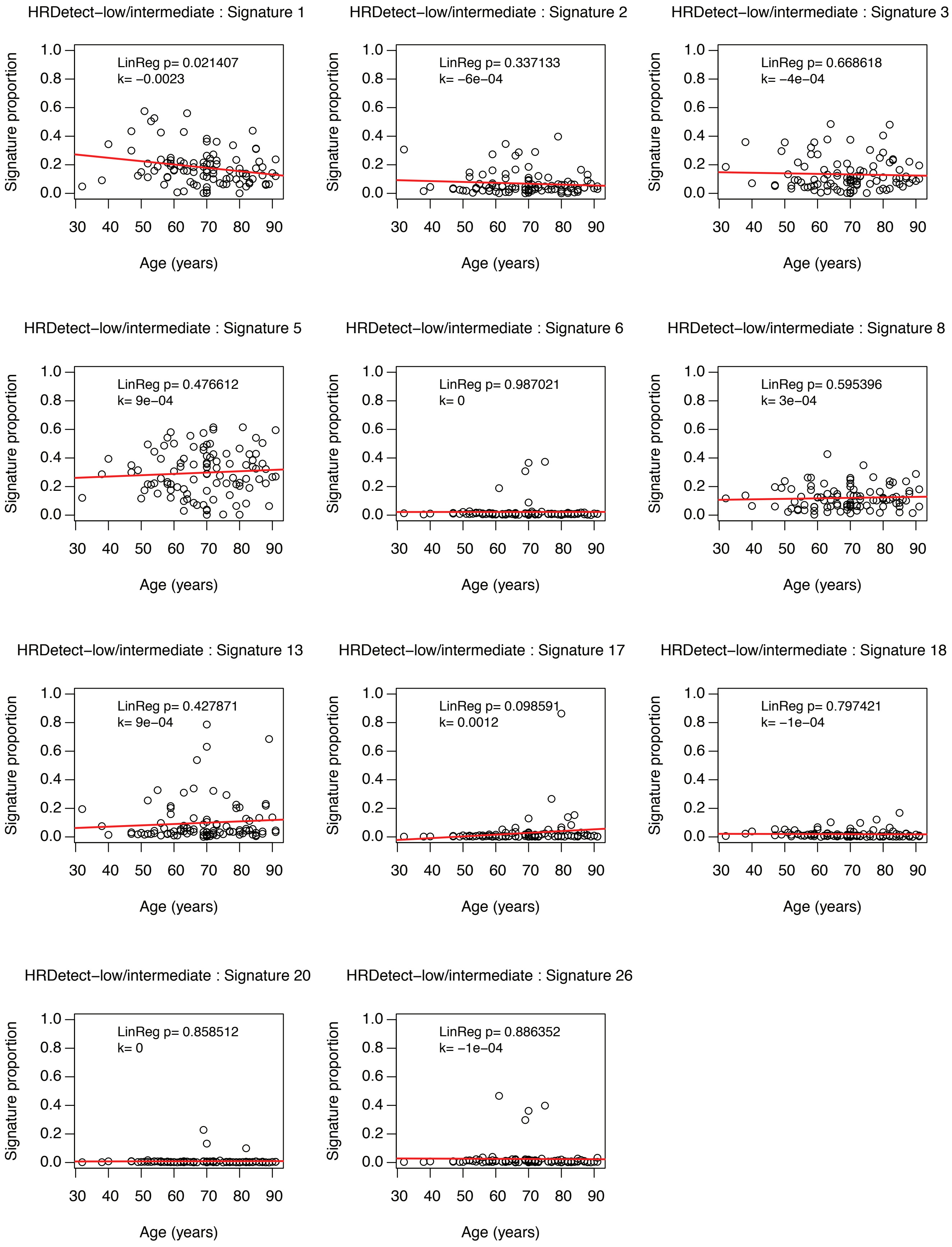
HRDetect−low/intermediate : Signature 1
LinReg p= 0.021407
k= −0.0023
HRDetect−low/intermediate : Signature 2
LinReg p= 0.337133
k= −6e−04
HRDetect−low/intermediate : Signature 3
LinReg p= 0.668618
k= −4e−04
HRDetect−low/intermediate : Signature 5
LinReg p= 0.476612
k= 9e−04
HRDetect−low/intermediate : Signature 6
LinReg p= 0.987021
k= 0
HRDetect−low/intermediate : Signature 8
LinReg p= 0.595396
k= 3e−04
HRDetect−low/intermediate : Signature 13
LinReg p= 0.427871
k= 9e−04
HRDetect−low/intermediate : Signature 17
LinReg p= 0.098591
k= 0.0012
HRDetect−low/intermediate : Signature 18
LinReg p= 0.797421
k= −1e−04
HRDetect−low/intermediate : Signature 20
LinReg p= 0.858512
k= 0
HRDetect−low/intermediate : Signature 26
LinReg p= 0.886352
k= −1e−04
Signature proportion
Age (years)

G)

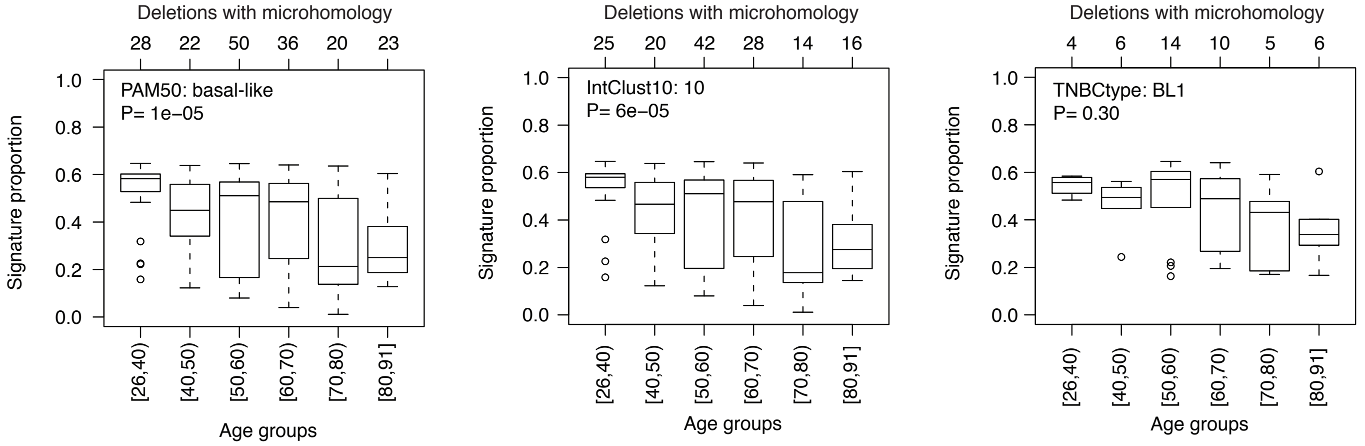
Deletions with microhomology
28 22 50 36 20 23
PAM50: basal-like
P= 1e−05
Signature proportion
0.0 0.2 0.4 0.6 0.8 1.0
[26,40) [40,50) [50,60) [60,70) [70,80) [80,91]
Age groups
Deletions with microhomology
25 20 42 28 14 16
IntClust10: 10
P= 6e−05
Signature proportion
Age groups
Deletions with microhomology
4 6 14 10 5 6
TNBCtype: BL1
P= 0.30
Signature proportion
Age groups

H)

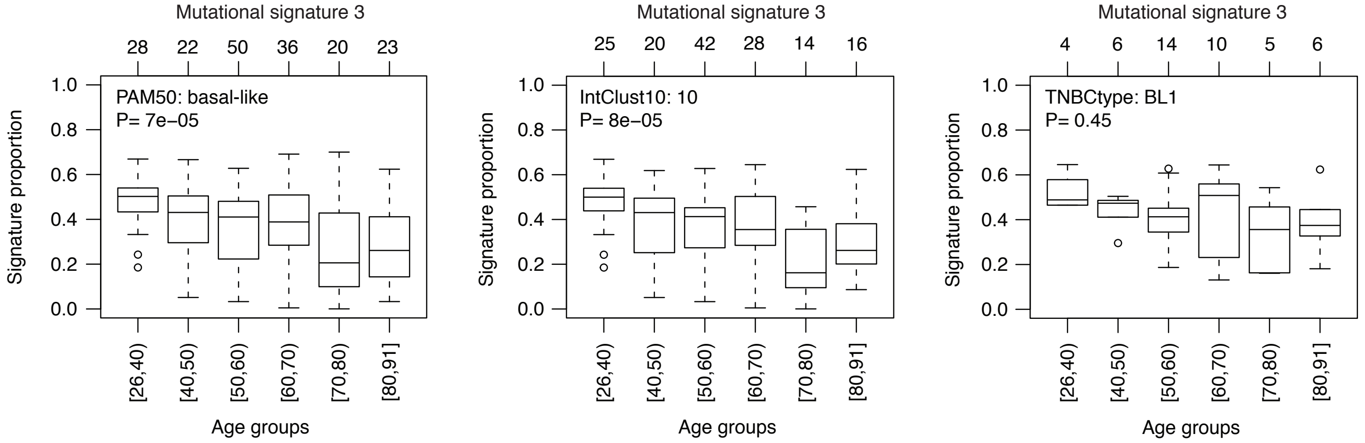
Mutational signature 3
28 22 50 36 20 23
PAM50: basal-like
P= 7e−05
Signature proportion
0.0 0.2 0.4 0.6 0.8 1.0
[26,40) [40,50) [50,60) [60,70) [70,80) [80,91]
Age groups
Mutational signature 3
25 20 42 28 14 16
IntClust10: 10
P= 8e−05
Signature proportion
Age groups
Mutational signature 3
4 6 14 10 5 6
TNBCtype: BL1
P= 0.45
Signature proportion
Age groups

I)

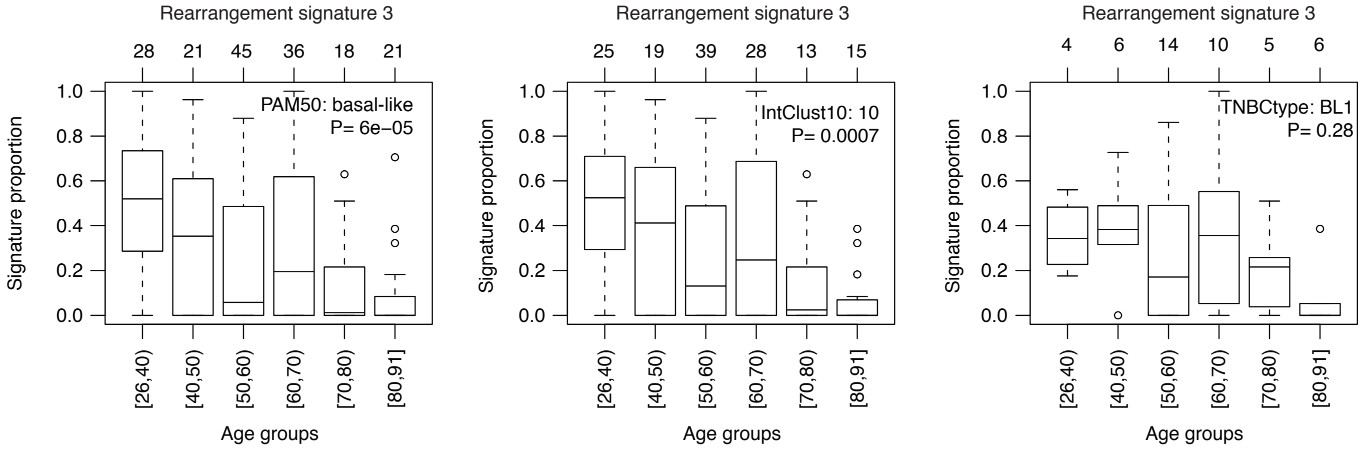
Rearrangement signature 3
28 21 45 36 18 21
PAM50: basal-like
P= 6e−05
Signature proportion
0.0 0.2 0.4 0.6 0.8 1.0
[26,40) [40,50) [50,60) [60,70) [70,80) [80,91]
Age groups
Rearrangement signature 3
25 19 39 28 13 15
IntClust10: 10
P= 0.0007
Signature proportion
Age groups
Rearrangement signature 3
4 6 14 10 5 6
TNBCtype: BL1
P= 0.28
Signature proportion
Age groups
