# Supplementary material for: Molecular analyses of triple-negative breast cancer in the young and elderly
Source: Breast Cancer Res. 2021 Feb 10;23:20. doi: 10.1186/s13058-021-01392-0 (PMC7874480; doi:10.1186/s13058-021-01392-0)
Supplement: Supplementary file 5 — Additional file 5. A PDF file with supplementary Figure 4 showing molecular characteristics of genetic subtypes of SCAN-B TNBCs related to the main Fig. 5. [file 13058_2021_1392_MOESM5_ESM.pdf]

Supplementary Figure 4. Molecular characteristics of genetic subtypes defined in Figure 5. SCAN-B tumors were stratified based on BRCA-status and HRDetect status into four genetic subgroups as shown in Figure 5 for which genomic and in situ features were analyzed with respect to defined age groups. (**A**) Gene expression subtype proportions (PAM50, IntClust10, TNBCtype) for the four genetic subtypes. (**B**) Principal component analysis for the four genetic subgroups based on gene expression data for 232 SCAN-B cases using 19102 RefSeq genes. Analyses were performed similarly as in Figure 2C using the R swamp package. In each analysis, different molecular and clinicopathological factors, including age at diagnosis (years: Age) and stratified age groups (10-year intervals: Age groups) were included to assess how strong explainers of the total transcriptional variation they represented. (**C**) Cumulative proportions of PAM50, IntClust10 and TNBCtype subtypes versus age at diagnosis for HRDetect-low/intermediate & non-BRCA1-like cases. Only subtypes with at least seven cases were included. (**D**) Cumulative proportions of TNBCtype subtypes versus age at diagnosis for BRCA1-deficient and HRDetect-high & non-BRCA1-like cases. Only subtypes with at least seven cases were included. (**E**) Distributions of TIL estimates versus age groups for the four genetic subtypes. Trends for BRCA1-deficient and HRDetect-high & non-BRCA-like groups match results shown in Figure 3A for HRDetect-high cases in general.

For the age group definitions these are indicated as "[" meaning equal or greater than, ")" meaning smaller than, or "]" meaning smaller or equal than the value specified next to it.

A)

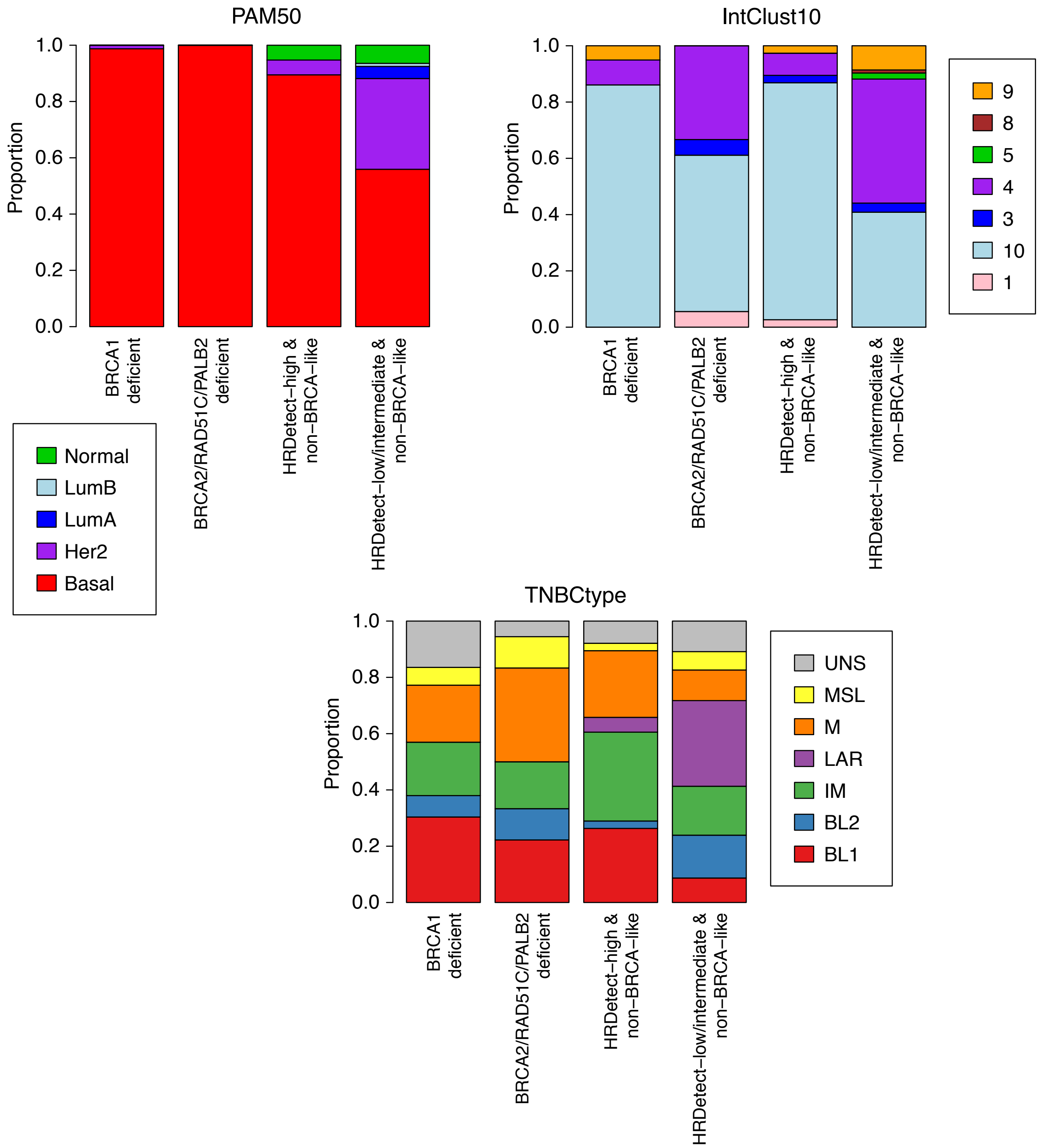

B)

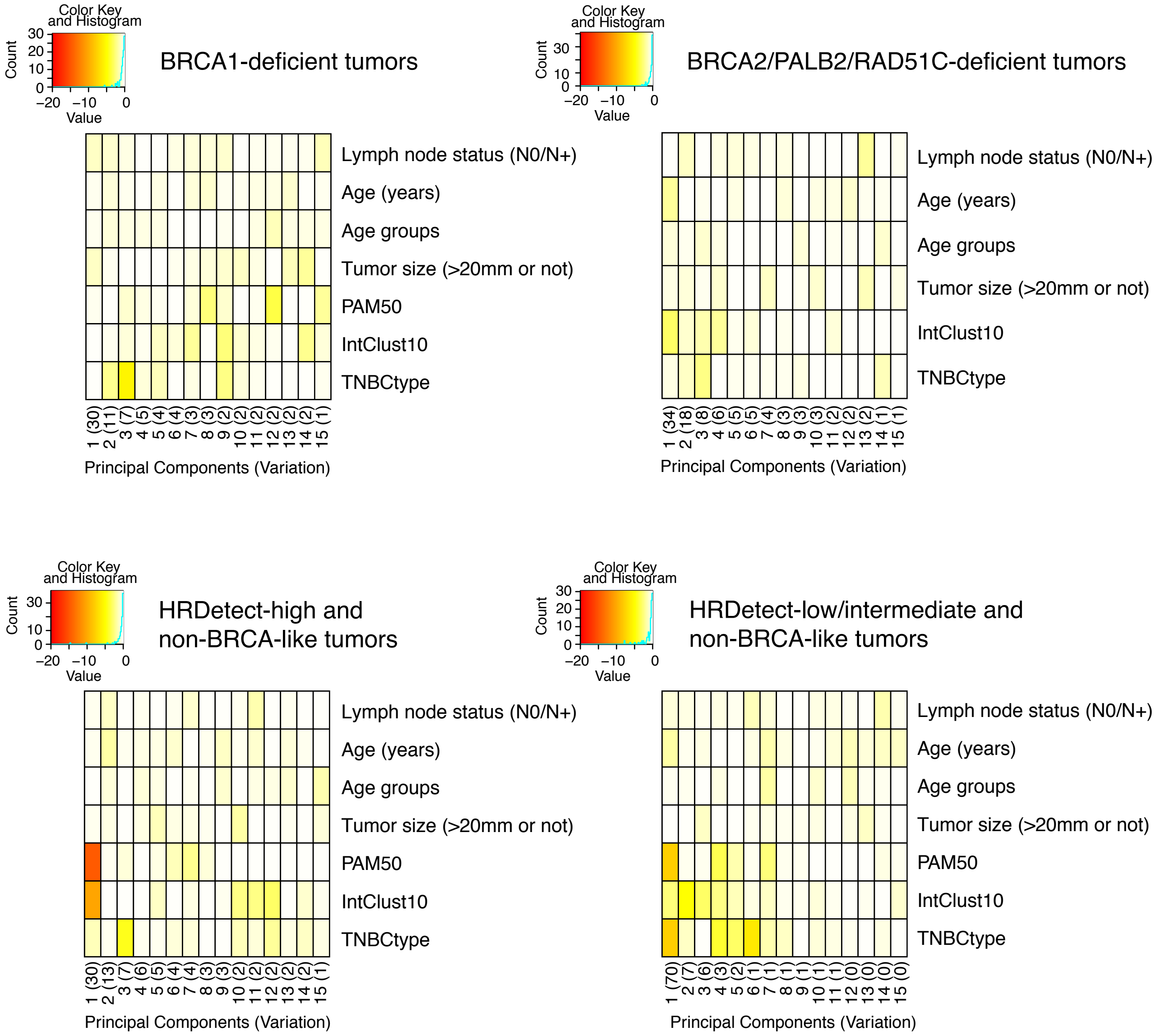
Color Key and Histogram
Count
Value
BRCA1-deficient tumors
BRCA2/PALB2/RAD51C-deficient tumors
HRDetect-high and non-BRCA-like tumors
HRDetect-low/intermediate and non-BRCA-like tumors
Lymph node status (N0/N+)
Age (years)
Age groups
Tumor size (>20mm or not)
PAM50
IntClust10
TNBCtype
Principal Components (Variation)
1 (30) 2 (11) 3 (7) 4 (5) 5 (4) 6 (4) 7 (3) 8 (3) 9 (2) 10 (2) 11 (2) 12 (2) 13 (2) 14 (2) 15 (1)
1 (34) 2 (18) 3 (8) 4 (6) 5 (5) 6 (5) 7 (4) 8 (3) 9 (3) 10 (3) 11 (2) 12 (2) 13 (2) 14 (1) 15 (1)
1 (30) 2 (13) 3 (7) 4 (6) 5 (5) 6 (4) 7 (4) 8 (3) 9 (3) 10 (2) 11 (2) 12 (2) 13 (2) 14 (2) 15 (1)
1 (70) 2 (7) 3 (6) 4 (3) 5 (2) 6 (1) 7 (1) 8 (1) 9 (1) 10 (1) 11 (1) 12 (0) 13 (0) 14 (0) 15 (0)

C)

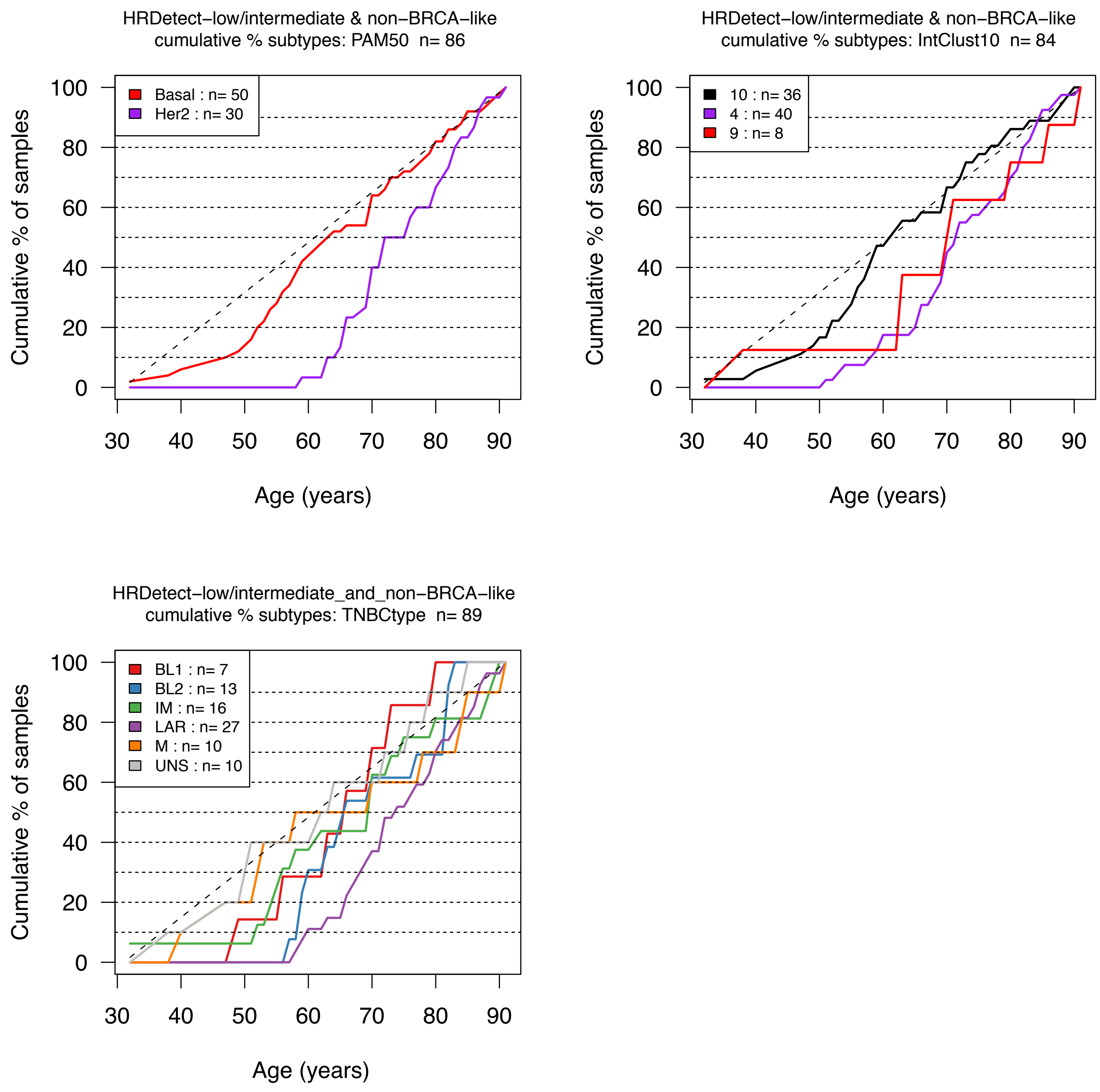
HRDetect−low/intermediate & non−BRCA−like
cumulative % subtypes: PAM50 n= 86
Basal : n= 50
Her2 : n= 30
Cumulative % of samples
Age (years)
HRDetect−low/intermediate & non−BRCA−like
cumulative % subtypes: IntClust10 n= 84
10 : n= 36
4 : n= 40
9 : n= 8
Cumulative % of samples
Age (years)
HRDetect−low/intermediate_and_non−BRCA−like
cumulative % subtypes: TNBCtype n= 89
BL1 : n= 7
BL2 : n= 13
IM : n= 16
LAR : n= 27
M : n= 10
UNS : n= 10
Cumulative % of samples
Age (years)

D)

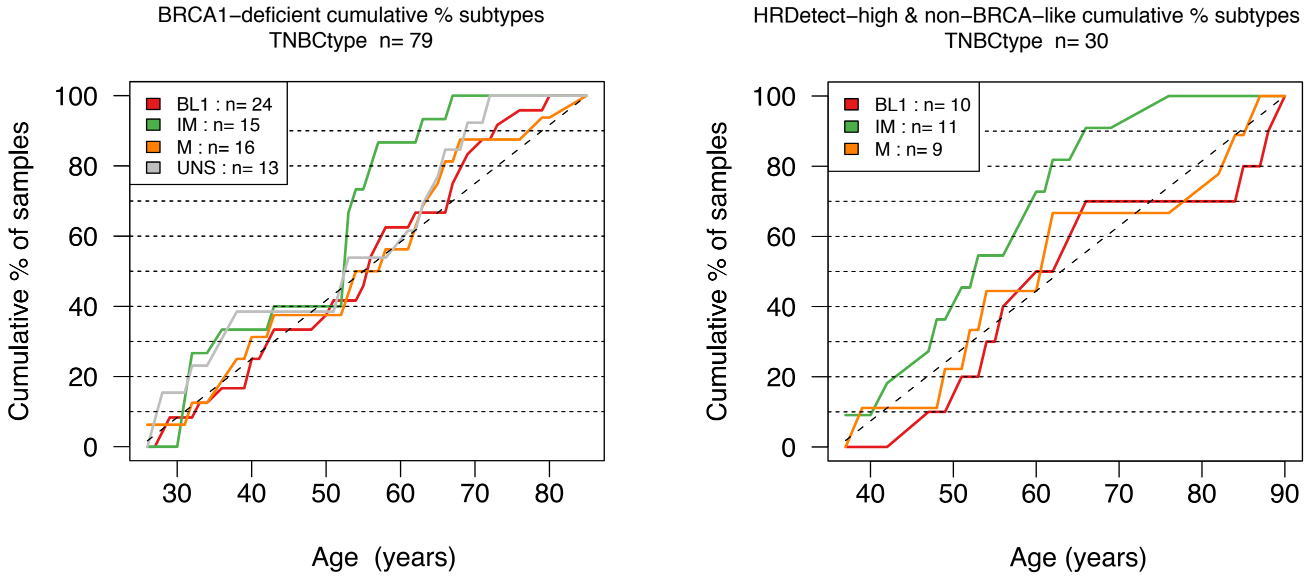
BRCA1−deficient cumulative % subtypes
TNBCtype n= 79
BL1 : n= 24
IM : n= 15
M : n= 16
UNS : n= 13
Cumulative % of samples
Age (years)
HRDetect−high & non−BRCA−like cumulative % subtypes
TNBCtype n= 30
BL1 : n= 10
IM : n= 11
M : n= 9
Cumulative % of samples
Age (years)

E)

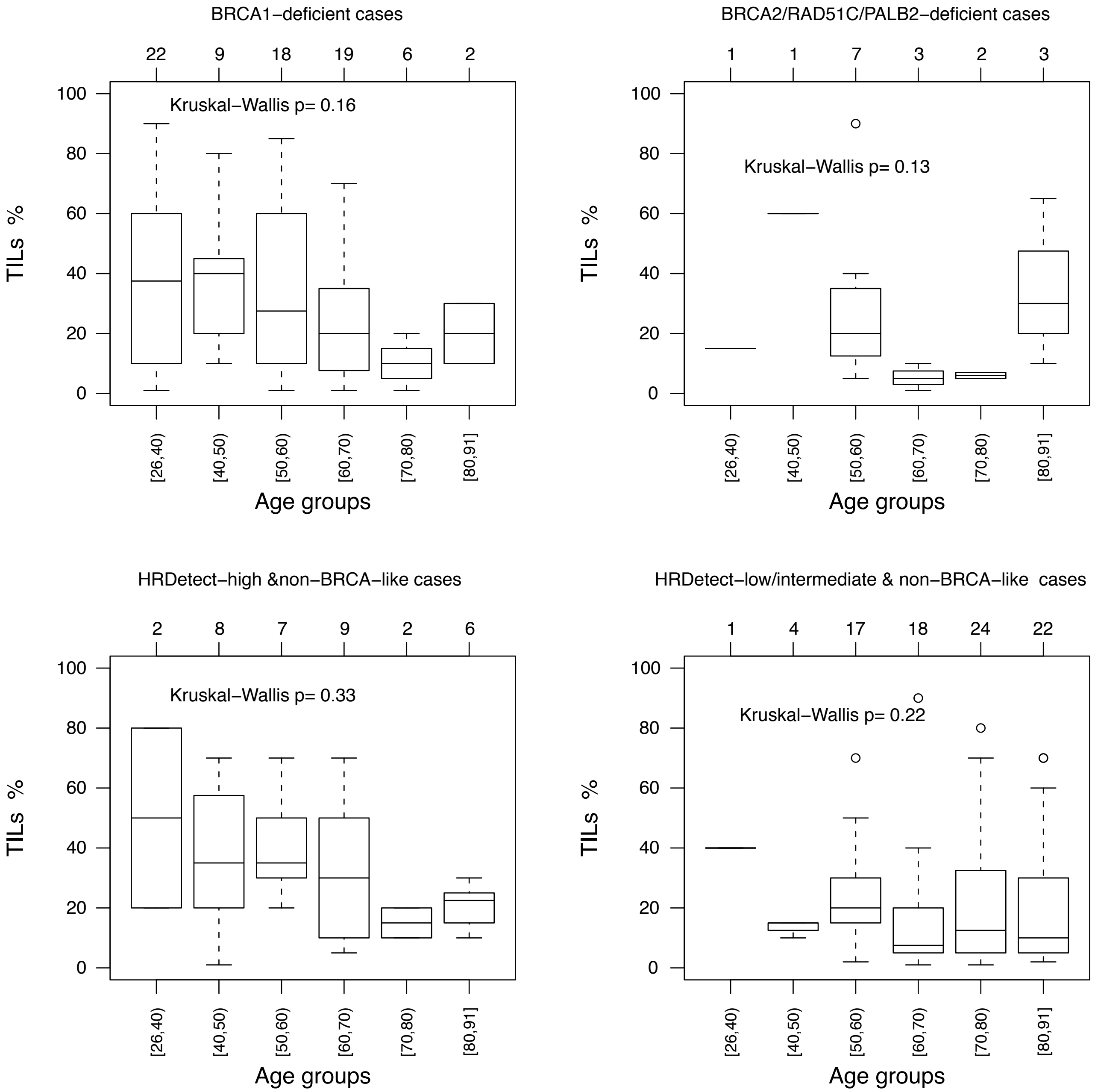

BRCA1−deficient cases
22 9 18 19 6 2
Kruskal−Wallis p= 0.16
100
80
60
40
20
0
TILs %
[26,40)
[40,50)
[50,60)
[60,70)
[70,80)
[80,91]
Age groups
BRCA2/RAD51C/PALB2−deficient cases
1 1 7 3 2 3
Kruskal−Wallis p= 0.13
TILs %
Age groups
HRDetect−high &non−BRCA−like cases
2 8 7 9 2 6
Kruskal−Wallis p= 0.33
TILs %
Age groups
HRDetect−low/intermediate & non−BRCA−like cases
1 4 17 18 24 22
Kruskal−Wallis p= 0.22
TILs %
Age groups
