# Supplementary material for: Molecular analyses of triple-negative breast cancer in the young and elderly
Source: Breast Cancer Res. 2021 Feb 10;23:20. doi: 10.1186/s13058-021-01392-0 (PMC7874480; doi:10.1186/s13058-021-01392-0)
Supplement: Supplementary file 6 — Additional file 6. A PDF file including supplementary Table 2 showing patterns of mutational and rearrangement signatures in the general Nik-Zainal et al. breast cancer cohort. [file 13058_2021_1392_MOESM6_ESM.pdf]

Supplementary Table 2. Patterns of mutational and rearrangement signatures in breast cancer.

	TNBC & HRDetect-high (n=88)	TNBC & HRDetect-low/inter (n=75)	ER+/HER2- & HRDetect-high (n=29)	ER+/HER2- & HRDetect-low/high (n=291)	PAM50 basal-like (n=55)	PAM50 luminal A (n=74)	PAM50 luminal B (n=111)
Insertions							
Age groups	NS	NS	NS	NS	NS	NS	NS
Linear Reg	NS	**NS* increase**	NS	NS	**Increase**	NS	NS
Deletions MH							
Age groups	NS	NS	NS	NS	NS	NS	NS
Linear Reg	NS	NS	NS	NS	NS	NS	NS
Deletions repeat							
Age groups	NS	NS	NS	NS	NS	NS	NS
Linear Reg	NS	NS	NS	NS	NS	NS	NS
Rearrangement Sig 1							
Age groups	NS	NS	NS	NS	NS	NS	NS
Linear Reg	NS	NS	NS	NS	NS	NS	NS
Rearrangement Sig 2							
Age groups	NS	NS	NS	NS	NS	NS	**NS***
Linear Reg	NS	NS	NS	NS	NS	NS	NS
Rearrangement Sig 3							
Age groups	NS	NS	NS	NS	NS	NS	NS
Linear Reg	NS	NS	NS	NS	**NS* increase**	NS	NS
Rearrangement Sig 4							
Age groups	NS	NS	NS	NS	NS	NS	NS
Linear Reg	NS	NS	NS	NS	NS	NS	NS
Rearrangement Sig 5							
Age groups	NS	NS	NS	NS	NS	NS	NS
Linear Reg	NS	NS	NS	NS	NS	NS	NS
Rearrangement Sig 6							
Age groups	NS	NS	NS	NS	NS	NS	NS
Linear Reg	NS	NS	NS	NS	NS	NS	NS
Mutational Sig 1							
Age groups	NS	NS	NS	NS	NS	NS	NS
Linear Reg	NS	NS	NS	NS	**NS* increase**	NS	NS
Mutational Sig 2							
Age groups	NS	NS	NS	NS	NS	NS	NS
Linear Reg	NS	NS	NS	NS	NS	NS	NS
Mutational Sig 3							
Age groups	NS	NS	NS	NS	NS	NS	NS
Linear Reg	NS	**NS* decrease**	NS	NS	**NS* decrease**	NS	NS
Mutational Sig 5							
Age groups	NS	NS	NS	**NS***	**NS***	**NS***	NS
Linear Reg	NS	**NS* Increase**	NS	**NS* Increase**	**Increase**	**Increase**	NS
Mutational Sig 6							
Age groups	NS	NS	NS	NS	NS	NS	NS
Linear Reg	NS	NS	NS	NS	NS	NS	NS
Mutational Sig 8							
Age groups	NS	NS	NS	NS	NS	NS	NS
Linear Reg	NS	NS	NS	NS	NS	NS	NS
Mutational Sig 13							
Age groups	NS	NS	NS	NS	NS	NS	NS
Linear Reg	NS	NS	NS	NS	NS	NS	NS
Mutational Sig 17							
Age groups	NS	NS	NS	NS	NS	NS	NS
Linear Reg	NS	NS	NS	NS	NS	NS	NS
Mutational Sig 18							
Age groups	NS	NS	NS	NS	NS	NS	NS
Linear Reg	NS	NS	NS	NS	NS	NS	NS

Mutational signatures 20 and 26 are excluded as they are mismatch repair deficient specific signatures.
Age groups: six age groups based on 10-year intervals; <40, 40-50, 50-60, 60-70, 70-80, and ≥80 years. P-value calculated by Kruskal-Wallis test and adjusted for multiple testing by Bonferroni adjustment by multiplication of 18 (representing the number of main features, rows, in the table tested per sample subgroup, to each p-value.
SIG: Bonferroni adjusted Kruskal-Wallis p-value <0.05
Linear Reg: Linear regression of variable versus patient age at diagnosis.
NS: non-significant with/without Bonferroni adjustment, p>0.05
NS*: non-significant after Bonferroni adjustment, significant without. The direction of the linear regression slope is provided.
Decrease: significant decrease with age by linear regression modelling, Bonferroni adjusted (original p-value multiplied by 18) p<0.05
Increase: significant increase with age by linear regression modelling, Bonferroni adjusted (original p-value multiplied by 18) p<0.05
