# Supplementary material for: Molecular analyses of triple-negative breast cancer in the young and elderly
Source: Breast Cancer Res. 2021 Feb 10;23:20. doi: 10.1186/s13058-021-01392-0 (PMC7874480; doi:10.1186/s13058-021-01392-0)
Supplement: Supplementary file 7 — Additional file 7. A PDF file with supplementary Figure 5 showing mutational and rearrangement signatures in 560 WGS analyzed breast cancers. [file 13058_2021_1392_MOESM7_ESM.pdf]

Supplementary Figure 5. Mutational and rearrangement signatures in 560 breast cancers. Tumors from Nik-Zainal et al. (Nature, 2016) were stratified into different groups: i) TNBC & HRDetect-high, ii) TNBC & HRDetect-low/intermediate, iii) ER-positive & HER2-negative (ER+/HER2-), iv) ER+/HER2- & HRDetect-high, v) ER+/HER2- & HRDetect-low/intermediate, vi) PAM50 basal-like, vii) PAM50 Luminal A, and viii) PAM50 Luminal B. For these groups proportions of different genome-wide patterns were compared versus patient age at diagnosis using both stratified age groups as defined below, and trend analysis using linear regression modelling of values as a function of age. For each trend analysis, a p-value and the slope (k) is reported. In total, for each patient subset 18 different features were tested as outlined in Supplementary Table S2, including individual mutational and rearrangement signatures. All p-values were corrected for multiple testing using Bonferroni adjustment by multiplication of p-values with 18. A corrected p-value <0.05 was considered significant.

(**A**) Significant associations in the Nik-Zainal cohort. All significant associations were observed using linear regression modelling (top panels). Corresponding boxplots for the stratified age groups are shown in lower panels. Both unadjusted and adjusted p-values are shown. (**B**) Proportions of mutational signature 1 (S1) and 5 (S5), proposed to be associated with age at diagnosis, across patient subgroups. (**C**) Fraction (proportion) of the genome altered by copy number alterations (CNA-FGA) or loss of heterozygosity (LOH-FGA) in different molecular subgroups of the Nik-Zainal et al. WGS data set stratified by age groups (box plots, left) or versus actual patient age (scatter plots, right). Scatter plot data was used for linear regression modelling using patient age as a continuous variable, providing a p-value, the regression line (red), and the slope coefficient in each plot.

For the age group definitions these are indicated as "[" meaning equal or greater than, ")" meaning smaller than, or "]" meaning smaller or equal than the value specified next to it.

A)

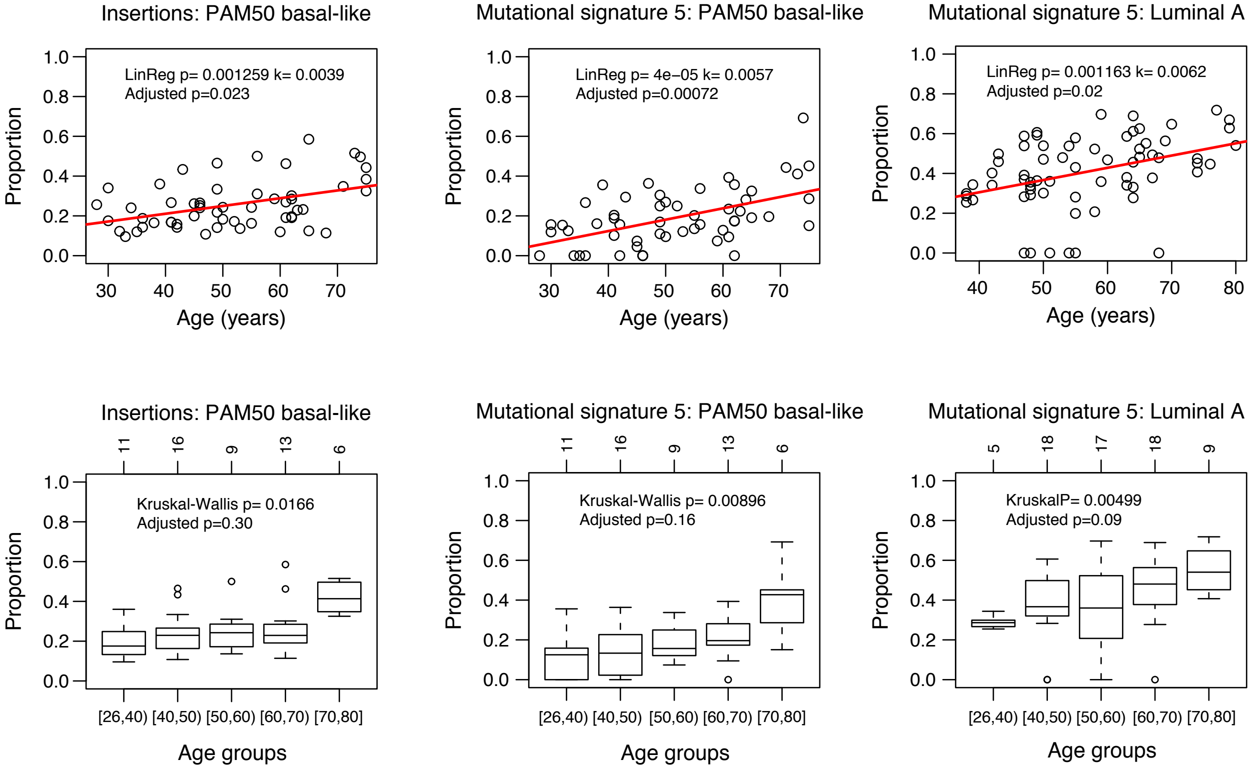

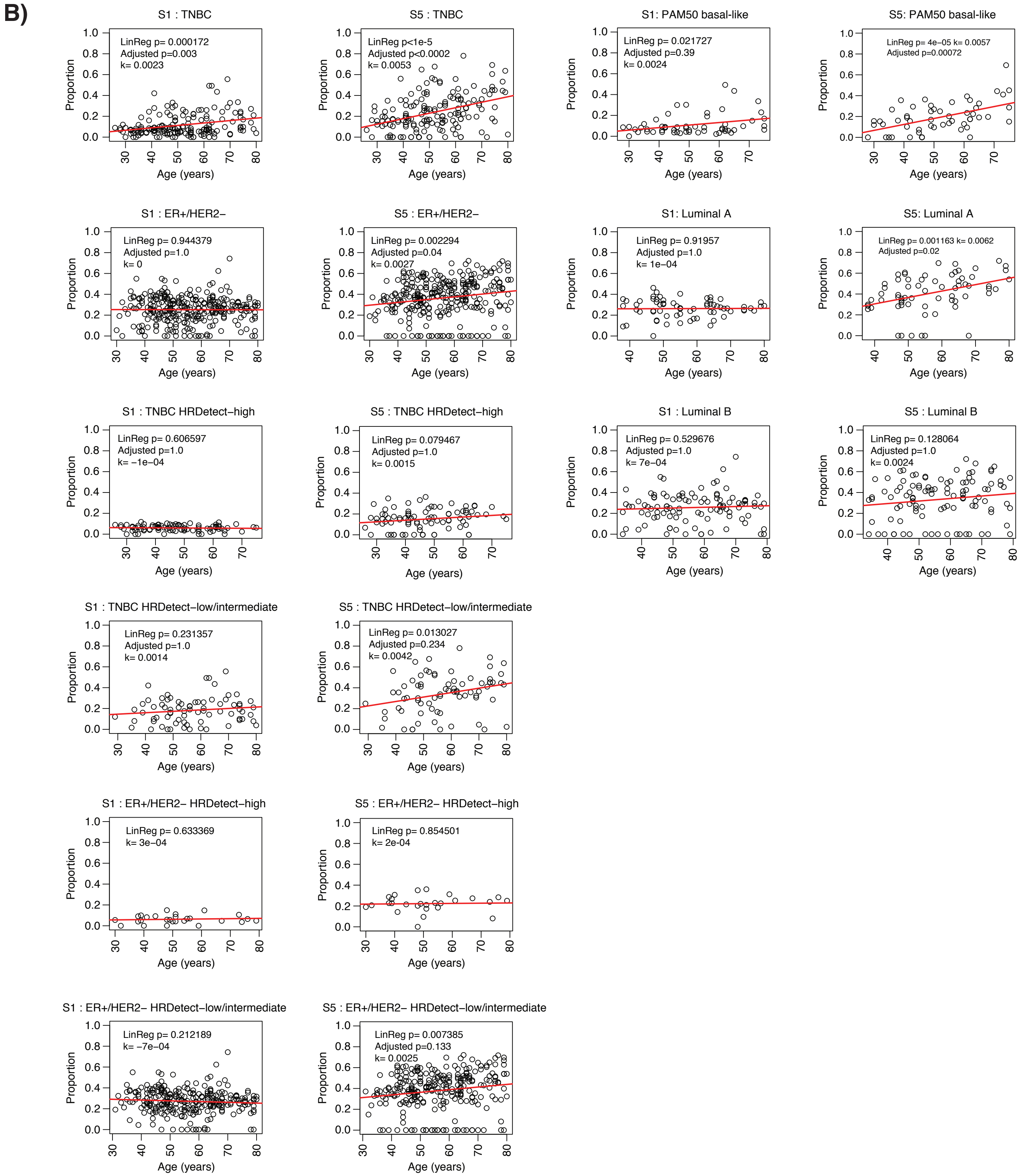

B)
S1 : TNBC
LinReg p= 0.000172
Adjusted p=0.003
k= 0.0023
S5 : TNBC
LinReg p<1e-5
Adjusted p<0.0002
k= 0.0053
S1: PAM50 basal-like
LinReg p= 0.021727
Adjusted p=0.39
k= 0.0024
S5: PAM50 basal-like
LinReg p= 4e-05 k= 0.0057
Adjusted p=0.00072
S1 : ER+/HER2−
LinReg p= 0.944379
Adjusted p=1.0
k= 0
S5 : ER+/HER2−
LinReg p= 0.002294
Adjusted p=0.04
k= 0.0027
S1: Luminal A
LinReg p= 0.91957
Adjusted p=1.0
k= 1e−04
S5: Luminal A
LinReg p= 0.001163 k= 0.0062
Adjusted p=0.02
S1 : TNBC HRDetect−high
LinReg p= 0.606597
Adjusted p=1.0
k= −1e−04
S5 : TNBC HRDetect−high
LinReg p= 0.079467
Adjusted p=1.0
k= 0.0015
S1 : Luminal B
LinReg p= 0.529676
Adjusted p=1.0
k= 7e−04
S5 : Luminal B
LinReg p= 0.128064
Adjusted p=1.0
k= 0.0024
S1 : TNBC HRDetect−low/intermediate
LinReg p= 0.231357
Adjusted p=1.0
k= 0.0014
S5 : TNBC HRDetect−low/intermediate
LinReg p= 0.013027
Adjusted p=0.234
k= 0.0042
S1 : ER+/HER2− HRDetect−high
LinReg p= 0.633369
k= 3e−04
S5 : ER+/HER2− HRDetect−high
LinReg p= 0.854501
k= 2e−04
S1 : ER+/HER2− HRDetect−low/intermediate
LinReg p= 0.212189
k= −7e−04
S5 : ER+/HER2− HRDetect−low/intermediate
LinReg p= 0.007385
Adjusted p=0.133
k= 0.0025
Proportion
Age (years)

C)

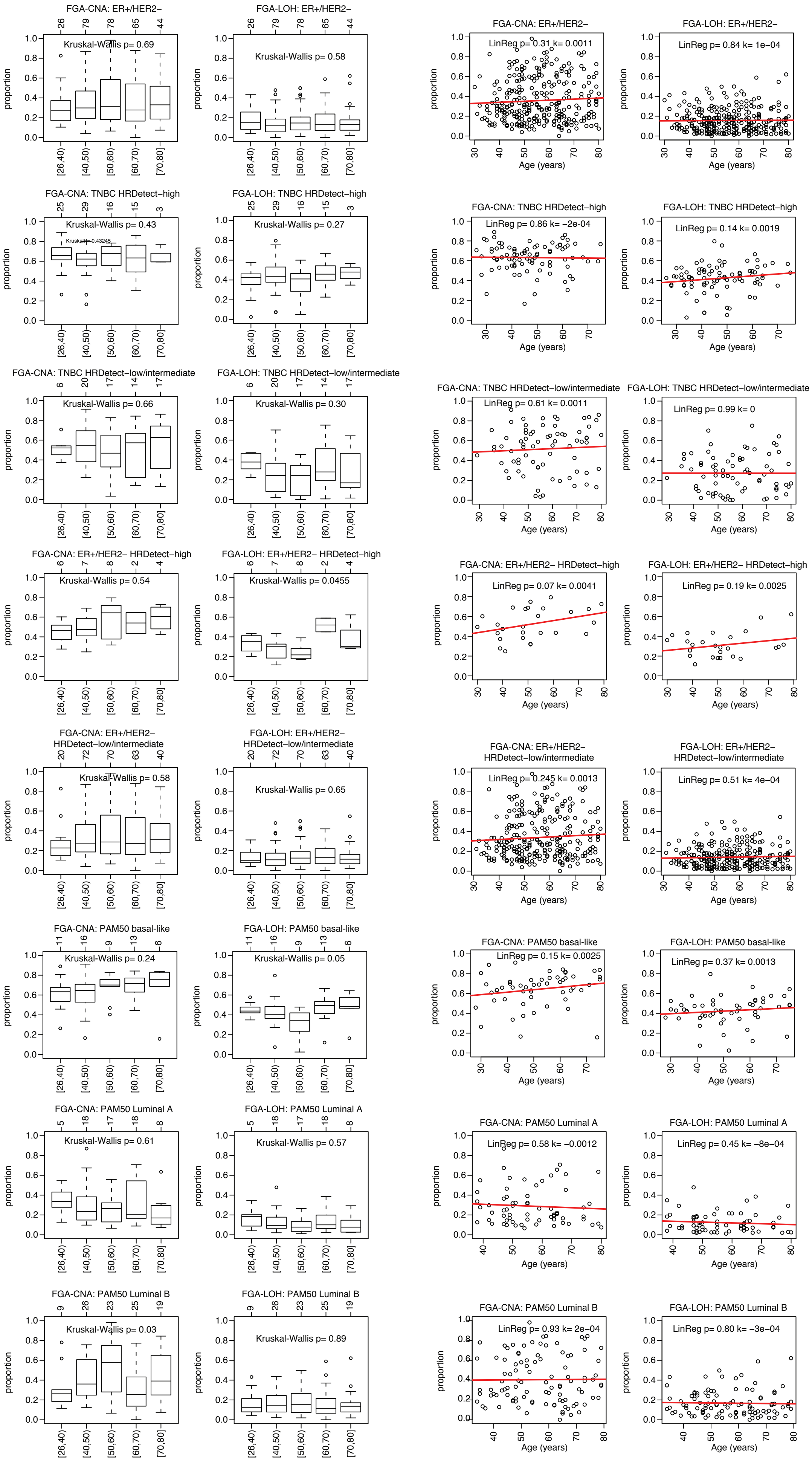

FGA-CNA: ER+/HER2−
Kruskal-Wallis p= 0.69
FGA-LOH: ER+/HER2−
Kruskal-Wallis p= 0.58
FGA-CNA: ER+/HER2−
LinReg p= 0.31 k= 0.0011
FGA-LOH: ER+/HER2−
LinReg p= 0.84 k= 1e−04
FGA-CNA: TNBC HRDetect−high
Kruskal-Wallis p= 0.43
FGA-LOH: TNBC HRDetect−high
Kruskal-Wallis p= 0.27
FGA-CNA: TNBC HRDetect−high
LinReg p= 0.86 k= −2e−04
FGA-LOH: TNBC HRDetect−high
LinReg p= 0.14 k= 0.0019
FGA-CNA: TNBC HRDetect−low/intermediate
Kruskal-Wallis p= 0.66
FGA-LOH: TNBC HRDetect−low/intermediate
Kruskal-Wallis p= 0.30
FGA-CNA: TNBC HRDetect−low/intermediate
LinReg p= 0.61 k= 0.0011
FGA-LOH: TNBC HRDetect−low/intermediate
LinReg p= 0.99 k= 0
FGA-CNA: ER+/HER2− HRDetect−high
Kruskal-Wallis p= 0.54
FGA-LOH: ER+/HER2− HRDetect−high
Kruskal-Wallis p= 0.0455
FGA-CNA: ER+/HER2− HRDetect−high
LinReg p= 0.07 k= 0.0041
FGA-LOH: ER+/HER2− HRDetect−high
LinReg p= 0.19 k= 0.0025
FGA-CNA: ER+/HER2− HRDetect−low/intermediate
Kruskal-Wallis p= 0.58
FGA-LOH: ER+/HER2− HRDetect−low/intermediate
Kruskal-Wallis p= 0.65
FGA-CNA: ER+/HER2− HRDetect−low/intermediate
LinReg p= 0.245 k= 0.0013
FGA-LOH: ER+/HER2− HRDetect−low/intermediate
LinReg p= 0.51 k= 4e−04
FGA-CNA: PAM50 basal-like
Kruskal-Wallis p= 0.24
FGA-LOH: PAM50 basal-like
Kruskal-Wallis p= 0.05
FGA-CNA: PAM50 basal-like
LinReg p= 0.15 k= 0.0025
FGA-LOH: PAM50 basal-like
LinReg p= 0.37 k= 0.0013
FGA-CNA: PAM50 Luminal A
Kruskal-Wallis p= 0.61
FGA-LOH: PAM50 Luminal A
Kruskal-Wallis p= 0.57
FGA-CNA: PAM50 Luminal A
LinReg p= 0.58 k= −0.0012
FGA-LOH: PAM50 Luminal A
LinReg p= 0.45 k= −8e−04
FGA-CNA: PAM50 Luminal B
Kruskal-Wallis p= 0.03
FGA-LOH: PAM50 Luminal B
Kruskal-Wallis p= 0.89
FGA-CNA: PAM50 Luminal B
LinReg p= 0.93 k= 2e−04
FGA-LOH: PAM50 Luminal B
LinReg p= 0.80 k= −3e−04
proportion
Age (years)
